# Discovery and Synthetic Applications of a NAD(P)H-Dependent Reductive Aminase from *Rhodococcus erythropolis*

**DOI:** 10.1021/acscatal.4c04935

**Published:** 2024-12-16

**Authors:** Ewald P. J. Jongkind, Jack Domenech, Arthur Govers, Marcel van den Broek, Jean-Marc Daran, Gideon Grogan, Caroline E. Paul

**Affiliations:** † Department of Biotechnology, Delft University of Technology, van der Maasweg 9, 2629 HZ Delft, The Netherlands; ‡ York Structural Biology Laboratory, Department of Chemistry, University of York, Heslington, York YO10 5DD, United Kingdom

**Keywords:** Biocatalysis, chiral amines, cofactor specificity, imine reductases, reductive amination

## Abstract

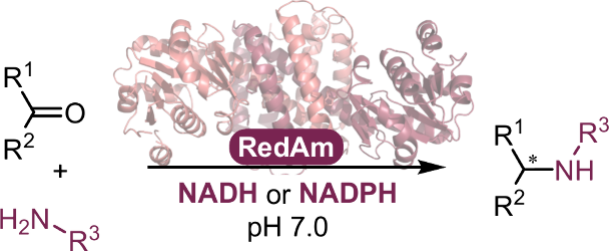

Reductive amination is one of the most synthetically direct routes to access chiral amines. Several Imine Reductases (IREDs) have been discovered to catalyze reductive amination (Reductive Aminases or RedAms), yet they are dependent on the expensive phosphorylated nicotinamide adenine dinucleotide cofactor NADPH and usually more active at basic pH. Here, we describe the discovery and synthetic potential of an IRED from *Rhodococcus erythropolis* (*Ryt*RedAm) that catalyzes reductive amination between a series of medium to large carbonyl and amine compounds with conversions of up to >99% and 99% enantiomeric excess at neutral pH. *Ryt*RedAm catalyzes the formation of a substituted γ-lactam and *N*-methyl-1-phenylethanamine with stereochemistry opposite to that of fungal RedAms, giving the (*S*)-enantiomer. This enzyme remarkably uses both NADPH and NADH cofactors with *K*_M_ values of 15 and 247 μM and turnover numbers *k*_cat_ of 3.6 and 9.0 s^–1^, respectively, for the reductive amination of hexanal with allylamine. The crystal structure obtained provides insights into the flexibility to also accept NADH, with residues R35 and I69 diverging from that of other IREDs/RedAms in the otherwise conserved Rossmann fold. *Ryt*RedAm thus represents a subfamily of enzymes that enable synthetic applications using NADH-dependent reductive amination to access complementary chiral amine products.

## Introduction

**Figure 1:**
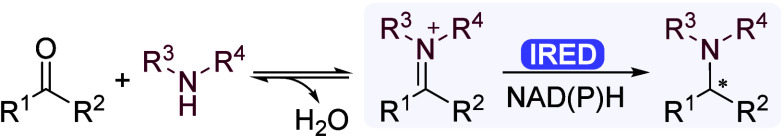
Reductive amination of carbonyls with alkylated amines and imine reduction (in light blue), catalyzed by IREDs.

Chiral amines are valuable motifs for applications in the pharmaceutical and agrochemical industries, representing ca. 40% of FDA-approved small molecule drugs.^1,2^ Besides metallo-^3^ and organocatalysts,^4^ biocatalysts have been used for the asymmetric production of chiral amines from prochiral substrates since the early 2000s.^5−11^ Within the oxidoreductase class of enzymes,^12,13^ herein, we focus on the family of nicotinamide adenine dinucleotide (phosphate) NAD(P)-dependent imine reductases (IREDs) that catalyze the asymmetric reduction of imines to amines^14,15^ and its subfamily of reductive aminases (RedAms) that catalyze the full reductive amination reaction from carbonyls and alkyl amines (Figure 1),^16−19^ in contrast to amine dehydrogenases, which mostly accept ammonia and methylamine.^10,20^ IREDs are homodimeric enzymes that contain a Rossmann-fold and prefer NADPH over NADH.^21^ In general, their active site harbors a negatively charged residue, usually aspartate, which has been proposed to stabilize the positively charged iminium substrate, and a proton donor such as tyrosine is often present as well.^22^ IREDs show the highest activity toward imine substrates at neutral pH;^23^ however, with imines being prone to hydrolysis in water,^24,25^ a carbonyl substrate with an excess of amine donor at a basic pH is usually applied to favor imine formation. The discovery of a “Reductive Aminase” from *Aspergillus oryzae* (*Asp*RedAm) and other IREDs enabled some reductive aminations to be performed with one molar equivalent of amine donor at neutral pH (Figure 1).^18,19,26,27^

Several IREDs with the ability to enable reductive aminations at equimolar concentrations of carbonyl and amine at neutral pH have been identified in fungi^22,28^ and bacteria^19,30^ and from wide panels of IREDs that were screened for this property at neutral pH.^18,27^ While the active sites in fungal enzymes contains several conserved amino acids,^26^ RedAm activity has also been demonstrated in enzymes with a range of active site residues.^29^ In most cases, however, the majority of enzymes favor phosphorylated cofactor NADPH over NADH.

In this work, we describe the discovery and characterization of a bacterial enzyme from *Rhodococcus erythropolis* (*Ryt*RedAm), which accepts both NADPH and NADH as nicotinamide cofactors. The gene encoding for this enzyme was discovered based on a sequence alignment with *Asp*RedAm from a database from the *Rhodococcus* genome via a Hidden-Markov Model (HMM).^28^

## Materials and Methods

### Expression and Enzyme Purification

The thermostable glucose dehydrogenase double mutant E170K_Q252L from *Bacillus subtilis*, *Bs*GDH E170K_Q252L, was previously produced and purified.^29^ Lyophilized plasmids of the selected sequences from EnzymeMiner and the *Rhodococcus* sequences cloned in pET-28a(+) were ordered and received from SynBio Technologies (not codon optimized, with an *N*-terminal His-tag). The *Asp*RedAm plasmid was provided by N. J. Turner (University of Manchester). *E. coli* BL21(DE3) chemically competent cells were transformed with the vector containing the listed genes unless stated otherwise (see SI 1.1). The *Asp*RedAm gene was transformed in *E. coli* BL21(DE3), C43(DE3), or BL21 Gold(DE3) competent cells (*Asp*RedAm production was significantly improved using the *E. coli* BL21 Gold(DE3) strain (SI Figure S2)). The transformed cells were grown on selective LB-agar plates (50 μg/mL kanamycin) overnight at 37 °C. Terrific broth (TB)-medium (500 mL in a 2 L baffled flask) was inoculated with 1% v/v overnight preculture and incubated at 37 °C. Expression was induced with 0.5 mM isopropyl β-d-1-thiogalactopyranoside (IPTG) at an OD_600_ of 0.6–0.8, followed by overnight incubation at 20 °C. Cells were harvested (17,000*g*, 20 min, 4 °C) and stored at −80 °C. Purification by immobilized-metal affinity chromatography (IMAC) was carried out. The cell pellets were thawed and suspended in 5 mL/g_wcw_ binding buffer of 50 mM Tris-HCl, pH 8.0, 300 mM NaCl, 1 mM MgCl_2_, and 30 mM imidazole, and a spatula tip of DNaseI, MgCl_2_, and lysozyme and a pill of cOmplete mini EDTA-free protease inhibitor were added. Three g_wcw_ of cells were passed through a cell disrupter (22 kpsi) and centrifuged (32,000*g*, 30 min, 4 °C). The cell-free extract (CFE) was filter loaded on a Ni-NTA 5 mL HisTrap FF crude column with a Bio-Rad NGC Chromatography system with an elution buffer of 50 mM Tris-HCl, pH 8.0, 300 mM NaCl, 1 mM MgCl_2_, and 300 mM imidazole. Pooled elution fractions were passed through a PD10 desalting column, flash frozen in liquid nitrogen, and stored at −80 °C. Enzyme concentration was determined by a bicinchoninic acid assay. For further details see SI Section 1.

### Activity Assays

For specific activity measurements, 4 mL UV grade poly(methyl methacrylate) (PMMA) plastic cuvettes were used to monitor the decrease of NAD(P)H at a wavelength of 340 nm with the extinction coefficient ε_340 nm_ = 6220 M^–1^cm^–1^, on a Cary 60 UV–vis spectrophotometer. Carbonyl substrates were prepared fresh as a 1 M stock solution in DMSO. Amines were prepared fresh in 100 mM KP_i_ buffer pH 7.0 and titrated with a solution of 6 M HCl to adjust the pH to 7.0. NAD(P)H stock solutions were prepared fresh in buffer as a 10 mM concentration, confirmed by UV spectrophotometry at 340 nm. For buffers at different pHs the following salts were used: sodium acetate-HCl pH 5, pyridine-HCl pH 5.5, KPi pH 6–8, MOPS-NaOH pH 6.5–7.5, Tris-HCl pH 7.5–9, and glycine-NaOH pH 9.5–10. Buffer pHs were thermodynamically corrected using the Buffer Calculator.^30^ Further details are in SI Section 3.

### Biotransformations

Reactions were performed in GC glass vials with 100 mM KP_i_ buffer, pH 7.0, 10 mM carbonyl substrate, 10–1000 mM amine donor, 0.2 mM NADP^+^, 30 mM glucose (Glc), 10 U/mL *Bs*GDH, 0.5 mg/mL purified *Ryt*RedAm, and 0.5 mL total reaction volume. Reactions were stirred at 500 rpm and 30 °C for 24 h on an Eppendorf Thermomixer C. To quench the reaction, 0.4 mL of 10 M NaOH was added, and the reaction mixture was extracted with 0.5 mL EtOAc, vortexed and centrifuged (10,000*g*, 1 min). The isolated organic layer was dried over anhydrous MgSO_4_, centrifuged (10,000*g*, 1 min), decanted to a GC vial, and injected onto the GC-FID. Control reactions were ran in the same conditions but without *Ryt*RedAm. For further details, see SI Sections 4 and 6.

### Crystallization

Crystals of both apo-*Ryt*RedAm and the ADP-2′-ribose phosphate (ADP-2RP) complex were grown using *Ryt*RedAm concentrated to 35 mg/mL in 50 mM HEPES buffer at pH 7.0 containing 300 mM NaCl. The structures of *Ryt*RedAm and *Ryt*RedAm-ADP-2RP have been deposited in the Protein Databank (PDB) with accession codes 9FM8 and 9FM7, respectively. Further details can be found in SI Section 1.6.

## Results and Discussion

### Discovery of *Ryt*RedAm

**Figure 2:**
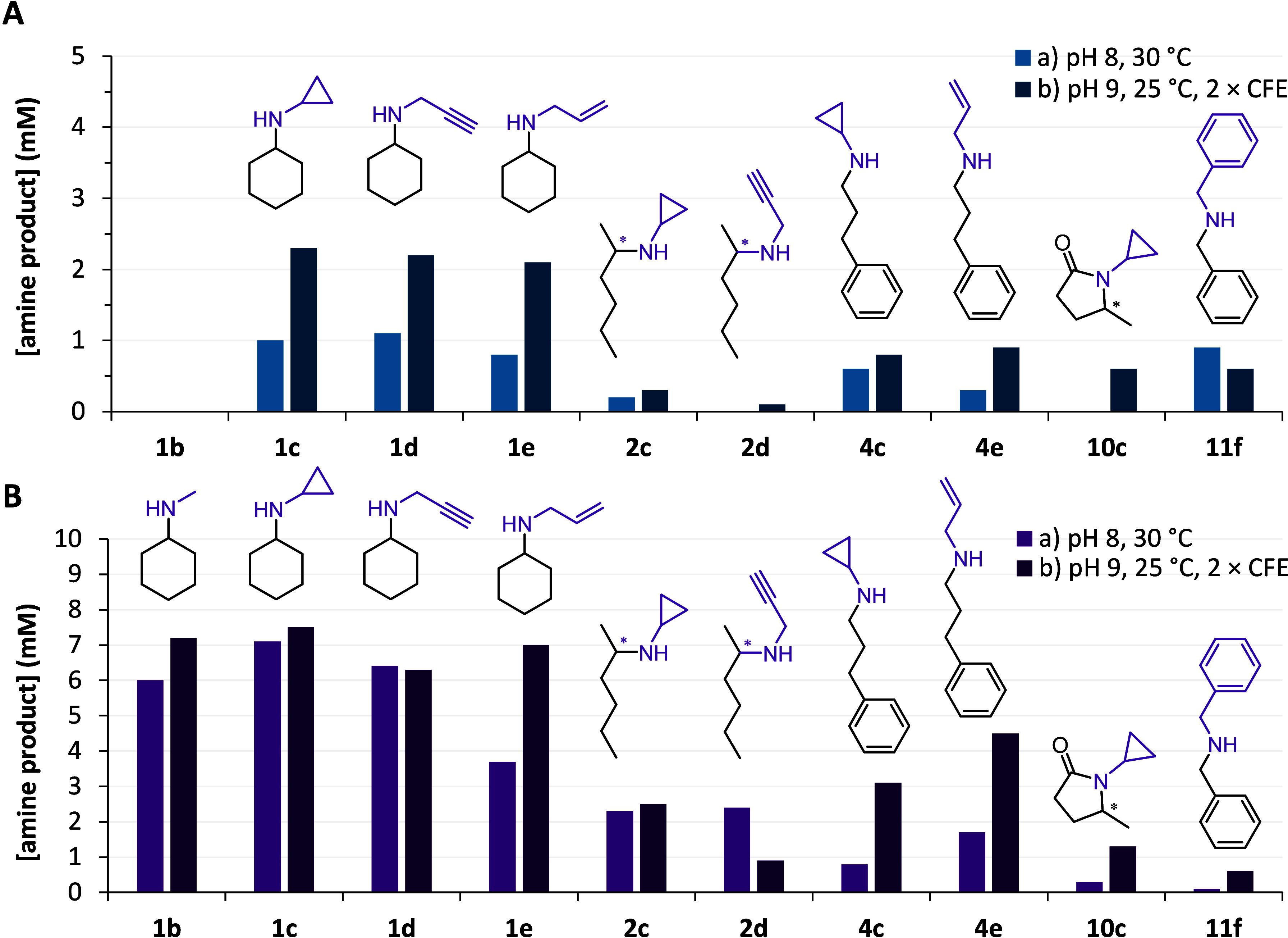
CFE-catalyzed reductive amination under reaction conditions (a) and (b) of (A) *Pih*RedAm and (B) *Shy*RedAm. *Absolute configuration aligned with that of *Asp*RedAm. Conditions: (a) 100 mM Tris-HCl, pH 8.0, 0.2 mM NADP^+^, 12 mM Glc, 6 U/mL GDH, 50 μL of CFE, 10 mM carbonyl, 1–20 equiv amine: **1c**, **1d**, **1e**, **2c**, and **2d** with a 1:1 ratio, **1b** 1:2 ratio, **4c**, **4e**, and **11f** 1:4 ratio, **10c** 1–20 ratio, 30 °C, 500 rpm, 24 h; (b) 100 mM Tris-HCl, pH 9.0, 0.4 mM NADP^+^, 30 mM Glc, 10 U/mL GDH, 100 μL of CFE, 10 mM carbonyl, 1–20 equiv amine as above, 25 °C, 500 rpm, 24 h.

We started our search for bacterial reductive aminases with *Asp*RedAm (accession number Q2TW47) as the query sequence, based on its three catalytic active site residues N93, D169, and Y177 and the other three residues W210, M239, and Q240 reported for substrate binding (Table 1).^26^ With the National Center for Biotechnology Information (NCBI) database, we used two bioinformatics tools. First, EnzymeMiner identified putative sequences by filtering hits containing either all six or only the three catalytic residues.^31^ Several enzyme sequences were selected for production; some did not express, or the protein precipitated after overexpression. Of the seven soluble enzymes obtained (SI Table S1), five gave activity with CFEs and three were screened for conversions with different substrates (Figure 2, SI Figures S9–S11 and S14). *Pih*RedAm (from *Paenibacillus ihbetae*), *Shy*RedAm (from *Streptomyces hygroscopicus*), and *Bac*RedAm (from *Bacillus* sp. J13) gave low to high conversions. *Shy*RedAm was the most promising, as it converted ketones such as cyclohexanone (with cyclopropylamine, propargylamine, and allylamine) and 2-hexanone (with cyclopropylamine and propargylamine) in a 1:1 ratio of carbonyl:amine (Figure 2). *Shy*RedAm displayed the same (*R*)-enantioselectivity as with *Asp*RedAm (**2c**, **2d**, **10c**) and accepted methyl amine to produce *N*-methylcyclohexylamine **1b**. *Pih*RedAm also displayed the same enantiopreference as *Asp*RedAm for **10c** but with higher selectivity, >99% *ee*.

**Table 1:** Active Site Residues of Sequences Obtained from the *Rhodococcus* Database and Corresponding Positions of Hit Sequences Compared with the Sequence of *Asp*RedAm

RedAm	93	169	177	210	239	240	Identity	Solubility
*Asp*RedAm	N	D	Y	W	M	Q	100	0.76
*Roc*RedAm	T	D	Y	F	L	E	39	0.47
*Rop*RedAm	T	D	Y	F	F	T	35	0.13
*Noc*RedAm	S	Y	H	F	M	M	32	0.34
*Ryt*RedAm	T	D	F	W	V	Y	31	0.36

In parallel, we used the basic local alignment search tool (BLAST)^32^ to download all hits with a sequence identity of more than 50%, cumulating to 100 sequences. A multiple sequence alignment was extrapolated from a multiple sequence comparison by log-expectation (MUSCLE),^33^ and a HMM database was created with the HMMer tool, resulting in a database with conserved domains of 101 protein sequences. With the HMM, we searched in the genome databases of 43 different *Rhodococcus* strains as reported in Busch et al.^28^ The output list of sequences was ranked based on the E-value. We selected the most significant hits with the following restrictions: the host organism should be known, and each sequence should originate from a different bacterial organism. Four hits from *Rhodococcus rhodochrous* (*Roc*RedAm), *Rhodococcus opacus* (*Rop*RedAm), *Nocardia seriolae* (*Noc*RedAm), and *Rhodococcus erythropolis* (*Ryt*RedAm) were selected based on the six active site residues from the *Asp*RedAm query sequence (Table 1, sequence identity and solubility factor predicted by SoluProt (between 0 and 1)^34^). Two of the three catalytic residues in the *Asp*RedAm active site D169 and Y177 are similar to the hit sequences except for *Noc*RedAm. The asparagine N93, shown to have a role in the catalytic activity,^22^ is replaced by a threonine or serine. The substrate active site residues at positions 210, 239, and 240 showed variances that could possibly result in a different substrate scope.

The potential bacterial RedAms were recombinantly produced in *E. coli*, and their activity was tested with hexanal and allylamine at neutral pH (SI Figures S4 and S10). The gene encoding for *Noc*RedAm did not overexpress, and *Roc*RedAm resulted in insoluble protein (SI Figure S4). *Rop*RedAm was poorly active at pH 7, whereas *Ryt*RedAm showed highly promising activity from the CFE, more than with *Shy*RedAm (SI Figures S10–S11), and thus was further characterized.

### *Ryt*RedAm Characterization

**Figure 3:**
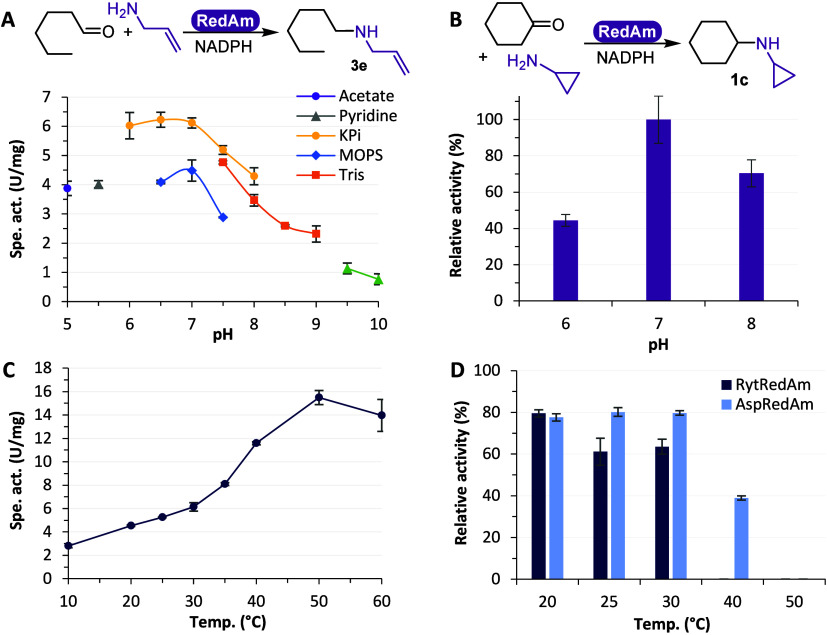
Effect of the pH and temperature on activity. (A) Conditions: 50 mM buffer of acetate pH 5.0 (purple ●), pyridine pH 5.5 (gray ▲), KPi pH 6.0–8.0 (yellow ●), MOPS-NaOH pH 6.5–7.5 (blue ◆), Tris-HCl pH 7.5–9.0 (orange ■), glycine-NaOH pH 9.5–10.0 (green ▲), 10 mM hexanal, 100 mM allylamine, 1% v/v DMSO, 0.2 mM NADPH, *Ryt*RedAm, 25 °C. (B) Conditions: 50 mM KP_i_ pH 6.0–8.0, 10 mM cyclohexanone, 100 mM cyclopropylamine, 0.2 mM NADPH, *Ryt*RedAm, 25 °C; 100% corresponds to 23 mU/mg. (C) Conditions: 100 mM KPi pH 7.0, 10 mM hexanal, 100 mM allylamine, 1% v/v DMSO, 0.2 mM NADPH, *Ryt*RedAm, 10–60 °C. (D) Conditions: *Ryt*RedAm or *Asp*RedAm incubated 1 h in 100 mM KP_i_ pH 7.0 at 20–50 °C, activity measured at 30 °C with 10 mM hexanal, 100 mM allylamine, 1% v/v DMSO, 0.2 mM NADPH; 100% corresponds to 6.7 U/mg for *Ryt*RedAm and 0.97 U/mg for *Asp*RedAm. Average of duplicates.

*Ryt*RedAm was purified by affinity chromatography in high yield with 250 mg of pure *Ryt*RedAm/6 g of wet cell weight (SI Figure S5). *Ryt*RedAm was further characterized in different buffer salts, measuring activity with hexanal and allylamine across a pH range of 5.0–10.0 (Figure 3A). MOPS-NaOH buffer caused lower activity compared to KP_i_ and Tris-HCl. KP_i_ buffer showed the highest activity within the pH range of 6.0–7.0 (6.1 U/mg at 25 °C) and was therefore used as a reaction and storage buffer. The specific activity was systematically higher at neutral pH than at pH 9 by a factor of 0.4. In comparison, *Asp*RedAm has a 0.8-fold lower activity at pH 7 compared with pH 9.^26^ Certain IREDs were also reported to have a broad pH range and be active at neutral pH, indicating the formation of the iminium.^23,27,35^ Therefore, we hypothesize that *Ryt*RedAm acts as a reductive aminase at neutral pH with a 1:1 ratio for several carbonyl:amine partners (shown further below in Figure 7). The activity at different pH values for cyclohexanone and cyclopropylamine was also measured and provided the highest activity at pH 7 (Figure 3B).

With the ideal neutral pH in hand, *Ryt*RedAm activity was measured at varying temperatures (Figure 3C). The activity increased along with higher temperatures, reaching a maximum at 50 °C and dropping at 60 °C. In terms of stability, *Ryt*RedAm displayed 20–35% loss of activity after a 1 h incubation at 20–30 °C and complete loss of activity at 40 °C (Figure 3D). Therefore, a temperature of 30 °C was used for further reaction screenings.

**Figure 4:**
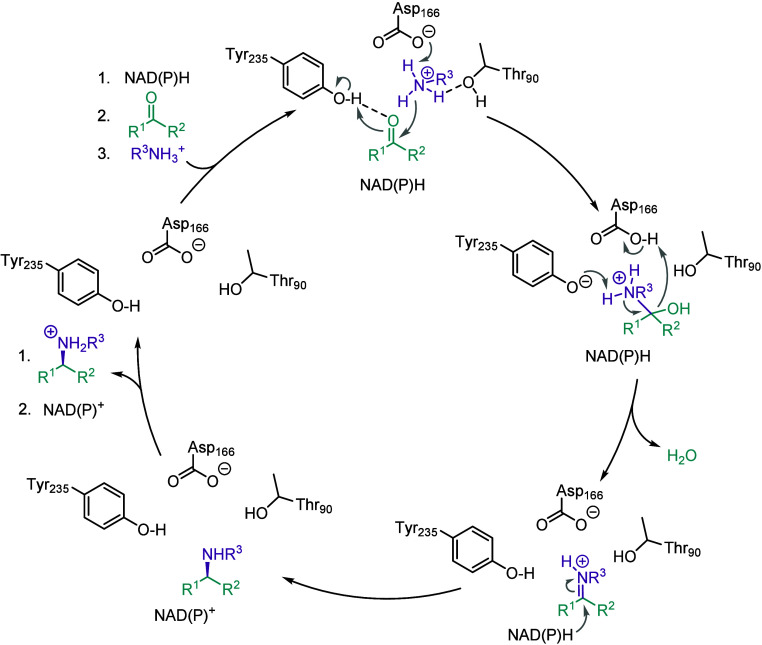
Proposed reaction mechanism of *Ryt*RedAm for reductive amination, involving key residues T90, D166, and Y235.

Considering the key amino acids involved in the active site, we propose the following mechanism based on previous observations with *Asp*RedAm (Figure 4).^22^ Y235 would be involved as a proton donor, while D166 and T90 coordinate the protonated amine donor. A nucleophilic attack of the amine on the carbonyl substrate can lead to an iminium ion intermediate and the release of water. NAD(P)H then can reduce the iminium intermediate, resulting in the amine formation and release.

### Evaluation of *Ryt*RedAm Substrate Scope

**Figure 5:**
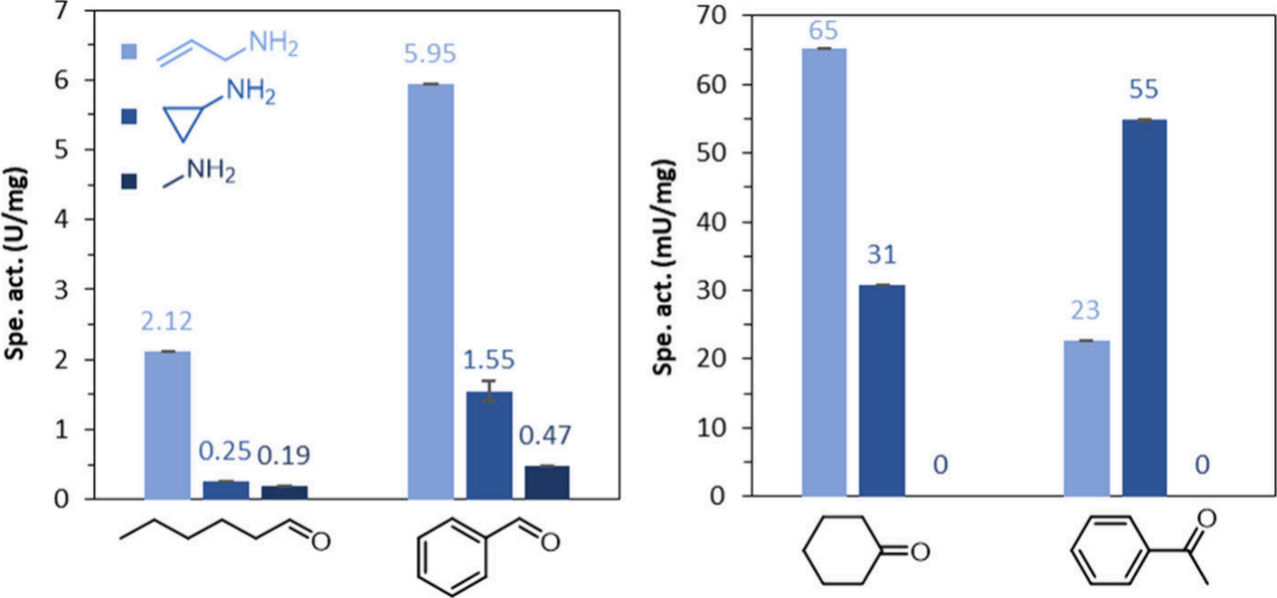
Specific activity of *Ryt*RedAm toward aldehyde (left) or ketone (right) substrates with 10 molar equiv of amine donor. Conditions: 100 mM KPi pH 7.0, 10 mM hexanal, benzaldehyde, cyclohexanone, or acetophenone, 100 mM allylamine, cyclopropylamine, or methylamine, 0.2 mM NADPH, *Ryt*RedAm, 30 °C. Average of duplicates.

*Ryt*RedAm activity was the highest with aldehydes, hexanal, and benzaldehyde, coupled with allyl amine, followed by cyclopropyl amine and methylamine (Figure 5). With ketones, cyclohexanone and acetophenone gave activities of 23–65 mU/mg with allyl amine and cyclopropyl amine and no observable initial activity with methylamine.

**Figure 6:**
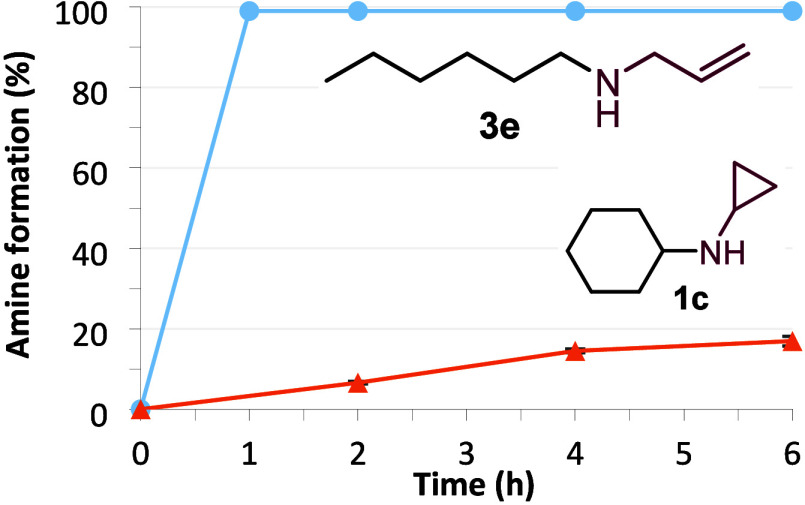
Reductive amination of hexanal with allylamine (blue ●) and cyclohexanone with cyclopropylamine (orange ▲). Conditions: 100 mM KPi pH 7.0, 30 mM Glc, 6 U/mL *Bs*GDH E170K_Q252L, 10 mM carbonyl, 25 mM amine, 0.2 mM NADP^+^, 0.25 mg/mL *Ryt*RedAm, 500 rpm, 30 °C, 24 h. Average of duplicates.

The conversion rate of *Ryt*RedAm with two different substrate-amine combinations over time showed full conversion in under 30 min for the most favored combination of hexanal and allylamine (Figure 6). The reductive amination of cyclohexanone with cyclopropylamine to form **1c** was clearly slower over time. A scale-up with 50 mM hexanal and 100 mM allylamine afforded 65 mg of pure **3e** (52% isolated yield), as confirmed by NMR (SI Figures S47–S48).

**Figure 7:**
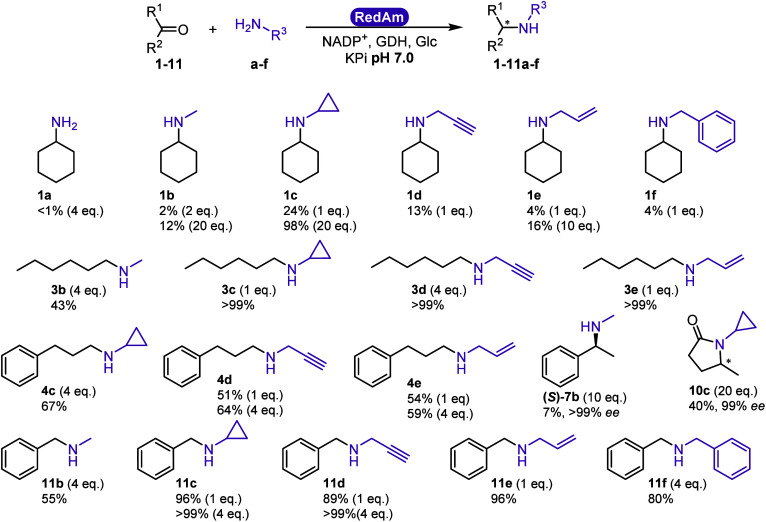
Reductive amination of carbonyl:amine combinations catalyzed by RytRedAm. Conditions: 100 mM KPi pH 7.0, 0.5 mg/mL RytRedAm, 30 mM Glc, 6 U/mL *Bs*GDH E170K_Q252L, 10 mM carbonyl, 10–200 mM amine (equivalents of amine donor in parentheses), 0.2 mM NADP^+^, 500 rpm, 30 °C, 24 h. Average of duplicates. *: Absolute configuration not assigned, opposite to that obtained with AspRedAm.

Bioconversions were carried out with a panel of carbonyl substrates and amine donors to establish a wider substrate scope (Figure 7, SI). Starting from 10 mM carbonyl, excellent conversions were obtained with aldehydes such as hexanal (>99% **3c**–**e**) and benzaldehyde (89–99% **11c**–**11e**) with only one molar equivalent of amine donor and moderate to high conversions, with hydrocinnamaldehyde (59–67% **4c**–**e**), combined with four molar equivalent of the best amine donors cyclopropylamine, propargylamine, and allylamine. Methyl amine gave 43–55% conversion with aldehydes (**3b**, **11b**) and 7–12% with ketones (**1b**, **7b** with >99% *ee S*). Benzyl amine gave 80% conversion with benzaldehyde (**11f**) and 4% conversion with cyclohexanone (**1f**). Reductive amination was not observed with ammonia (**1a**, SI Figure S15), nor with 2-hexanone (SI Figure S8).

Previous work showed that more equivalents of amine donor increased conversion. A conversion of 98% **1c** was achieved with 20 equiv cyclopropyl amine, compared with 24% when using equimolar amounts (see SI Figure S15). Imine reduction with 2-methyl-1-pyrroline was attempted, but less than 2% conversion was obtained after 24 h (SI Figure S39). This displays the lack of IRED activity in contrast with the reported *Asp*RedAm *v*_max_ of 0.24 U/mg.^26^

Interestingly, ethyl levulinate combined with cyclopropylamine produced the chiral γ-lactam product 1-cyclopropyl-5-methyl-2-pyrrolidinone **10c**. A scale-up provided 11 mg of pure **10c** (confirmed by NMR, SI Figures S50–51) with 95% *ee*, the opposite enantiomer to that formed by *Asp*RedAm, which provides only 74% *ee* (SI Figures S33 and S49). The opposite enantioselectivity is also seen with **7b** (>99% *ee* (*R*) for *Asp*RedAm and (*S*) for *Ryt*RedAm, SI Figure S30). This complementarity enables further opportunities for product scope with RedAms, which are otherwise engineered to access the desired enantiomer.^36^

### Evaluation of *Ryt*RedAm Cofactor Specificity

**Figure 8:**
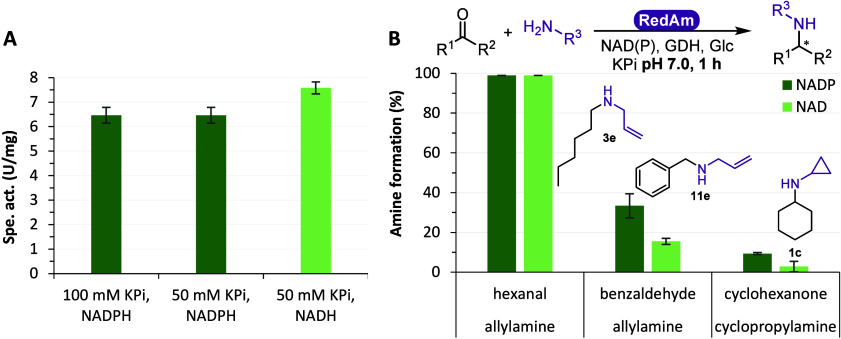
Cofactor specificity. (A) Specific activity with NADPH and NADH. Conditions: 100 or 50 mM KPi pH 7.0, 10 mM hexanal, 100 mM allylamine, 1% v/v DMSO, 0.2 mM NAD(P)H, *Ryt*RedAm, 25 °C. (B) 1 h conversions of carbonyl substrates with 10 equiv of amine donor with NADP^+^ (dark green) and NAD^+^ (light green). Conditions: 100 mM KPi pH 7.0, 10 mM carbonyl, 100 mM amine, 30 mM Glc, 12 U/mL *Bs*GDH E170K_Q252L, 0.2 mM NAD(P)^+^, 0.5 mg/mL *Ryt*RedAm, 500 rpm, 30 °C, 1 h.

*Ryt*RedAm specific activity was measured with each NADPH and NADH cofactor and was surprisingly slightly higher with NADH (7.6 U/mg, Figure 8A), which has been so far unreported for RedAms and IREDs,^37,38^ and would significantly increase the economic and synthetic potential of these enzyme families, allowing for NAD-dependent biocatalytic processes. Conversions of 1 h were run with varying combinations of carbonyls and amines with each NADP^+^ or NAD^+^ with a GDH/Glc recycling system (Figure 8B). Full conversion was obtained for **3e** with both cofactors, whereas **11e** and **1c** with lower conversions showed the slight preference for NADPH over NADH, which may also be ascribed to the GDH employed for cofactor recycling (*Bacillus subtilis Bs*GDH E170K_Q252L, SI Section 1).^39^

At first glance, the cofactor binding motif in the *Ryt*RedAm sequence is similar to other IREDs/RedAms that have strong NADPH preference.^21,38^ However, when the kinetic parameters were measured (Table 2, SI Figures S12–S13), the catalytic efficiency *k*_cat_/*K*_M_ with NADH was only seven times lower than that with NADPH. Noticeably, the *k*_cat_ for NADH is 3-fold higher, which explained the higher specific activity observed earlier. Kinetic parameters of *Asp*RedAm with NADH are not known, but it is reported that *Asp*RedAm is approximately 150 times more active with NADPH than with NADH.^26^ When measuring the activity with cyclohexanone and methylamine, Aleku et al.^26^ reports a *K*_M_ of 120 μM for NADPH, which is ten times higher than that of the *Ryt*RedAm *K*_M_ of 15 μM. One IRED from *Myxococcus stipitatus* was engineered using the Cofactor Specificity Reversal Structural Analysis and Library Design (CSR-SALAD) to change cofactor specificity from NADPH to NADH, resulting in a V10 mutant with an overall *k*_cat_/*K*_M_ of 7.9 min^–1^mM^–1^ with a *K*_M_ of 11 mM.^37,38^ In our study, *Ryt*RedAm gave a catalytic efficiency of 2187 min^–1^mM^–1^ with NADH, with a *K*_M_ value of 0.25 mM (Table 2). One recent report from Ward and co-workers describes a range of IREDs accepting both NADH and NADPH as cofactors for reductive amination at pH 7 and 9, but no kinetic data is reported.^40^*Ryt*RedAm displays significant activity for NADH that is uncommon in RedAms and IREDs, including engineered variants. This enzyme model enables opportunities for protein engineering of other RedAms and IREDs and the further discovery of other “cofactor-flexible” enzymes.

**Table 2:** *Ryt*RedAm Kinetic Parameters *K*_M_ and *k*_cat_ for NADPH and NADH^a^

Cofactor	*K*_M_ (μM)	*k*_cat_ (s^–1^)	*k*_cat_/*K*_M_ (s^–1^mM^–1^)	*k*_cat_/*K*_M_ (min^–1^mM^–1^)
NADPH	15 ± 4	3.6 ± 0.2	241	14440
NADH	247 ± 24	9.0 ± 0.3	36	2187

a Conditions: 100 mM KPi pH 7.0, 10 mM hexanal, 100 mM allylamine, NADPH or NADH, *Ryt*RedAm, 30 °C (SI Section 3.2).

### Crystal Structure and Mechanism

**Figure 9:**
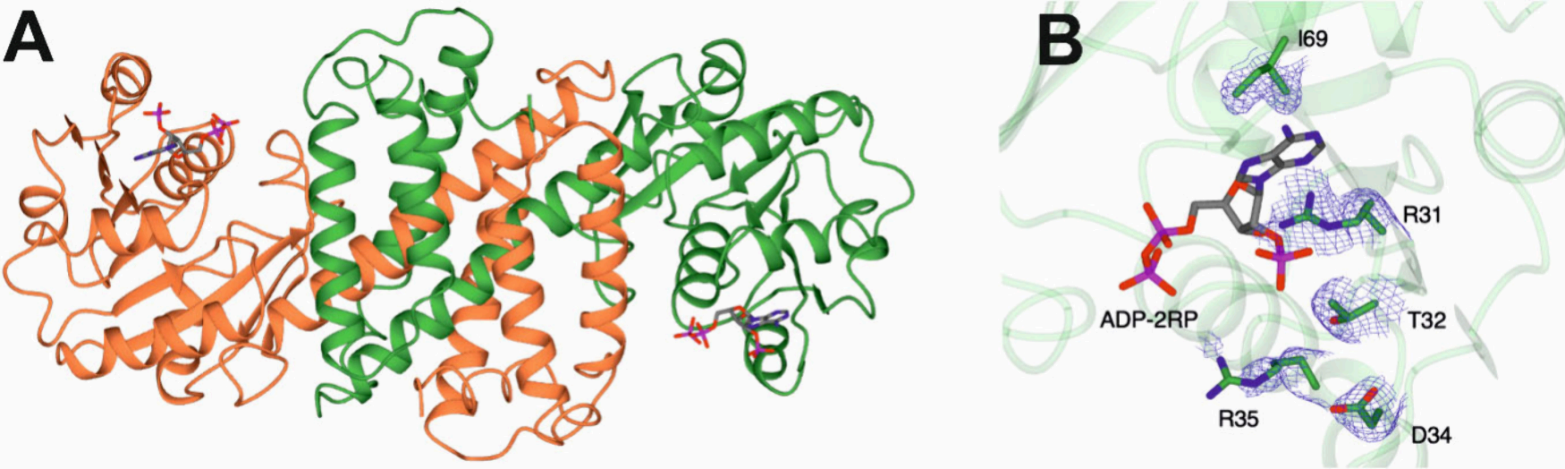
Crystal structure and active site residues of *Ryt*RedAm. (A) Structure of dimer of *Ryt*RedAm (PDB entry 9FM7) with subunits in coral and green. (B) Cofactor binding site of *Ryt*RedAm showing selected side chains and ADP-2RP with carbon atoms in green and gray, respectively. The electron density map shown is the 2*F*o-*F*c map at a level of 0.6 σ clipped to the side chains to show R35 pointing away from the cofactor binding site.

In an effort to shed light on the determinants of cofactor binding by *Ryt*RedAm, its structure was determined by X-ray crystallography in two forms. Each structure was obtained in space group *P*3_2_21 with one molecule in the asymmetric unit; therefore, the IRED dimer was constructed using the symmetry neighbor. The first, an apo-structure, was determined in the absence of cofactor (PDB 9FM8). While cocrystallization with NAD^+^ or NADP^+^ failed to give a structure with a fully intact cofactor, crystals obtained with the latter gave an ADP-2′-ribose phosphate (ADP-2RP) complex (density for the nicotinamide ring and ribose sugar of NADP^+^ being absent) that revealed the residues involved in cofactor binding. A search of the structural databases with the *Ryt*RedAm monomer using DALI^41^ revealed fairly low sequence homology with determined structures, including IREDs from *Streptomyces albidoflavus* (PDB 7XE8,^42^ 33% sequence id; *Z*-score 32.8; rmsd 1.3 Å over 284 Ca atoms) and *Mycobacterium smegmatis* (6SMT;^43^ 30%; 32.7; 2.6 Å over 283 Ca atoms). Neither of these enzymes was reported to use NADH as the cofactor in addition to NADPH. The structure of the reconstituted dimer of the *Ryt*RedAm ADP-2RP complex is shown in Figure 9A (PDB entry 9FM7), and the residues involved in cofactor binding are shown in Figure 9B.

**Figure 10:**
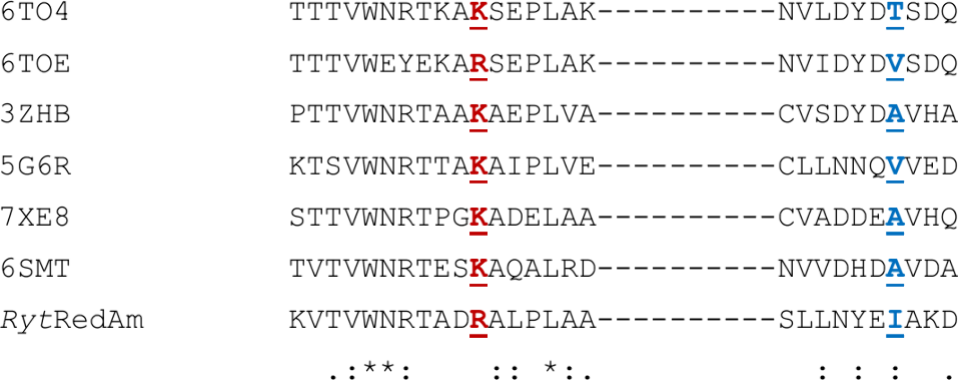
Sequence alignment of selected IREDs in cofactor binding regions. 3ZHB and 5G6R are NADPH-specific IREDs. 6TO4 is the NADPH-specific IRED from *Myxococcus stipitatus* that was engineered into 6TOE, a variant with improved NADH specificity through, among others, mutation of K37 (red) to R and T71 to V (blue). Natural *Ryt*RedAm is seen to have R35 and hydrophobic I69 at these positions.

A consideration of the residues involved in cofactor binding is revealing when compared with natural IREDs with NADPH specificity and especially those that have been engineered previously for NADH recognition. These include the enzyme from *Myxococcus stipitatus*,^38^ for which crystal structures of both the NADPH-dependent wild-type (6TO4) and NADH-dependent mutant (6TOE) have both been presented by one of our groups.^44^ An alignment of selected cofactor binding regions for a range of IREDs is shown in Figure 10.

The alignment reveals that, although many residues are involved in cofactor binding, two of the most effective mutations for increasing NADH binding in 6TO4 were at positions K37 and T71 to R and V, respectively. K37 is also present in enzymes of established NADPH specificity including 3ZHB, *Asp*RedAm 5G6R, and also the closest extant structural homologues of *Ryt*RedAm, 7XE8 and 6SMT. *Ryt*RedAm already has an R in position 35 equiv to 37; in the structure of *Ryt*RedAm in complex with ADP-2RP, the side chain had incomplete density for the terminal guanidinium group, but the map at a level of 0.6 σ indicates that the side chain is pointing away from the phosphate binding site. Ward, Hailes, and co-workers^40^ suggest that this K to R substitution does not always have to be present for IREDs to display at least some NADH-dependent activity. In 6TO4 position T71, *Ryt*RedAm has hydrophobic I69, which stacks against the adenine ring of ADP-2RP in the structure and is closer in chemical character to the valine in the NADH-dependent 6TOE variant. A consideration of the variant data suggests that the preference for either cofactor is a complicated phenomenon with synergistic input from a number of residues, but the observations for *Ryt*RedAm may provide useful indicators for the mutations of other IREDs to alter cofactor specificity.

## Conclusions

We discovered bacterial reductive aminases via EnzymeMiner and HMM approaches; in particular, *Ryt*RedAm represents another family of RedAms that catalyze reductive amination at neutral pH and accepts both NADPH and NADH cofactors. It is active on a panel of carbonyl substrates, especially aldehydes, to produce secondary amines and provides a chiral γ-lactam with 95% *ee* and (*S*)-*N*-methyl-1-phenylethylamine, with selectivity opposite to that of fungal RedAms. The higher activity at pH 7 compared to pH 9 shows this enzyme catalyzes the formation of the iminium ion.

*Ryt*RedAm displays an interesting profile regarding substrate preference, enantioselectivity, cofactor specificity, and reaction conditions. The crystal structure obtained provides insights for its NADH acceptance. Because of its complementarity with previously characterized RedAms and IREDs, *Ryt*RedAm is a good model to investigate the reductive amination reaction mechanism with varying substrate combinations with NADH as a cofactor for further scale-up applications.

*Ryt*RedAm could benefit from protein engineering to obtain other chiral amine products and higher thermostability. Other optimizing strategies such as reaction temperature, amine donor equivalents, and protein concentrations can be investigated. *Ryt*RedAm is a promising example of the potential undiscovered pool of bacterial reductive aminases, and further discovery of enzymes from this family is ongoing.

## Supporting Information Available

Details on the genes and production of enzymes, protocols for the synthesis of amines, enzymatic activity assays, biotransformations, analytical measurements, crystallization, GC chromatograms, and NMR spectra (PDF)
