# Supplementary material for: Discovery and Synthetic Applications of a NAD(P)H-Dependent Reductive Aminase from Rhodococcus erythropolis
Source: ACS Catal. 2024 Dec 16;15(1):211–9. doi: 10.1021/acscatal.4c04935 (PMC11705230; doi:10.1021/acscatal.4c04935)
Supplement: Supplementary file 1 — cs4c04935_si_001.pdf [file cs4c04935_si_001.pdf]

Supporting information

# Discovery and synthetic applications of a NAD(P)H-dependent reductive aminase from *Rhodococcus erythropolis*

Ewald P.J. Jongkind,[a] Jack Domenech,[b] Arthur Govers,[a] Marcel van den Broek,[a] Jean-Marc Daran,[a] Gideon Grogan,[b] Caroline E. Paul[a,*]

[a] *Department of Biotechnology, Delft University of Technology, van der Maasweg 9, 2629 HZ Delft, The Netherlands, c.e.paul@tudelft.nl*

[b] *York Structural Biology Laboratory, Department of Chemistry, University of York, Heslington, York YO10 5DD, UK*

## Contents

General information ........ 2
1. Gene sequences, enzyme production and purification, *Ryt*RedAm crystallization ........ 2
1.1. Accession numbers and sequences ........ 3
1.2. Enzyme production ........ 6
1.3. Affinity chromatography purification of *Asp*RedAm and *Ryt*RedAm ........ 8
1.4. Size exclusion chromatography purification of *Ryt*RedAm ........ 9
1.5. Expression and purification of *Ryt*RedAm for X-ray crystallography ........ 10
1.6. Crystallization, data collection and structure solution and refinement ........ 10
2. Carbonyl substrates, amine donors and products. ........ 12
2.1. Substrates and amine donors ........ 12
2.2. Amine products synthesis ........ 12
2.3. *N*-methylation of (*S*)-α-methylbenzylamine ........ 13
3. RedAm activity assays ........ 14
3.1. CFE activity of RedAm hits ........ 14
3.2. Steady-state kinetics of *Ryt*RedAm with NADH and NADPH ........ 15
4. RedAm reactions ........ 16
4.1. CFE reactions of RedAm hits ........ 16
4.2. *Ryt*RedAm 1-hour biotransformation ........ 16
4.3. *Ryt*RedAm reaction screening ........ 16
4.4. *Ryt*RedAm imine reduction screening ........ 16
4.5. Reductive amination with cyclohexanone and different amine donor concentrations ........ 17
5. GC analyses ........ 17
5.1. GC columns and methods ........ 17
5.2. GC chromatograms ........ 19
5.2.1. Amine products from cyclohexanone **1** ........ 19
5.2.2. Amine products from hexanal **3** ........ 22
5.2.3. Amine products from hydrocinnamaldehyde **4** ........ 24
5.2.4. Amine products from acetophenone **7** ........ 27
5.2.5. Amine products from ethyl levulinate **10** ........ 29
5.2.6. Amine products from benzaldehyde **11** ........ 30
5.2.7. Imine reduction by *Ryt*RedAm ........ 33
5.3. GC-MS column and chromatograms ........ 34
5.3.1. *N*-methylhexylamine **3b** ........ 34
5.3.2. *N*-hexylcyclopropanamine **3c** ........ 35
5.3.3. *N*-allylhexan-1-amine **3e** ........ 35
5.3.4. *N*-methylbenzylamine **11b** ........ 36
5.3.5. *N*-benzylcyclopropanamine **11c** ........ 36
5.3.6. *N*-benzylpropargylamine **11d** ........ 37
6. *Ryt*RedAm scale-up reactions ........ 38
6.1. Biotransformation to *N*-allylhexan-1-amine 3e ........ 38
6.2. Biotransformation to 1-cyclopropyl-5-methyl-2-pyrrolidinone 10c ........ 39
7. References ........ 40

## General information

Chemicals (aldehydes, amines, ketones) were purchased from abcr GmbH (Karlsruhe, Germany), Merck Sigma (St. Louis, Missouri, U.S.), TCI Europe (Zwijndrecht, Belgium), Thermo Fisher Scientific (Waltham, Massachusetts, U.S.) and VWR International BV (Amsterdam, the Netherlands) and were used as received without further purification. Amines were stored under nitrogen atmosphere. Aldehydes were newly bought before use and were checked by GC for purity. NADPH was bought from OYC EU B.V. (Rotterdam, The Netherlands) with a reported purity of ≥95%. NADH was purchased from Prozomix (Haltwhistle, United Kingdom).

Conversions and enantiomeric excess were measured on Shimadzu GC-2010 gas chromatographs (Kyoto, Japan) with an AOC-20i Auto injector equipped with a flame ionization detector (FID), using nitrogen or helium as the carrier gas. Products were confirmed by reference standards. Product concentrations were obtained with a calibration curve equation using 5 mM dodecane as an internal standard. All samples were injected with GC quality ethyl acetate (EtOAc), except where specified with diethyl ether ($Et_2O$) or methyl *tert*-butyl ether (MTBE).

Thin layer chromatography (TLC) were run on TLC polyester sheets with a silica gel layer POLYGRAM SIL G (Macherey-Nagel, Düren, Germany) and compounds were revealed using a potassium permanganate solution.

Nuclear magnetic resonance (NMR) spectra were recorded on an Agilent 400 spectrometer at 400 ($^{1}H$) and 100 ($^{13}C$) MHz. Chemical shifts (δ) are reported in parts per million (ppm) relative to $Me_4Si$ (δ 0.00) in deuterated chloroform ($CDCl_3$). NMR data is reported as follows: br = broad, s = singlet, d = doublet, t = triplet, m = multiplet, coupling constant(s) (*J*) in Hz, integration.

Specific rotation measurements were performed on a Perkin Elmer Model 343 S Polarimeter at 20 °C, at a wavelength of 589 nm with the solvent and concentration stated.

## 1. Gene sequences, enzyme production and purification, *Ryt*RedAm crystallization

EnzymeMiner search: The sequence from *Asp*RedAm (Q2TW47, PDB 5G6R) was used to identify targets on the online EnzymeMiner database. Two approaches were used: protein sequences that contained the six key residues N93, D169 Y177, W210, M239 and Q240, or contained only the first three of these residues. Then, the query identity range was set to 0-50%, as all hits with higher similarity come from fungal organisms. The predicted transmembrane protein sequences were excluded. The first hit with an identified organism strain was selected from *Streptomyces hygroscopicus*. The solubility factor was set to be a minimum of 0.5, determined by SoluProt. Then, the bacterial strains were manually selected. From eight sequences, *Streptomyces* and *Bacillus* hits were selected. From the remaining six sequences, one hit was selected from each family, since there were three pairs of sequences from the same family of *Amycolatopsis*, *Paenibacillus* and *Kibdelosporangium*. The sequence from *Rhodococcus rhodnii* was rationally selected to use a broad range of bacterial strains for this work (**Table S1**).

**Table S1.** Amino acids on key-residue positions of selected hits from bacterial organisms, found by EnzymeMiner. Sequence identity is based on the *Asp*RedAm sequence. Solubility is calculated by SoluProt.

| Organism | RedAm | 93 | 169 | 177 | 210 | 239 | 240 | Id. | Solub. |
|---|---|---|---|---|---|---|---|---|---|
| *Aspergillus oryzae* | *Asp*RedAm | N | D | Y | W | M | Q | 100 | 0.76 |
| *Amycolatopsis lurida* | *Alu*RedAm | N | D | Y | W | M | Q | 41.3 | 0.54 |
| *Bacillus* sp. J13 | *Bac*RedAm | N | D | Y | Y | M | Q | 39.5 | 0.66 |
| *Kibdelosporangium aridum* | *Kar*RedAm | N | D | Y | W | M | Q | 36.7 | 0.55 |
| *Paenibacillus ihbetae* | *Pih*RedAm | N | D | Y | Y | M | Q | 40.1 | 0.56 |
| *Rhodococcus rhodnii* LMG 5362 | *Rhod*RedAm | N | D | Y | W | L | N | 35.3 | 0.23 |

| | | | | | | | | | |
|---|---|---|---|---|---|---|---|---|---|
| *Streptomyces hygroscopicus* | *Shy*RedAm | N | D | Y | W | M | Q | 48.1 | 0.23 |
| *Streptomyces* sp. WAC 01529 | *Strep*RedAm | N | D | Y | W | I | N | 42.9 | 0.53 |

## 1.1. Accession numbers and sequences

All gene sequences were cloned in a pET-28a(+) vector with a *N*-terminal His-tag, without codon optimization.

***Aspergillus oryzae Asp*RedAm**, GenBank accession number: KY327363.1; UniProt number: Q2TW47
ATGTCCAAGCACATCGGTATCTTCGGTCTGGGTGCAATGGGTACCGCACTGGCTGCGAAATACCTGGAGCATGGTTACAAAACCTCTGTT
TGGAACCGTACTACCGCGAAAGCGATCCCGCTGGTTGAGCAGGGTGCTAAGCTGGCGTCTACCATCAGCGAAGGTGTTAACGCGAACG
ACCTGATCATTATCTGCCTGCTGAACAACCAGGTTGTTGAAGATGCGCTGCGTGACGCGCTGCAAACCCTGCCGTCTAAAACCATCGTTA
ACCTGACTAACGGTACTCCGAACCAGGCGCGTAAACTGGCAGACTTCGTTACCTCTCACGGTGCACGTTACATCCACGGTGGTATCATGG
CGGTGCCGACCATGATTGGCTCTCCGCACGCAGTGCTGCTGTACTCTGGTGAATCCCTGGAACTGTTTCAGTCTATTGAATCTCACCTGTC
TCTGCTGGGTATGAGCAAGTATCTGGGCACTGACGCGGGCTCTGCGAGCCTGCATGATCTGGCACTGCTGTCTGGCATGTACGGTCTGTT
CTCTGGTTTCCTGCACGCGGTGGCTCTGATTAAATCTGGTCAGGACACCTCCACCACTGCAACTGGTCTGCTGCCGCTGCTGACTCCGTGG
CTGAGCGCAATGACCGGTTACCTGAGCTCTATCGCGAAACAGATCGACGACGGTGATTACGCGACCCAGGGTTCTAACCTGGGCATGCA
GCTGGCTGGTGTTGAAAACATCATCCGTGCGGGTGAAGAACAGCGTGTTTCTTCTCAGATGATCCTGCCGATCAAAGCACTGATTGAACA
GGCGGTTGGTGAAGGTCATGGTGGCGAAGACCTGTCCGCGCTGATCGAATACTTCAAGGTGGGTAAAAACGTTGACTAA

>*Asp*RedAm protein sequence
MSKHIGIFGLGAMGTALAAKYLEHGYKTSVWNRTTAKAIPLVEQGAKLASTISEGVNANDLIIICLLNNQVVEDALRDALQTLPSKTIVNLTNGT
PNQARKLADFVTSHGARYIHGGIMAVPTMIGSPHAVLLYSGESLELFQSIESHLSLLGMSKYLGTDAGSASLHDLALLSGMYGLFSGFLHAVALI
KSGQDTSTTATGLLPLLTPWLSAMTGYLSSIAKQIDDGDYATQGSNLGMQLAGVENIIRAGEEQRVSSQMILPIKALIEQAVGEGHGGEDLSALI
EYFKVGKNVD

***Amycolatopsis lurida Alu*RedAm**, GenBank accession number: WP_091600158.1
ATGGGCAAGGCGCTGGCCGCCGCCTTCCTGGCCGCAGGCCACCCCACCACCGTCTGGAACCGCTCGGCGGGCAAAGCCGACAGCCTCGT
CGCGGACGGCGCGATCCAGGCGGCCACGATCACCGAGGCGGTCACGGCGAGCCCGATCGTGGTCGTCTGTCTGCTGGACTACCCGGTC
CTGCACGAGATTCTCGAGCCGGTGGGCGACACCTTGGCCGGTCGAGCACTGGTGAACCTCACCAACGGCACTCCGGACCAGGCTCGCGA
GACCGCCGGCTGGGCGAGAGGGCGCGGCGCCGACTACCTCGACGGCGGCATCATGGCCGTGCCCGCGATGATCGGCCGGCCCGAAGC
CCGCGTGCTCTACAGCGGCTCACGGCCTGTCTTCGACCAGTACGAGGGGACGCTGAACCGGCTGGGCACCGCCAGGTACGTCGGCGCC
GACCACGGCTTGGCGTCGCTCTACGACCTGGCCCTGCTCAGCGCGATGTACGGCCAGTTCGCGGGGGCGAGCCACGCACTCGCCCTCGT
CCGCACGGAGAAGGCCGATCTCACGGAATTCGCCTCGTCCCTGCTGGCCCCCTGGCTGACCGCGACGACCGTCGCGTTACCCCTGCTCGC
CGAACAAAACGACACCGGCACGCAGGCGGGCGAGGAATCCGCTTCCCCACCGGACATGCAGGCGGTGGCCATCGCCAACATCGTCACG
GCCAGCAACGCCCAGAACGTCGACAAAGCGCTGCTCTCCCACCTGTTCGTCCCCCTGCGAGACCTCATCGGCCGGCCTGCCCACGACCGC
GACCTCGCCGGAATGGTCGACCTGATCAAGAAACCCCAGGAG

>*Alu*RedAm protein sequence
MGKALAAAFLAAGHPTTVWNRSAGKADSLVADGAIQAATITEAVTASPIVVVCLLDYPVLHEILEPVGDTLAGRALVNLTNGTPDQARETAG
WARGRGADYLDGGIMAVPAMIGRPEARVLYSGSRPVFDQYEGTLNRLGTARYVGADHGLASLYDLALLSAMYGQFAGASHALALVRTEKAD
LTEFASSLLAPWLTATTVALPLLAEQNDTGTQAGEESASPPDMQAVAIANIVTASNAQNVDKALLSHLFVPLRDLIGRPAHDRDLAGMVDLIKK
PQE

***Bacillus* sp. *Bac*RedAm**, GenBank accession number: WP_028404255.1
ATGGTTCGATATTTCTTTCGACAAGGGAAAGGGGAAATCAAAGTGAGTACCGATCAAAGCAAGAACGAAGCTCTCTCTCCAGTGACCAT
CATCGGTCTTGGCGAGATGGGGCAGGCATTAGCAAATGTATTTTTGCAAAACGGATATCCTACGACGGTTTGGAACCGGACGGCAGCGA
AGGCAGAAGCCTTGGTGAAGCAGGGAGCTGTGCTTGCTGCCACACCTCGTGAAGCCATACAGGCAAGCCCGGTCGTGATCCTGTGCGTT
CTGGATTACGATGCCGTACATGAAATCCTTGATCCACTCGGTGATGCACTTAAGGGTCGTGTTCTGTTCAACCTAACGAACGGAACTCCTA
AGCAGGCAGGTGATACCGCACAATGGGCTAAGGATCGAGGTTACGATTATATCGATGCCGGTATCATGGCCGTTCCGCAAATCATCGGA
ACCGAGGATGCTTTTATCCTCTACAGTGGAGGAAACAAGAACTCCTTCGATTCCAATAAGGAATTGTTGGATGTAATGGGCGCGTCCACC
TACTTAGGCGAAGATGCCGGTTTAGCATCCTTGCTGGATCTAGCGATGAATGGTGCGATGTATGGGATGCTCGCCGGTGCCATGCACGC
TATCTCGGTAGTTGCCACAGAAGGGATCAAGGCACAGGCTTTCTCGTCAGAACTCTTGATTCCCTACCTCACTGCAATTACCGGCATCATA
CCTAATCTAGCCCGCCAGTTCGACACGAAAGAATTTACGGTTGGCGTCTCGGCCAAACTTGCCATGCAGCAGGTGGGTTTCAGAAATATT
CGGCAAGCCAGCAAGGACCAAGGGATTAGTACAGAGCTGCTCGACCCGATCCAATCTCTTATGGACCGGCGTGTCGCTGCCGGGTTCCC
CGATGACGACTTCTCCGCTGTTACCGAGCTTTTTAAACAAGCAAAGCACCAAAACACGTGA

>*Bac*RedAm protein sequence
MVRYFFRQGKGEIKVSTDQSKNEALSPVTIIGLGEMGQALANVFLQNGYPTTVWNRTAAKAEALVKQGAVLAATPREAIQASPVVILCVLDYD
AVHEILDPLGDALKGRVLFNLTNGTPKQAGDTAQWAKDRGYDYIDAGIMAVPQIIGTEDAFILYSGGNKNSFDSNKELLDVMGASTYLGEDAG
LASLLDLAMNGAMYGMLAGAMHAISVVATEGIKAQAFSSELLIPYLTAITGIIPNLARQFDTKEFTVGVSAKLAMQQVGFRNIRQASKDQGIST
ELLDPIQSLMDRRVAAGFPDDDFSAVTELFKQAKHQNT

***Kibdelosporangium aridum Kar*RedAm**, GenBank accession number: WP_051793401.1
ATGGGCGAGACGTTGGCCGAGACGTTCGTAAGCAACGGTCACCCGACGACCGTGTGGAATCGCACGCCAGGAAAGGTCGTCGAGGGC
GCGACCCATGCTCCGACCGCCGCGGAAGCGGTCGCTGCCAGCGAGGTCGTTGTCGTGTGCGTGCTCGACTACAAGGCCGCGCGTGAGG
TTCTGGATCCCATCGACCTGACCGGCAAGGCCGTGGTCAACCTGACCAACGGCACGCCCGCGCACGCCAGGGAATTCGTCCGAGGTGAC
TATCTGGACGGCGGGATCATGGCTGTCCCGCAGATGATCGGCACACCTGAGGCGATCGTGCTCTACAGCGGGTCGCGCACGGTCTTTGA
CAATTATCAGGACACGTTGAACGTGCTGGGGCAGAGCAGGTTCGTGGGAGAAGATCCGGGACTCGCCGCGCTGTATGACCTGGCACTG
CTCAGCGCGATGTACGGCCAGTTCGCCGGAGCGAAGCACGCGCTGGACATGGTCGGCGCGAACAGGGGCGACTTCGTGGAAACCCTGC
TGATCCCGTGGCTGACGTCGACCATGGTCGCGATCCCGGTGCTGGGCCAGAGCGATCCGCAATCCCCGGACGACATGCAAGCCGTGGC
GATCGGCAACATCATCGAGGCCAACGAGGACCTTGGTCTGGGACAGCCGGGACACCTGCGA

>*Kar*RedAm protein sequence
MGETLAETFVSNGHPTTVWNRTPGKVVEGATHAPTAAEAVAASEVVVVCVLDYKAAREVLDPIDLTGKAVVNLTNGTPAHAREFVRGDYLD
GGIMAVPQMIGTPEAIVLYSGSRTVFDNYQDTLNVLGQSRFVGEDPGLAALYDLALLSAMYGQFAGAKHALDMVGANRGDFVETLLIPWLTS
TMVAIPVLGQSDPQSPDDMQAVAIGNIIEANEDLGLGQPGHLR

***Paenibacillus ihbetae Pih*RedAm**, GenBank accession number: WP_099476365.1
ATGAATACCGATCAAAGCAAGAGAAATGCCCTCTCTCCAGTGACCATCATCGGTCTTGGCGAGATGGGGCAGGCATTAGCAAATGTATT
TTTGCAAAACGGTTATCCTACGACGGTTTGGAACCGGACGGCAGCGAAAGCAGAAGCTTTGGTGAAGCAAGGAGCTGTGCTTGCTGCCA
CACCTTATGAAGCCATACAGGCTAGTCCAGTCGTGATCCTGTGCGTTCTGGATTACGATGCCGTACATGAAATCCTTGATCCGCTCGGTG
ATGCACTTAAGGGTCGTGTGCTGTTCAACCTAACGAACGGAACTCCAAAGCAGGCACGTGATACCGCCCAGTGGGCTAAGGATCTAGGT
TACGATTATATGGATGCCGGTATCATGGCCGTTCCGCAAATCATCGGAACCGAGGCTGCTTTCATTCTCTACAGTGGAGGAAACAAGGAA
TCCTTCAATTCCAATAAGGAATTGTTGGATGTCATGGGCGCGTCCACCTACTTAGGCGAAGATGCCGGTTTAGCATCCTTGCTGGATCTA
GCGATGAATGGAGCGATGTATGGGATGCTCGCCGGTGCCATGCACGCTATCTCGGTAGTTGCCACAGAAAGGATCAAGGCACAGGATT
TCTCGTCAGAACTCTTGATTCCCTACCTCACTGCAATTACCGGCATCATACCTAATCTAGCCCGCCAGTTTGACACGAAAGAATTTACGGTT
GGCGTGTCGGCCAAACTTGCCATGCAGCAGGTTGGTTTCAGAAATATCCGGCAGGCCAGCAAGGACCAAGGGATTAGCACCGAGCTGC
TCGATCCCATCCAATCCCTAATGGATCGACGTGTCGCTGCCGGGTTCCCCGATGACGACTTCTCGGCCGTTACCGAGCTATTTAAAACACC
TAAGCAATAA

>*Pih*RedAm protein sequence
MNTDQSKRNALSPVTIIGLGEMGQALANVFLQNGYPTTVWNRTAAKAEALVKQGAVLAATPYEAIQASPVVILCVLDYDAVHEILDPLGDALK
GRVLFNLTNGTPKQARDTAQWAKDLGYDYMDAGIMAVPQIIGTEAAFILYSGGNKESFNSNKELLDVMGASTYLGEDAGLASLLDLAMNGA
MYGMLAGAMHAISVVATERIKAQDFSSELLIPYLTAITGIIPNLARQFDTKEFTVGVSAKLAMQQVGFRNIRQASKDQGISTELLDPIQSLMDRR
VAAGFPDDDFSAVTELFKTPKQ

***Rhodococcus rhodnii Rhod*RedAm**, GenBank accession number: EOM76188.1
ATGGGCACGCCGATAGCCGCCGCCTTCATCGACGCCGGATACCGAACGATCGTCTGGAATCGCAGCCCGGGAAAGGCCGATGCGCTGG
CCTCGCGAGGGGCCGAGCCCGCGGCCACCGCCGCCGAGGCGGTCGCCGCCGCGCCGCTGGTGGTCGCCCCGTTGCTCGACCACGTCGC
CGTACGTCAAACCTTGGTCCCGGCCACAGCCGCCCTACAGGGTCGTACGGTGGTCAACCTCGCCAATAGCACACCGGACCAGGCGCGCG
ACCTCGCCGCTTGGGTGGCCGGGCACGGTGCCGCGTATCTGGACGGGGCCATGATGGCGCTGCCGGACAGCGTCGCCACGCGAGAGG
GCTTCTTCCTTTACAGCGGTTCGGAGGAAGCGTTCACGCGCTACCGCAGTGCGTTGGAAGTCATGGCGCCCGCACACTACTTCGGCGCCG
ATCCGGGCGGGGCGGAGATCCACGACCTCGCGGTGCTGGGCACCGGATACGGTGCGCTTTCAGGCTTCCTGCACTCGCTTGCGATCCTG
CACGGGACGGGCACGAGCCCCGAGCATTTCGCCGCGCTGGCCGCGCGCTGGCTGAACGGCCTGGCCGCCTTCCTTCCGGAGCTGGCCC
GCGAAATCGACGCCGCGCACTATACGGACGGGATCTCGACCATCGATCTCAACCGTGCCGCCGTCGACGGGATCGTCGAGCTCGGCCGG
GCGAGCGGGGTATCCGCGGCCACCCACGAGCCCCTGCGCGACCTGTTGCATCGGAGCTCGGACAACGGGCGCGGCAAGGACAGTTTCT
CCAGCGTTTTCGAGCTGATGCGGAAGCGGGACGATCCCCGGCAA

>*Rhod*RedAm protein sequence
MGTPIAAAFIDAGYRTIVWNRSPGKADALASRGAEPAATAAEAVAAAPLVVAPLLDHVAVRQTLVPATAALQGRTVVNLANSTPDQARDLAA
WVAGHGAAYLDGAMMALPDSVATREGFFLYSGSEEAFTRYRSALEVMAPAHYFGADPGGAEIHDLAVLGTGYGALSGFLHSLAILHGTGTSP
EHFAALAARWLNGLAAFLPELAREIDAAHYTDGISTIDLNRAAVDGIVELGRASGVSAATHEPLRDLLHRSSDNGRGKDSFSSVFELMRKRDDP
RQ

***Streptomyces hygroscopicus Shy*RedAm**, GenBank accession number: WP_066027906
ATGAGCAAGACCCCCGTAACCGTCCTCGGACTCGGCGACATGGGCACCGCGCTGGCCCGCGCCCTGCTGGAGGGCGGACACCCGACGA
CGGTGTGGAACCGTACGGCCGCCAAGGCCGAGGCGCTCGCCCCCGAGGGCGCGCTGACCGCGGCCACGACCGGCGAGGCCGTCGCGG
CGAGCCGTCTGGTGGTGGTCTGCCTGCTGGACTACGACTCCGTCCGCCAGGTGCTGGGCCCCCTCGGGGAGGCCCTGGCCGGACGGAC
CGTCGTCAACCTCACCAACGGCACTCCACGGCAGGCCCGCGACCTCGCCGCATGGGCGGCCGGACACGGCGCCGAGTACATCGACGGC
GGCATCATGGCCGTCCCGCCGATGATCGGGACACCCGCCGCCTTCCTCCTCTACAGCGGCTCACCCGCCGCCTTCGCCGCCCACCGGTCC
GTGCTGGACCTCTTCGGCGAGAGCCACCACCTCGGCGAGGACCACGGTCGTGCCCCGCTGTACGACCTCGCTCTGCTCAGCGCGATGTA
CGGGATGTTCTCGGGCGTGCTGCACGCCTACGCCCTGGTGCGGTCGGACGGCGTCGCGGCGGGGGACGTCGCCCCGCTGCTGGGGCGC
TGGCTGACCGCCATGTCCGGGGCCGTGGACGGTTACGCCCAGCGGATCGACTCCGGCGACCACGCCACCGGCGTGGTCTCCACCATCGC

CATGCAGTCCGCCGCGTTCGGCAACTTCACCGGCTCCGCGCGGGACCAGGGCATCAGCCCCGAACTGATCGCCCCGATCGGCGCGCTCA
TGGCCCGCCGGGTGGCCGCTGGCCACGGCCATGAGGACCTCACGGGGCTGGTGGAACTGCTCACCGCGTAA

>*Shy*RedAm protein sequence
MSKTPVTVLGLGDMGTALARALLEGGHPTTVWNRTAAKAEALAPEGALTAATTGEAVAASRLVVVCLLDYDSVRQVLGPLGEALAGRTVVNL
TNGTPRQARDLAAWAAGHGAEYIDGGIMAVPPMIGTPAAFLLYSGSPAAFAAHRSVLDLFGESHHLGEDHGRAPLYDLALLSAMYGMFSGV
LHAYALVRSDGVAAGDVAPLLGRWLTAMSGAVDGYAQRIDSGDHATGVVSTIAMQSAAFGNFTGSARDQGISPELIAPIGALMARRVAAGH
GHEDLTGLVELLTA

***Streptomyces* sp. *Strep*RedAm**, GenBank accession number: WP_125517916.1
ATGGGCAGCGCTCTCGCCGCCGCCCTGCTCAGGGCGGGCCACCGGACGACCGTCTGGAACCGCACCGCCGCCAAGACCGGCCCCCTGG
CCGCCCAGGGCGCGACCCCCGCCGAGACGGCCGCGGAGGCCATCGAGGCGAGCGCCCTCGTCATCGTCTGCCTGACGACCAACGACAA
CGTCCGCACCCTCCTGGAGCCCGAGGCCGCCGCCCTCGCCGGCCGCACCGTCGTGAACCTCACCAACGGAACCCCGGCACAGGCACGGG
AGTTGGCGCACTGGGCGGCCGAGCACGGCATCACGTACATCGACGGCGGCATCATGGCCGTACCGCAGATGATCGCCACACCCGGCGC
GTACATCCTGTACAGCGGCACCGACGAAGAGGCGTACGAGACGCACCGGCCCACGCTGGCGGCGCTCGCGGAGACCAAGTGGGTCGG
CAAGGACCCGGGCGCCGCCGCGCTGTACGACCTGTCCCTGCTCACCGGCATGTACGGCATGGTGATGGGCGTCGCCCAGGCCTACGCCC
TGATCGGCACCGGAGGGGTCCCGGCCCGTGACTTCGCGCCCCTGCTCAAGGAGTGGGTCAACGCGATGACGGACGGCCTGGTGCCCGG
CATGGCCGAGGCGCTGGACTCCGGGCAGCACCTCACGGACGTGTCGTCCCTGGCGATCAACCAGGCCGCGCTCCCCAACTTCCTGGACG
CCTTCGCCCAGCAGGGCCTGAGCGGCGCCCTGTTCGAGCCGCTCCAGGCCCTCCTGGACCGCTCGGTCGAGGAGGGATACGGCGCCGA
CGGCCTCTCCCGCCTCGCCACCCTGATCAAGAAGGAG

>*Strep*RedAm protein sequence
MGSALAAALLRAGHRTTVWNRTAAKTGPLAAQGATPAETAAEAIEASALVIVCLTTNDNVRTLLEPEAAALAGRTVVNLTNGTPAQARELAH
WAAEHGITYIDGGIMAVPQMIATPGAYILYSGTDEEAYETHRPTLAALAETKWVGKDPGAAALYDLSLLTGMYGMVMGVAQAYALIGTGGV
PARDFAPLLKEWVNAMTDGLVPGMAEALDSGQHLTDVSSLAINQAALPNFLDAFAQQGLSGALFEPLQALLDRSVEEGYGADGLSRLATLIKK
E

***Rhodococcus rhodochrous* reductive aminase *Roc*RedAm**, GenBank accession number WP_059384799
ATGGGCAGTGCCCTCGCCTCACGACTCCTCGACATCGGCTACCACGTGACGGTCTGGAATCGGAGCCCTGGTCGCGACACCGTTCTGGTT
GAGAGCGGCGCTCACCCCGCTGAGACCGTTGCCGCTGCGGCTGGGGCGAACCCACTCATCGTCGCCTGTCTGCTCCGGGCGACGTCGGT
TTACGAGACCCTCACCCCGGTGGTCGAGCAGTTGCGGGGGCGCACCTTGATCAATCTCACGACCACCACCCCGAACGAGGCTCGCGCGC
TCGCCGATTGGGCCGATCGGCACGGCATCGCCTACCTGACCGGCGCAATCCTGGCGGTCCCGGACATGATCGGCACTCCCGCAGCACAG
ATCTTCTACAGCGGCCCGCAACCCATTTACGAGCAGCACCACGAACTCCTCGACACATGGGCGACCAGCACGTACGACGGCGCGGACCC
GGGGATGGCATCGCTGGTCGACTTGGCGATGCTTTCGGGCATGTACCAGATGTTCGCGGGATTCTTCCACGGCGCTGCCATGGTGGGAT
CCGAAGGCATGACCGCCGAGGAATTCGCGCGCCGCGCAACCCCATTCCTCCGCGCAATGACCAGCGGTTTCAGAGAATATGCGGCAGTC
ATCGATGCCGGCGACTACACCGCTCCGGGGCAACAGAGCTTGGAGTTCTCCGATCTCGGTCACATTGTGAGCGCCAGCGAGGAACAGTG
CGTCGATCCGGCGACACTCGTTGCCCTGCAGGGACTTATCACTCGGGAGATTGCCGCTGGTCACGGCTCCGAAGGCTTCGCCCGAGTCTT
CGTGAGCATGCGGGCCGACACGGGCGACCGCACCGCCGAACGCATCGCATGA

>*Roc*RedAm protein sequence
MGSALASRLLDIGYHVTVWNRSPGRDTVLVESGAHPAETVAAAAGANPLIVACLLRATSVYETLTPVVEQLRGRTLINLTTTTPNEARALADWA
DRHGIAYLTGAILAVPDMIGTPAAQIFYSGPQPIYEQHHELLDTWATSTYDGADPGMASLVDLAMLSGMYQMFAGFFHGAAMVGSEGMTA
EEFARRATPFLRAMTSGFREYAAVIDAGDYTAPGQQSLEFSDLGHIVSASEEQCVDPATLVALQGLITREIAAGHGSEGFARVFVSMRADTGDR
TAERIA

***Rhodococcus opacus* reductive aminase *Rop*RedAm**, GenBank accession number: WP_064080687.1
ATGACCGGCACCGACGTCACCGTTCTCGGGCTCGGCGCCATGGGACAGGCTATCGCCGTCGCGCTGCTGCGGGCGGGTCGCACGGTCA
CCGTCTGGAACCGCACGCCCGAGAAAGCGGACACGGTGACGAAGTCCGGTGCGCACCCGGCCGGGTCGGTGTCCGCGGCGGTGCAGG
CGTCGCCGCTGGTGCTGGTCTGCGTGCTCGACGACGAGACGGTGCACGAGCTGATCGAGCCCGTCGCCGGTGACCTGCGTGGACGAAC
GCTCGTGAACCTGACGACGACCACGCCCGAGCAGGCGCGTGCGATGTCGCGGTGGGCCGGCGCGGCGCGAGTGGAGTACGTCGACGG
CGGCATCATGGCGGTGCCCGACATGATCGGGGGCGGCGACACGTTCGTGCTCTACAGCGGCGCAGCGGAGGCGTTCGACAGGAATCG
GCCGGTGCTGGAACTGTTCGGTCGTGCGGTATTCGTCGGCGACGACGCCGGCGCTGCGGCGATGTACGACCTCGCGCTGCTCGGCGCG
ATGTACGCCATGTTCGCCGGGTTCGAGCAGGGCGCCGCGATGGTCCGAGGCGCCGGCGGCACCGCGGGGGAACTCGCCGCAATGGCG
GCGCCGTTCCTTCAGGCGATGACCGGAAGCTTCGAGGAGTTCGCGGTCGGAATCGACACTCCCGCGCAGTACGAGCCGGTGCAGAGCG
CGGAATTCACCGCGGCGGCAATCGACACGGTCGCACGTGCGGGCGCCGAAGCGGGCGTGCCGACCGCTCTGCCGATCGCCGTGCGTGC
GGTTCTCGCGGGTTCTATCGACGCAGCATGTCCCTGA

>*Rop*RedAm protein sequence
MTGTDVTVLGLGAMGQAIAVALLRAGRTVTVWNRTPEKADTVTKSGAHPAGSVSAAVQASPLVLVCVLDDETVHELIEPVAGDLRGRTLVNL
TTTTPEQARAMSRWAGAARVEYVDGGIMAVPDMIGGGDTFVLYSGAAEAFDRNRPVLELFGRAVFVGDDAGAAAMYDLALLGAMYAMFA
GFEQGAAMVRGAGGTAGELAAMAAPFLQAMTGSFEEFAVGIDTPAQYEPVQSAEFTAAAIDTVARAGAEAGVPTALPIAVRAVLAGSIDAA
CP

***Rhodococcus erythropolis*** **reductive aminase *Ryt*RedAm**, GenBank accession number: WP_020971038.1
ATGGACGTATCAATACTCGGCACCGGCTTGATGGGAACAGCTCTCGCTCAGGCGCTCATACGCAGCGGGACAAAGGTGACGGTGTGGA
ATCGAACCGCCGACAGAGCGTTACCCCTCGCTGCGGCTGGAGCTACAGTCGCCGAATCTCCACAATCTGCGATTGCGGCCAGCCCGCTA
ATCGTCATCAGTCTCCTGAACTACGAGATCGCCAAAGATGTCGTTACAGAAGCAGATTCGATTGCCGGAAAAATCATCGTCAACACCGCA
ACCGGTACGCCGGAGGAAGCGAACCAGTTCGCCGAGTGGATCGCAGGGCGAGGCGCTCGGTACCTCGACGGAGCCATAGCTGCATACC
CGGAGGACATCGGAACCGAAAGTTCAGGGATCAACTATTCCGGAGACGAGGACGTGTGGGAGGACGTCCAGAGCCTTCTGACACCCAT
CGCCGCCCAATCACGATATGTCGGAGCCCGTCCCGGTGCCGCCAACGTGATCGACGCGGCCATGGCAGGCGCATTCTTCAACGTCGCGC
TCGGTGCCTTCCACGAAGCAGCAGCGTACGTGCGGTCCGAGGACGTCGCGATCGCGGAGATGCGCCACAGTTTGCACCTGTGGACCGA
CAAACTTCTCGAACTCCTTCACGAAGCACTCAAAGCGTTCGAATCCGGAGAGTACGAGACCGATCAGGCAACCTTGAACGTATACGCAGC
CGCAGTAGAAGCCTGGCAACAATCGATGCAGCGAGCAGGCCAACGCGCCGCTCTGATGACGGCGAATCTCGACAACTTGCAACGTGCA
TGCGCAGCCGGGCACGGGGACAAGGGCATCTTCGCCCAAATCGAAACACTCTCCGCCAACCCTCAATCCGCAATCTAA

>*Ryt*RedAm protein sequence
MDVSILGTGLMGTALAQALIRSGTKVTVWNRTADRALPLAAAGATVAESPQSAIAASPLIVISLLNYEIAKDVVTEADSIAGKIIVNTATGTPEEA
NQFAEWIAGRGARYLDGAIAAYPEDIGTESSGINYSGDEDVWEDVQSLLTPIAAQSRYVGARPGAANVIDAAMAGAFFNVALGAFHEAAAYV
RSEDVAIAEMRHSLHLWTDKLLELLHEALKAFESGEYETDQATLNVYAAAVEAWQQSMQRAGQRAALMTANLDNLQRACAAGHGDKGIFA
QIETLSANPQSAI

***Nocardia seriolae*** **reductive aminase *Noc*RedAm,** GenBank accession number: WP_033085900
ATGTCTGAACCGCGTCGATCCGTCACCGTCATCGGCCTGGGCCCGATGGGCCGCGCGATGGTCAAAGCGTTCCTGGCCGCCGGGGTCGA
GGTCACGGTGTGGAACCGCAGCCCCGAGAAGGCCGATGCCATGGCCGAGTCGGGTGCGAAGCGGGCCGGGACCGTGGCCGAGGCCCT
CGACGCCAACGAGGTGATCGTCGTGAGCCTCACCCACTACGCGGCCATGTACGACGTGCTCGAGCCGGTCGCGGATCGACTGCGCGGC
AAGGTGATCGCGAACCTGTCCTCCGATTCTCCGGAGAACGCCCGCAAGGGCGCGGCCTCGGTGCGATCGTTCGGCGCGCGATTCCTTTC
GGGCGGTTGCATGACCGTCTCCGACGATATCCTGCACCCCGCCTCGTACATCTTCTACAGTGGCCCACGCGAGGTCTTCGACGCGCACGC
CGAGCTGCTGCGCCCGCTCAGCCCGCAGGAGTACCTGGGCGCGGACGACGGTCTGTCCCAGGTCTACTACCAGGCCCTGCTCACCATCTT
CCACTCGTGGATGCTGGCCCTGGACCAGGCTTTCGCGCTGATCGTCAACTCCGGCAACGAGATCGGTCATTTCCTGCCCTACGCGCTGCG
CTCGCAGACCCCCTTCGCCGATTTCATGGCGAACTTCGCGGCCGCGGCCGAGGCGGGCGGCTGGGGCGACCTGGCCAACCTGCGCATG
ATGGACGCGGGTGCGCAGCATGTCATCGAGGCGAGCGAGGACGCGGGGGTCGATGCCTCGCTCGCGCACGCCGCGCAGGCCCTGTGG
CGCAAGGCCATTGCCGCCTCCGAAACCGCAGGGGCCCCGGTACCGGTCTTTCGGATACTGAAAGGCACTGCGGCCTAA

>*Noc*RedAm protein sequence
MSEPRRSVTVIGLGPMGRAMVKAFLAAGVEVTVWNRSPEKADAMAESGAKRAGTVAEALDANEVIVVSLTHYAAMYDVLEPVADRLRGKVI
ANLSSDSPENARKGAASVRSFGARFLSGGCMTVSDDILHPASYIFYSGPREVFDAHAELLRPLSPQEYLGADDGLSQVYYQALLTIFHSWMLAL
DQAFALIVNSGNEIGHFLPYALRSQTPFADFMANFAAAAEAGGWGDLANLRMMDAGAQHVIEASEDAGVDASLAHAAQALWRKAIAASET
AGAPVPVFRILKGTAA

***Bacillus subtilis*** **glucose dehydrogenase *Bs*GDH E170K_Q252L**, UniProt number: P12310
ATGTATCCGGATTTAAAAGGAAAAGTCGTCGCTATTACAGGAGCTGCTTCAGGGCTCGGAAAGGCGATGGCCATTCGCTTCGGCAAGGA
GCAGGCAAAAGTGGTTATCAACTATTATAGTAATAAACAAGATCCGAACGAGGTAAAAGAAGAGGTCATCAAGGCGGGCGGTGAAGCT
GTTGTCGTCCAAGGAGATGTCACGAAAGAGGAAGATGTAAAAAATATCGTGCAAACGGCAATTAAGGAGTTCGGCACACTCGATATTAT
GATTAATAATGCCGGTCTTGAAAATCCTGTGCCATCTCACGAAATGCCGCTCAAGGATTGGGATAAAGTCATCGGCACGAACTTAACGG
GTGCCTTTTTAGGAAGCCGTGAAGCGATTAAATATTTCGTAGAAAACGATATCAAGGGAAATGTCATTAACATGTCCAGTGTGCACGAA
GTGATTCCTTGGCCGTTATTTGTCCACTATGCGGCAAGTAAAGGCGGGATAAAGCTGATGACAAAGACATTAGCGTTGGAATACGCGCC
GAAGGGCATTCGCGTCAATAATATTGGGCCAGGTGCGATCAACACGCCAATCAATGCTGAAAAATTCGCTGACCCTAAACAGAAAGCTG
ATGTAGAAAGCATGATTCCAATGGGATATATCGGCGAACCGGAGGAGATCGCCGCAGTAGCAGCCTGGCTTGCTTCGAAGGAAGCCAG
CTACGTCACAGGCATCACGTTATTCGCGGACGGCGGTATGACACTCTATCCTTCATTCCAGGCAGGCCGCGGT

>*Bs*GDH E170K_Q252L protein sequence
MYPDLKGKVVAITGAASGLGKAMAIRFGKEQAKVVINYYSNKQDPNEVKEEVIKAGGEAVVVQGDVTKEEDVKNIVQTAIKEFGTLDIMINNA
GLENPVPSHEMPLKDWDKVIGTNLTGAFLGSREAIKYFVENDIKGNVINMSSVHEVIPWPLFVHYAASKGGIKLMTKTLALEYAPKGIRVNNIG
PGAINTPINAEKFADPKQKADVESMIPMGYIGEPEEIAAVAAWLASKEASYVTGITLFADGGMTLYPSFQAGRG

## 1.2. Enzyme production

Lyophilized plasmids of the selected sequences from EnzymeMiner and the *Rhodococcus* sequences cloned in pET-28a(+) were ordered and received from SynBio Technologies (Monmouth Junction, NJ, United States). The *Asp*RedAm plasmid was kindly provided by Prof. N.J. Turner (University of Manchester, Manchester Institute of Biotechnology, UK).

*E. coli* BL21(DE3) chemically competent cells were transformed with the vector containing the listed genes unless stated otherwise. The *Asp*RedAm gene was transformed in *E. coli* BL21(DE3), C43(DE3) or

BL21 Gold(DE3) competent cells. The transformed cells were grown on selective LB-agar plates (50 µg/mL kanamycin) overnight at 37 °C. Terrific Broth (TB)-medium (500 mL in 2 L baffled flask) was inoculated with 1% v/v overnight LB preculture of the transformed cells and incubated for 3-5 h at 37 °C, 180 rpm. After reaching an $OD_{600}$ of 0.6-0.8, 0.5 mM IPTG was added, followed by overnight incubation at 20 °C. Cells were harvested (17,000 × *g*, 20 min, 4 °C) and stored at -80 °C.

Sodium dodecyl sulfate polyacrylamide gel electrophoresis (SDS-PAGE) was run to show gene expression and enzyme purity levels (**Figures S1-S4**). Samples were prepared by mixing with one equivalent of Laemmli buffer and 5% v/v dithiothreitol (DTT), heated to 95 °C for 5 min, then centrifuged at 9,000 × *g* for 2 min. From these samples, 10 µL was loaded onto the gel, whereas 5 µL of protein ladder was loaded onto a Criterion TGX Stain-Free Precast Gel. Imaging was performed with a ChemiDoc MP imaging system (Bio-Rad Laboratories, Hercules, California, U.S.).

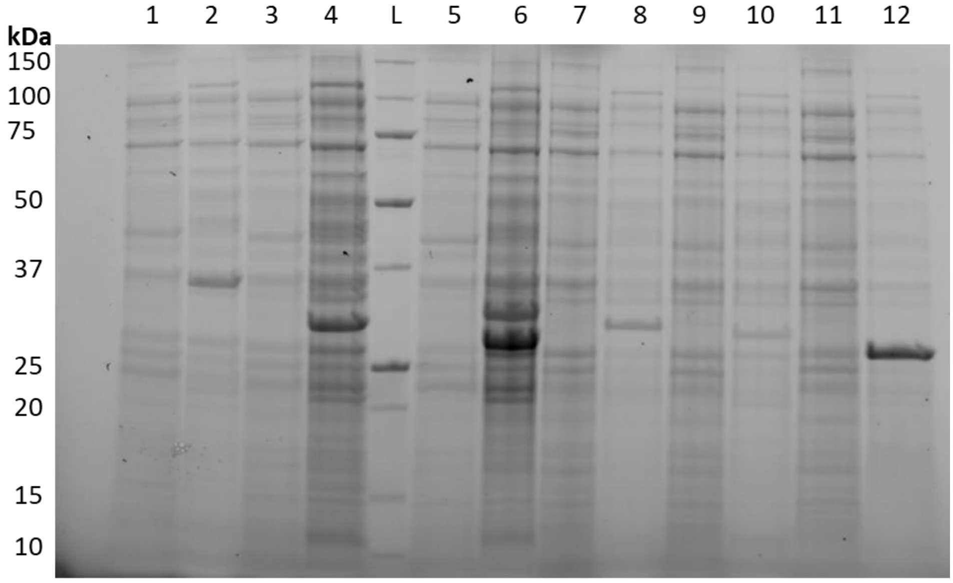


**Figure S1**. SDS-PAGE gel of hits from the EnzymeMiner search before and after IPTG-induction. **1:** *Alu*RedAm (29 kDa) before induction. **2:** *Alu*RedAm after induction. **3:** *Rhod*RedAm before (29 kDa) induction. **4:** *Rhod*RedAm after induction. **5:** *Bac*RedAm (32 kDa) before induction. **6:** *Bac*RedAm after induction. **7:** *Rhod*RedAm (29 kDa) before induction. **8:** *Rhod*RedAm after induction. **9:** *Shy*RedAm (29 kDa) before induction. **10:** *Shy*RedAm after induction. **11:** *Strep*RedAm (29 kDa) before induction. **12:** *Strep*RedAm after induction.

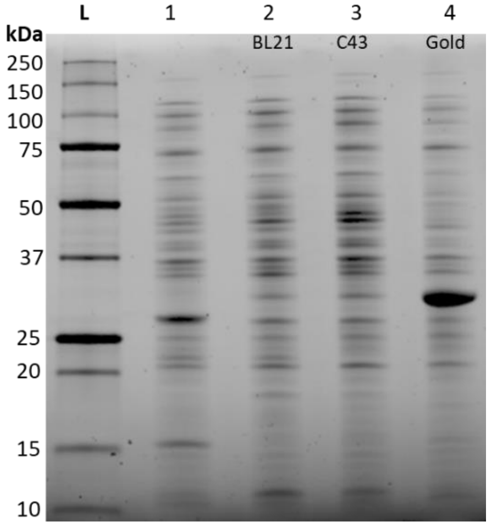


**Figure S2.** SDS-PAGE gel of *Asp*RedAm (32 kDa) cell-free extracts (CFE) after induction with different strains of *E. coli*. **L**: protein ladder. **1**: *Kar*RedAm (24 kDa). **2**: *Asp*RedAm in *E. coli* BL21(DE3). **3**: *Asp*RedAm in *E. coli* C43(DE3). **4**: *Asp*RedAm in *E. coli* BL21 Gold(DE3), shown to give the most *Asp*RedAm.

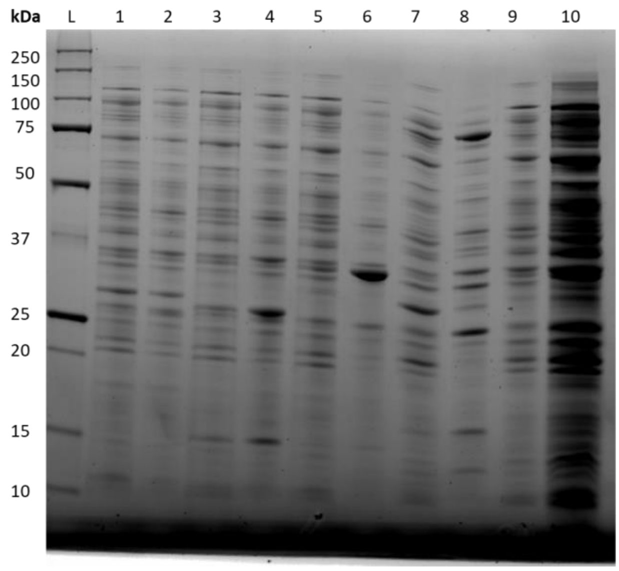


**Figure S3**. SDS-PAGE gel of hits selected from EnzymeMiner after cell lysis. **1:** *Shy*RedAm (29 kDa) CFE. **2:** *Shy*RedAm lysate. **3:** *Kar*RedAm (24 kDa) CFE. **4:** *Kar*RedAm lysate. **5:** *Alu*RedAm (29 kDa) CFE. **6:** *Alu*RedAm lysate. **7:** *Pih*RedAm (32 kDa) CFE. **8:** *Pih*RedAm lysate. **9:** *Strep*RedAm (29 kDa) CFE. **10:** *Strep*RedAm lysate.

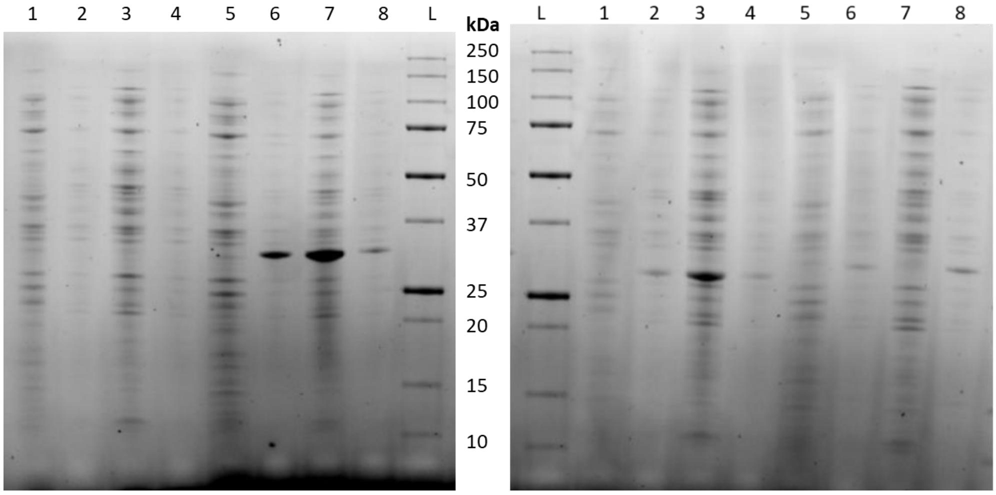


**Figure S4.** SDS-PAGE gels of CFE of bacterial RedAms. **Left: 1**: *Noc*RedAm (30.8 kDa) before induction. **2**: *Noc*RedAm after IPTG induction. **3**: CFE. **4**: cell lysate. **L**: protein ladder. **5**: *Ryt*RedAm before induction. **6**: *Ryt*RedAm (31.4 kDa) after IPTG induction. **7**: CFE. **8**: cell lysate. **Right: 1**: *Rop*RedAm before induction. **2**: *Rop*RedAm (28.7 kDa) after IPTG induction. **3**: CFE. **4**: cell lysate. **5**: *Roc*RedAm before induction. **6**: *Roc*RedAm after IPTG induction. **7**: CFE. **8**: cell lysate. **L**: protein ladder.

## 1.3. Affinity chromatography purification of *Asp*RedAm and *Ryt*RedAm

Two buffers were prepared: 50 mM Tris-HCl pH 8.0, 300 mM NaCl, 1 mM $MgCl_2$ with 30 mM imidazole for the binding buffer, and with 300 mM imidazole for the elution buffer. The buffers were filtered and degassed before use. The cell pellets were thawed and suspended in 5 mL/$g_{wcw}$ binding buffer. After adding a spatula tip of deoxyribonuclease (DNase), $MgCl_2$, lysozyme from chicken egg white (Sigma Aldrich) and a pill of cOmplete$^{TM}$ Mini EDTA-free Protease Inhibitor (Merck), 3 $g_{wcw}$ of cells were disrupted using a Constant Systems Continuous Flow Cell Disrupter CF1 (22 kpsi) and clarified by centrifugation (32,000 × *g*, 30 min, 4 °C).

The cell-free extract (CFE) was then filtered (0.2 µm) and purification was performed by immobilized-metal affinity chromatography (IMAC) using a Ni-NTA 5 mL HisTrap$^{TM}$ FF crude column (GE Healthcare, Chicago, Illinois, U.S.) on a Bio-Rad NGC Chromatography system. After equilibrating the column with binding buffer at 5 mL/min, the CFE was loaded on the column with a velocity of 3 mL/min. Then, the

column was flushed with 10 column volumes (CVs) of binding buffer at 3 mL/min. After 10 CVs, or when the absorbance at 280 nm reached near zero, 100% of the elution buffer was pumped through the column at 3 mL/min. Elution fractions were collected, pooled, and kept on ice until desalted with a PD10 column (GE Healthcare). Enzyme concentration was determined by the Uptima bicinchoninic acid (BC) assay (Interchim, Montluçon, France).

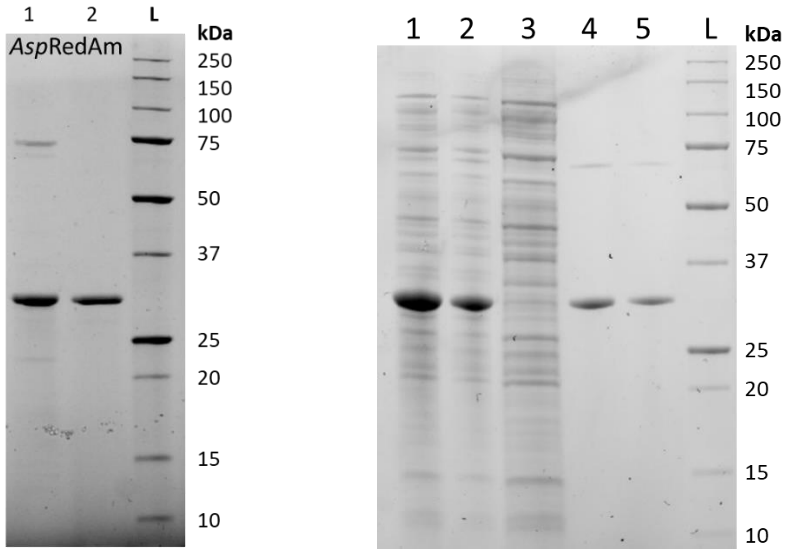


**Figure S5.** SDS-PAGE gels of IMAC purification of *Asp*RedAm and *Ryt*RedAm. **Left: 1**: *Asp*RedAm (32 kDa) elution, 180 mM imidazole. **2**: *Asp*RedAm elution, 300 mM imidazole. **L**: protein ladder. **Right: 1**: CFE *Ryt*RedAm. **2**: cell lysate after disruption. **3**: IMAC flowthrough. **4-5**: *Ryt*RedAm (31.4 kDa) elution, 300 mM imidazole. **L**: protein ladder.

## 1.4. Size exclusion chromatography purification of *Ryt*RedAm

Peak fractions after affinity chromatography containing protein of the correct molecular weight (32 kDa) were pooled. Size exclusion chromatography (SEC) was conducted on an ÄKTA pure Protein Purification System. The pooled fractions were loaded on a HiLoad 16/600 Superdex 75 pg Cytiva column and eluted using buffer containing 100 mM $KP_i$ pH 7.0, 300 mM NaCl and 10% v/v glycerol. Peak fractions containing protein of the correct molecular weight were pooled and centrifugally concentrated, using 10 kDa MWCO concentrator (VivaSpin20™ Göttingen, Germany).

Protein concentration was determined by absorbance at 280 nm using the Beer-Lambert law. Proteins were concentrated to 13, 26, 36 and 65 mg/mL. Proteins were mixed with 5 mM $NAD(P)^+$. Initial screening of crystallization conditions was performed using commercially available INDEX (Hampton Research), PACT premier and CSSI/II (Molecular Dimensions) screens in 96-well sitting drop trays.

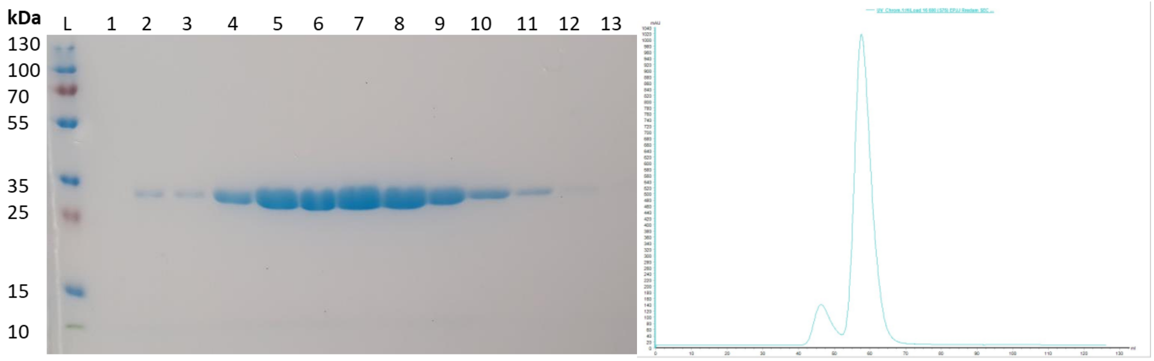


**Figure S6.** SDS-PAGE gel (**left**) and SEC chromatogram (**right**) of the SEC purification of *Ryt*RedAm. **L**: protein ladder. **1**: void volume. **2**: fraction minor peak. **3-13**: fractions major peak.

## 1.5. Expression and purification of *Ryt*RedAm for X-ray crystallography

The sequence encoding *Ryt*RedAm was subcloned into the pETYSBLIC-3C plasmid using established protocols.[1] The LIC3C plasmid containing the gene for *Ryt*RedAm was used to transform *E. coli* BL21(DE3) competent cells for gene expression. Pre-cultures were grown in LB-medium (10 mL) containing 30 µg/mL kanamycin for 18 h at 37 °C with shaking at 180 rpm. 0.5 L TB cultures were inoculated with the pre-culture (10 mL) and incubated at 37 °C, with shaking at 230 rpm until an $OD_{600}$ of 0.7 was reached. Gene expression was induced by addition of IPTG (0.5 mM) and shaking was continued overnight at 16 °C and 230 rpm. The cells were then harvested by centrifugation (5000 × *g*, 20 min) and resuspended in 0.1 M Tris-HCl buffer pH 8.0 containing 300 mM NaCl and 30 mM imidazole (His Buffer A). Cells were disrupted and centrifuged (20,000 × *g*, 1 h, 4 °C) to yield a clear lysate.

The *N*-terminal His6-tagged protein was purified by IMAC using a Ni-NTA column, followed by size exclusion chromatography (SEC). The lysate was loaded onto a pre-equilibrated HisTrap™ FF crude 5 mL column, followed by washing with His Buffer A. The bound protein eluted with a step profile with 500 mM imidazole. Fractions were analyzed by 12% acrylamide SDS-PAGE (**Figure S7A**). Fractions containing *Ryt*RedAm were pooled, HRV3C protease (1:50 ratio) added in order to cleave the histidine tag, and the protein dialyzed overnight against His buffer A. The cleaved *Ryt*RedAm was loaded onto the aforementioned IMAC column, with only the flow-through being collected. His-tag free *Ryt*RedAm was centrifugally concentrated (10 kDa MWCO Vivaspin) and loaded onto a HiLoad 16/600 Superdex 75 gel filtration column pre-equilibrated with buffer containing 50 mM HEPES pH 7.0 and 300 mM NaCl. Fractions were analyzed by 12% acrylamide SDS-PAGE (**Figure S7B**). The concentrated protein sample after gel filtration was used for crystallization screening.

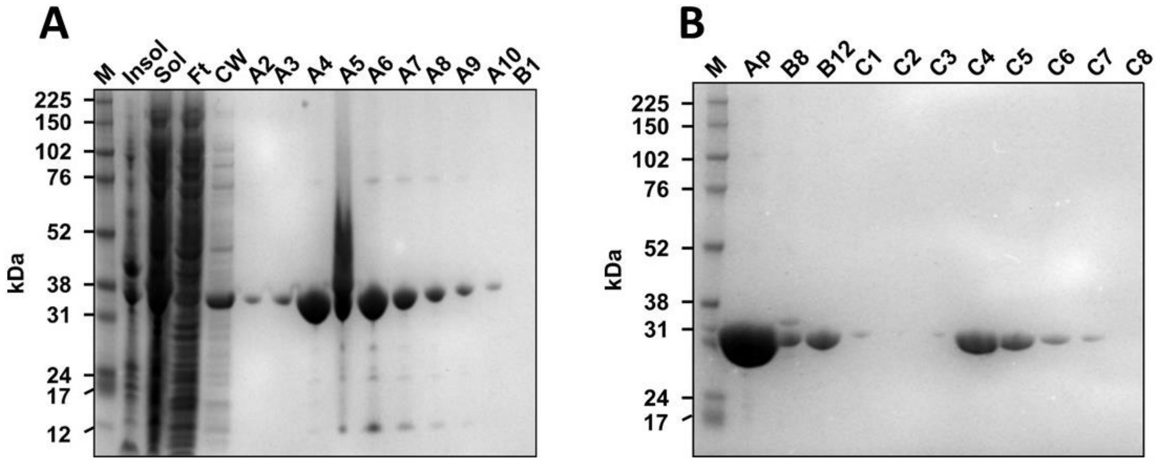


**Figure S7.** 12% acrylamide SDS-PAGE gels of the Ni-NTA column **A** and gel filtration column **B** showing *Ryt*RedAm at approximately 31 kDa.

## 1.6. Crystallization, data collection and structure solution and refinement

Initial screening of crystallization conditions was performed using commercially available INDEX (Hampton Research), PACT premier and CSSI/II (Molecular Dimensions) screens in 96-well sitting drop trays. Optimization was carried out in a 48-well sitting-drop format to obtain crystals for X-ray diffraction studies. For co-crystallization experiments, a 0.1 M stock solution of cofactor $NADP^+$ in water was prepared and cofactor added to a final concentration of 2 mM.

Crystals of both apo- *Ryt*RedAm and the ADP-2'-ribose phosphate (ADP-2RP) complex were grown using *Ryt*RedAm concentrated to 35 mg/mL in 50 mM HEPES buffer at pH 7.0 containing 300 mM NaCl. The best crystals were obtained in conditions containing 25% (w/v) PEG 3350 and 0.2 M $MgCl_2$ (hexahydrate) in bis-Tris buffer pH 6.5. Crystals for diffraction testing were harvested directly into

liquid nitrogen with nylon CryoLoops™ (Hampton Research), using the mother liquor without any further cryoprotectant.

**Data collection, structure solution and refinement**

The datasets described in this report were collected at the Diamond Light Source, Didcot, Oxfordshire, U.K. on beamlines I03 and I04. Data were processed and integrated using XDS[2] and scaled using [3] included in the Xia2[4] processing system. Data collection statistics are provided in **Table S2**. All crystals were obtained in space group $P3_221$, with one molecule in the asymmetric unit. The structure of *Ryt*RedAm was solved by molecular replacement using MOLREP[5] and a model generated using AlphaFold[6] as the search model. The structures were built and refined using iterative cycles in Coot[7] and REFMAC.[8] Following building and refinement of the protein and water molecules in the ADP-2RP dataset, residual density was observed in the omit maps at what would be the dimer interface. This could be clearly modelled as ADP-2RP, as no density for the nicotinamide ring and ribose of $NADP^+$ were observed.

The final structures of *Ryt*RedAm and *Ryt*RedAm-ADP-2RP exhibited % $R_{cryst}$ / $R_{free}$ values of 21.8/30.0 and 19.5/24.5 respectively. Refinement statistics for the structures are presented in **Table S2.** The structures of *Ryt*RedAm and *Ryt*RedAm-ADP-2RP have been deposited in the Protein Databank (PDB) with accession codes **9FM8** and **9FM7**, respectively.

**Table S2.** Data collection and refinement statistics for *Ryt*RedAm. Numbers in brackets refer to data for highest resolution shells.

| | ***Ryt*RedAm apo**<br>**21-04-24 - 0574** | ***Ryt*RedAm ADP-2RP**<br>**10-05-24 - 5331** |
|---|---|---|
| Beamline | I03 | I04 |
| Wavelength (Å) | 0.97627 | 0.95374 |
| Resolution (Å) | 53.39-2.38 (2.47-2.38) | 70.78-2.00 (2.05-2.00) |
| Space Group | $P3_221$ | $P3_221$ |
| Unit cell (Å) | a = b = 80.66; c = 82.78; α = β = 90.00°; γ = 120.00° | a = b = 81.73; c = 83.38; α = β = 90.00°; γ = 120.00° |
| No. of molecules in the asymmetric unit | 1 | 1 |
| Unique reflections | 12900 (1319) | 22229 (1631) |
| Completeness (%) | 100.0 (100.0) | 100.0 (100.0) |
| $R_{merge}$ (%) | 0.14 (0.98) | 0.07 (1.14) |
| $R_{p.i.m.}$ | 0.03 (0.22) | 0.02 (0.37) |
| Multiplicity | 19.8 (20.0) | 20.3 (20.6) |
| $\langle I/\sigma(I)\rangle$ | 8.6 (0.6) | 28.0 (3.1) |
| Overall *B* from Wilson plot (Å²) | 47 | 35 |
| $CC_{1/2}$ | 1.00 (0.96) | 1.00 (0.94) |
| $R_{cryst}$/ $R_{free}$ (%) | 21.8/30.0 | 19.5/24.5 |
| r.m.s.d 1-2 bonds (Å) | 0.005 | 0.007 |
| r.m.s.d 1-3 angles (°) | 1.44 | 1.65 |
| Avge main chain B (Å²) | 71 | 48 |
| Avge side chain B (Å²) | 76 | 55 |
| Avge waters B (Å²) | 61 | 50 |
| Avge ligand B (Å²) | - | 82 |

# 2. Carbonyl substrates, amine donors and products.

## 2.1. Substrates and amine donors

**Table S3.** List of carbonyl substrates and amine donors used in this study

| Label | Carbonyl substrates | Label | Amine donors |
|---|---|---|---|
| **1** | cyclohexanone | **a** | ammonium chloride |
| **2** | 2-hexanone | **b** | methylamine |
| **3** | hexanal | **c** | cyclopropylamine |
| **4** | hydrocinnamaldehyde | **d** | propargylamine |
| **5** | 2,5-hexanedione | **e** | allylamine |
| **6** | benzylacetone | **f** | benzylamine |
| **7** | acetophenone | | |
| **8** | 1-indanone | | |
| **9** | β-tetralone | | |
| **10** | ethyl levulinate | | |
| **11** | benzaldehyde | | |
| **12** | 4-fluorophenylacetone | | |
| **13** | 2-heptanone | | |

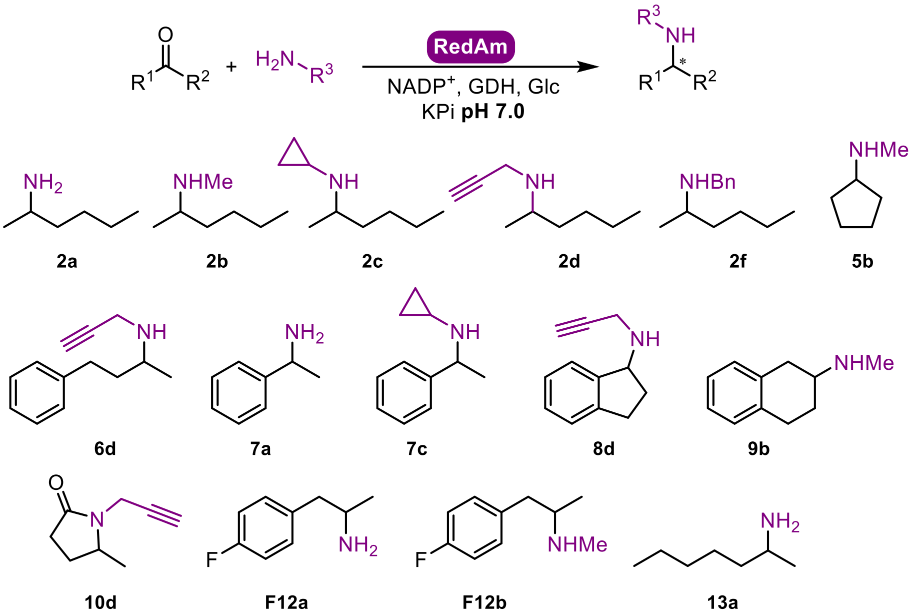


**Figure S8.** Targeted amine products for which the *Ryt*RedAm showed <2% or no measurable conversion.

## 2.2. Amine products synthesis

The synthesis of cyclopropylcyclohexanamine (**1c**, 824-82-8), *N*-2-propyn-1-ylcyclohexanamine (**1d**, 18292-76-7), *N*-cyclopropylbenzenepropanamine (**4c**, 18381-62-9), *N*-2-propyn-1-ylbenzenepropanamine (**4d**, 56862-31-8), *N*-allylbenzenepropanamine (**4e**, 528812-92-2) and 5-methyl-1-(2-propyn-1-yl)-2-pyrrolidinone (**10d**, 18327-34-9) was performed as previously reported.[9]
In dry tetrahydrofuran (THF), 2 mmol of ketone/aldehyde, 2.2 mmol amine, 3 mmol sodium triacetoxyborohydride and 2 mmol glacial acetic acid were mixed and stirred under $N_2$ at room temperature (20 °C) overnight. The reaction was quenched with 10 mL of 10 M NaOH. The reaction mixture was mixed with EtOAc (10 mL) and the organic phase was separated. This step was repeated, the organic phases were combined and extracted with 1M HCl (3 × 10 mL). the aqueous phase was basified to pH 12 with a 5 M NaOH solution. The product was extracted into EtOAc (2 × 20 mL), dried with $MgSO_4$ and the solvent was removed under reduced pressure on a rotary evaporator to afford the aforementioned amines.

The synthesis of 1-cyclopropyl-5-methyl-2-pyrrolidinone (**10c**, 1351473-78-3) and 5-methyl-1-(2-propyn-1-yl)-2-pyrrolidinone (**10d**, 18327-34-9) was performed based on literature.[9] In dry THF, 1 mmol of ketone, 2 mmol of amine, 2 mmol of acetic acid and 1.4 mmol of sodium triacetoxyborohydride were mixed and stirred under $N_2$ at room temperature overnight. The mixture was quenched with 1 M NaOH (5 mL) and extracted with EtOAc (3 × 5 mL). The organic layers were combined, dried over $MgSO_4$ and the solvent evaporated under reduced pressure to afford crude amine products. Yields of synthesized amines varied between 30 and 50% isolated yield. The synthesized compounds were confirmed by NMR. Products *N*-methylhexylamine (**3b**, 35161-70-7), *N*-hexylcyclopropanamine (**3c**, 1040067-31-9), *N*-allylhexan-1-amine (**3e**, 22774-71-6), *N*-methylbenzylamine (**11b**, 103-67-3) and *N*-benzylcyclopropanamine (**11c**, 13324-66-8) were obtained via biotransformations and confirmed by GC-MS (see section 5.3).

## 2.3. *N*-methylation of (*S*)-α-methylbenzylamine

To assign the enantiomer obtained from product *N*-methyl-1-phenethylamine (**7b**, 32512-24-6), *N*-methylation of (*S*)-α-methylbenzylamine was performed as described by Aleku *et al.*[9] 2 mg of the primary amine was dissolved in 200 µL methanol, then 100 µL of 16% v/v formaldehyde in $H_2O$ was added, and the mixture was shaken at room temperature (20 °C) at 800 rpm for 1 h. The reaction mixture was cooled on ice, and 5 mg of $NaBH_4$ was added slowly. 200 µL $dH_2O$ was added, and the reaction mixture was extracted with 1 mL $CH_2Cl_2$. The organic layer was dried with $MgSO_4$ and the corresponding secondary amine with known configuration was analyzed by GC-FID without further purification.

## 3. RedAm activity assays

For specific activity measurements, 4 mL UV grade polymethylmethacrylate (PMMA) plastic cuvettes were used to monitor the decrease of NAD(P)H at a wavelength of 340 nm on a Cary 60 UV-Vis spectrophotometer. The extinction coefficient of NAD(P)H used was $\varepsilon_{340\ nm}$ = 6220 $M^{-1}cm^{-1}$,[10] and $\varepsilon_{370\ nm}$ = 2.216 $M^{-1}cm^{-1}$ (for kinetic parameter measurements at higher NADH concentrations).[9]

Carbonyl substrates were prepared fresh as a 1 M stock solution in DMSO. Amines were prepared fresh in 100 mM $KP_i$ buffer pH 7.0, titrated with a solution of 6 M HCl to adjust the pH to 7.0. NAD(P)H stock solutions were prepared fresh in the mentioned buffer as a 10 mM concentration, confirmed by UV spectrophotometry at 340 nm.

For buffers at different pHs the following salts were used: sodium acetate-HCl pH 5, pyridine-HCl pH 5.5, KPi pH 6-8, MOPS-NaOH pH 6.5-7.5, Tris-HCl pH 7.5-9, glycine-NaOH pH 9.5-10. Buffer pHs were thermodynamically corrected using Buffer Calculator© (Prof. Robert J. Beynon http://phbuffers.org/BufffferCalc/Buffer.htm, checked on 22/05/2024).

### 3.1. CFE activity of RedAm hits

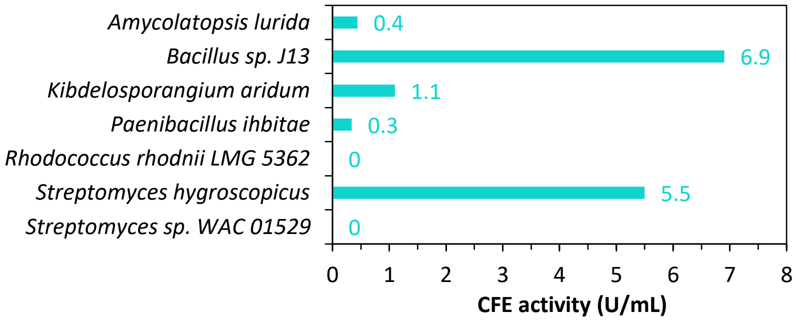


**Figure S9.** CFE reductive amination activity of RedAms from the EnzymeMiner search (*Alu*RedAm, *Bac*RedAm, *Kar*RedAm, *Pih*RedAm, *Rhod*RedAm, *Shy*RedAm, *Strep*RedAm). Conditions: 100 mM $KP_i$ pH 7.0, 10 mM hexanal, 100 mM allylamine, 1% v/v DMSO, 0.2 mM NADPH, 75 µL CFE, 25 °C.

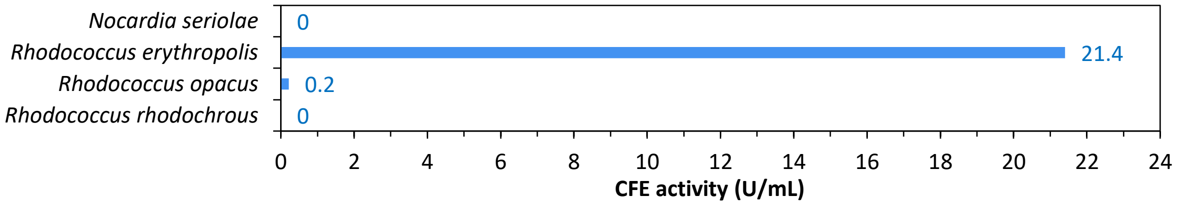


**Figure S10.** CFE reductive amination activity of RedAms from the HMM search in the *Rhodococcus* genome (*Noc*RedAm, *Ryt*RedAm, *Rop*RedAm, *Roc*RedAm). Conditions: 100 mM $KP_i$ pH 7.0, 10 mM hexanal, 100 mM allylamine, 1% v/v DMSO, 0.2 mM NADPH, CFE, 25 °C.

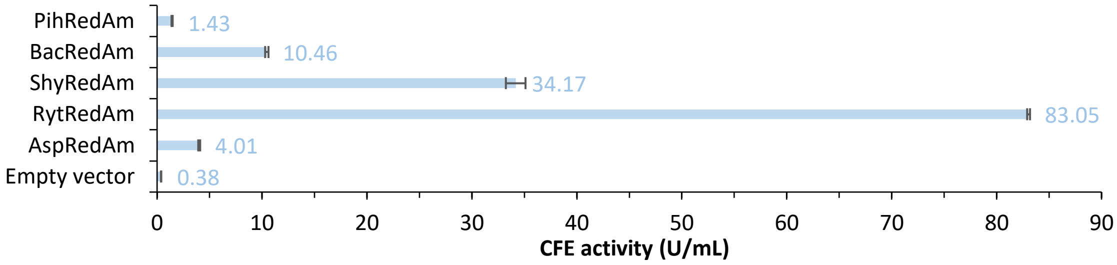


**Figure S11.** CFE reductive amination activity of soluble and best active RedAm hits, compared with *Asp*RedAm and an empty pET28(+) vector. Conditions: 100 mM $KP_i$ pH 7.0, 10 mM hexanal, 100 mM allylamine, 1% v/v DMSO, 0.2 mM NADPH, CFE, 25 °C

## 3.2. Steady-state kinetics of *Ryt*RedAm with NADH and NADPH

Steady-state kinetics were analyzed using IGOR Pro 9 (WaveMetrics). Measurements were plotted onto the equation f(S) = $v_{max}$ × S/($K_M$+S).

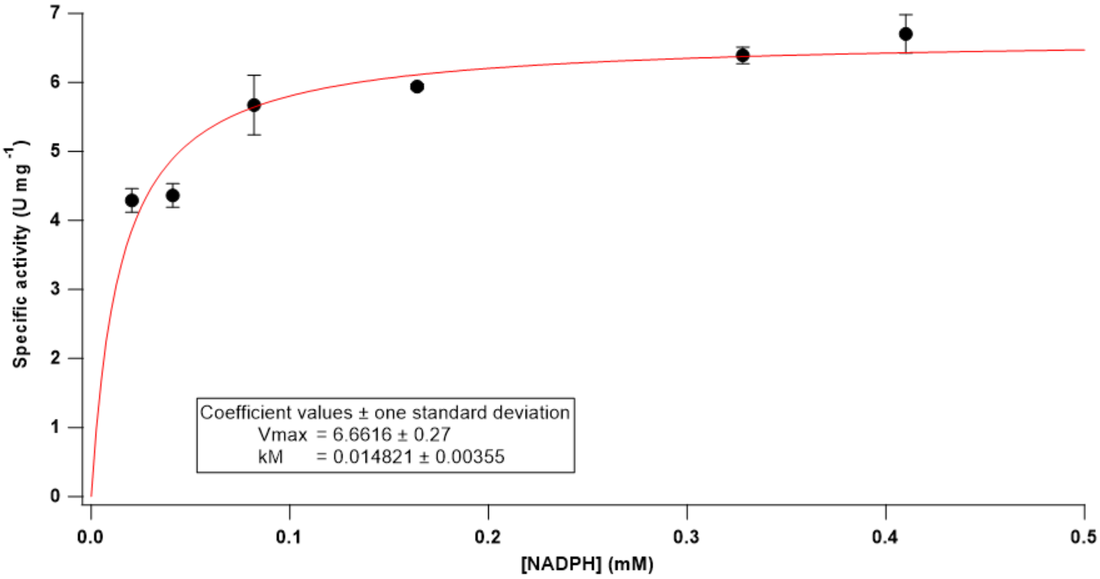


**Figure S12.** Steady-state kinetics for the reductive amination of hexanal and allylamine catalyzed by *Ryt*RedAm with NADPH. Conditions: 100 mM KP$_i$ pH 7.0, 0.025-0.4 mM NADPH, 10 mM hexanal, 100 mM allylamine, 7.5 µg *Ryt*RedAm, 30 °C.

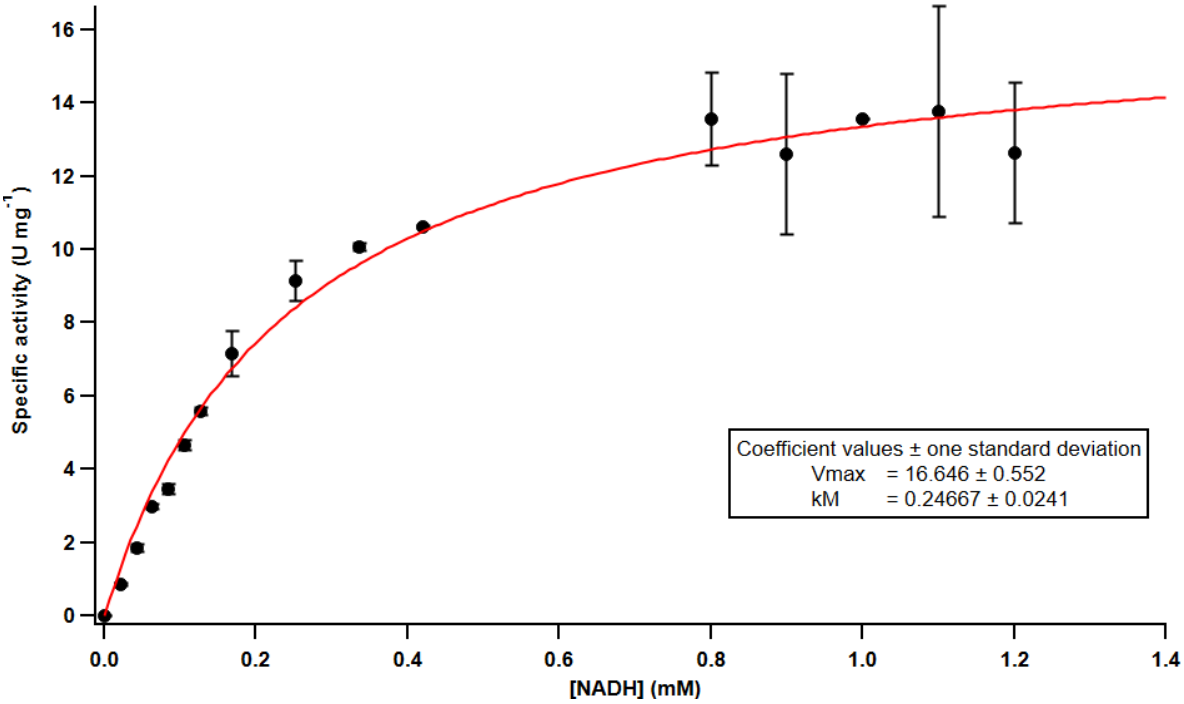


**Figure S13.** Steady-state kinetics for the reductive amination of hexanal and allylamine catalyzed by *Ryt*RedAm with NADH. Conditions: 100 mM KP$_i$ pH 7.0, 0.025-0.5 mM NADH, 10 mM hexanal, 100 mM allylamine, 7.5 µg *Ryt*RedAm, 30 °C.

# 4. RedAm reactions

## 4.1. CFE reactions of RedAm hits

CFE screening of *Pih*RedAm, *Shy*RedAm and *Kar*RedAm (not shown) were performed under two different sets of conditions:
**A:** 100 mM Tris-HCl pH 8.0, 0.2 mM $NADP^+$, 12 mM Glc, 6 U/mL GDH, 50 µL CFE, 10 mM carbonyl substrate, 1-20 eq. amine donor. Stirred at 30 °C and 500 rpm for 24 h
**B:** 100 mM Tris-HCl pH 9.0, 0.4 mM $NADP^+$, 30 mM Glc, 10 U /mL GDH, 100 µL CFE, 10 mM carbonyl substrate, 1-20 eq. amine donor. Stirred at 25 °C and 500 rpm for 24 h.
CFE screening of *Bac*RedAm was only performed under condition set A (**Figure S14**). *Kar*RedAm displayed no reductive amination conversions in both sets of conditions for any substrate-amine combination we tried. To quench the reaction, 0.8 eq. 10 M NaOH was added to the mixtures, and vortexed thoroughly. Then, the reaction mixture was extracted twice with 0.5 µL EtOAc, vortexed and centrifuged (12,000 rpm, 1 min), the organic layer was separated, dried over anhydrous $MgSO_4$, centrifuged, and transferred to a GC vial for GC-FID analysis. Conversions were calculated by the product peak area divided by the sum of the substrate and product peak areas.

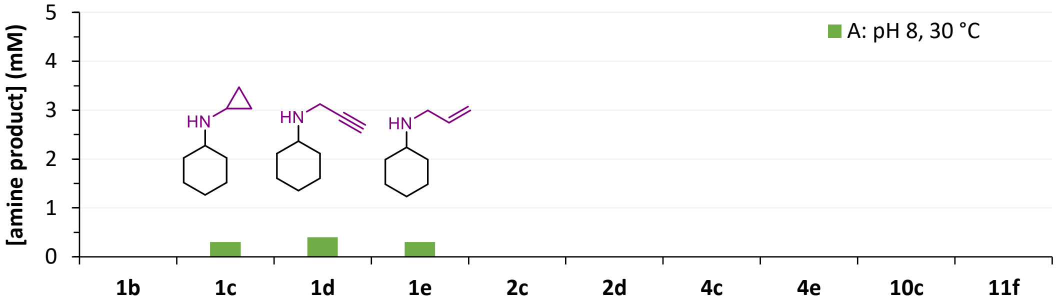


**Figure S14**. *Bac*RedAm CFE-catalyzed reductive amination under reaction conditions **A**.

## 4.2. *Ryt*RedAm 1-hour biotransformation

Reactions were performed in GC glass vials with 100 mM $KP_i$ buffer pH 7.0, 10 mM carbonyl substrate, 100 mM amine donor, 0.2 mM $NADP^+$, 30 mM Glc, 12 U/mL *Bs*GDH, 0.5 mg/mL purified *Ryt*RedAm, 0.5 mL total reaction volume, stirred at 500 rpm at 30 °C for 1 h on an Eppendorf Thermomixer C.

## 4.3. *Ryt*RedAm reaction screening

Reactions were performed in GC glass vials with 100 mM $KP_i$ buffer pH 7.0, 10 mM carbonyl substrate, 10-1000 mM amine donor, 0.2 mM $NADP^+$, 30 mM Glc, 10 U/mL *Bs*GDH, 0.5 mg/mL purified *Ryt*RedAm, 0.5 mL total reaction volume. Reactions were stirred at 500 rpm and 30 °C for 24 h on an Eppendorf Thermomixer C. To quench the reaction, 0.4 mL 10 M NaOH was added and the reaction mixture was extracted with 0.5 mL EtOAc, vortexed, centrifuged (10,000 × *g*, 1 min), the organic layer was isolated, dried over anhydrous $MgSO_4$, centrifuged, decanted to a GC vial for GC-FID analysis. Conversions were determined by the product peak area divided by the sum of the substrate and product peak areas. Control reactions were ran in the same conditions but without *Ryt*RedAm.

## 4.4. *Ryt*RedAm imine reduction screening

Reactions were performed in GC glass vials with 10 mM 2-methyl-1-pyrroline, 0.2 mM $NADP^+$, 30 mM Glc, 10 U/mL *Bs*GDH, 100 mM KPi buffer pH 7.0, 0.5 mg/mL purified *Ryt*RedAm, 0.5 mL total reaction volume. Reactions were stirred at 500 rpm at 30 °C for 24 h on an Eppendorf Thermomixer C. The reaction mixture was quenched, extracted and analyzed as described above. Less than 1% conversion was observed (see GC section 5.2.7).

### 4.5. Reductive amination with cyclohexanone and different amine donor concentrations

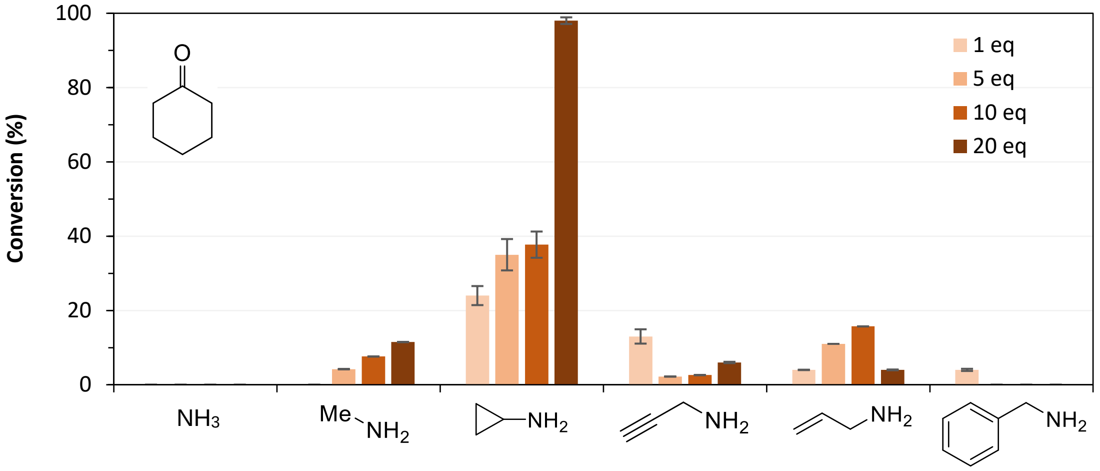


**Figure S15.** Influence of the amine donor concentration for the reductive amination of cyclohexanone **1** catalyzed by *Ryt*RedAm. Conditions: 100 mM KPi pH 7.0, 30 mM Glc, 10 U/mL *Bs*GDH, 10 mM cyclohexanone **1**, 10-500 mM amine **a-f**, 0.2 mM $NADP^+$, 0.5 mg/mL *Ryt*RedAm, stirred at 500 rpm at 30 °C for 24 h.

## 5. GC analyses

### 5.1. GC columns and methods

The following chiral columns were used to determine enantiomeric excess of chiral products; details on the injection temperature, linear velocity, column flow, oven temperature program and retention times can be found for each compound below.

**A. CP-Sil 8 CB** (Agilent Technologies, Santa Clara, California, United States) (25 m × 0.25 mm × 1.20 µm), injection at 340 °C, split ratio 50, linear velocity 30 cm/sec, column flow 1.01 mL/min, nitrogen as carrier gas.

**B. CP-Wax 52 CB** (Agilent Technologies, Santa Clara, California, United States) (25 m × 0.53 mm × 2.0 µm), injection at 250 °C, split ratio 50, flow 4 mL/min, nitrogen as carrier gas.

**C. Hydrodex β-TBDM** (Macherey-Nagel, Düren, Germany), 50 m × 0.25 mm × 0.15 µm, heptakis-(2,3-di-O-methyl-6-O-*t*-butyldimethyl-silyl)-β-cyclodextrin, injection at 250 °C split ratio 50, linear velocity 38 cm/s, column flow 2.23 mL/min, helium as carrier gas.

**Table S4.** List of GC methods, with corresponding methods and retention times.

| | | GC oven program | | | | |
|---|---|---|---|---|---|---|
| **GC column** | **Method** | rate (°C/min), | temp. (°C), | hold (min) | **Compound** | **ret. time (min)** |
| **A CP-Sil 8 CB** | A1 | - | 80 | 3 | Methylamine **b** | 7.8 |
| | | 5 | 100 | 4 | DMSO | 8.3 |
| | | 25 | 345 | 1 | cyclohexylamine **1a** | 9.9 |
| | | | | | cyclohexanol (side product) | 10.8 |
| | | | | | cyclohexanone **1** | 11.4 |
| | | | | | *N*-methylcyclohexylamine **1b** | 12.5 |
| | | | | | dodecane | 16.8 |
| **A** | A2 | - | 80 | 3 | DMSO | 8.3 |
| | | 5 | 100 | 4 | cyclohexanol (side product) | 10.8 |
| | | 10 | 200 | 2.2 | cyclohexanone **1** | 11.4 |
| | | 25 | 345 | 1 | benzylamine **f** | 16.0 |
| | | | | | *N*-2-propyn-1-ylcyclohexanamine **1d** [a] | 18.6 [a] |
| | | | | | *N*-allylcyclohexanamine **1e** | 17.9 |
| | | | | | *N*-cyclopropylcyclohexanamine **1c** [a] | 18.6 [a] |
| | | | | | dodecane | 20.3 |
| | | | | | *N*-benzylcyclohexanamine **1f** | 27.3 |
| **A** | A3 | - | 100 | 3.25 | DMSO | 5.9 |
| | | 5 | 250 | 1 | benzaldehyde **11** | 9.7 |
| | | 20 | 345 | 0 | benzyl alcohol | 11.7 |
| | | | | | *N*-methylbenzylamine **11b** [b] | 12.6 [b] |
| | | | | | dodecane | 16.9 |
| | | | | | *N*-allylbenzylamine **11e** [c] | 17.8 [c] |
| | | | | | *N*-benzylcyclopropanamine **11c** [b] | 18.6 [b] |
| | | | | | *N*-benzylpropargylamine **11d** [b] | 18.7 [b] |
| | | | | | dibenzylamine **11f** | 32.6 |
| **A** | A4 | - | 100 | 1.75 | DMSO | 5.1 |
| | | 10 | 140 | 2 | dodecane | 13.8 |
| | | 5 | 180 | 5 | hydrocinnamaldehyde **4** | 13.2 |
| | | 20 | 345 | 1 | *N*-allylbenzenepropanamine **4e** [a] | 21.8 [a] |
| | | | | | *N*-2-propyn-1-ylbenzenepropanamine **4d** [a] | 22.6 [a] |
| | | | | | *N*-cyclopropylbenzenepropanamine **4c** [a] | 22.7 [a] |
| **B CP-Wax 52 CB** | B1 | - | 80 | 3 | hexanal **3** | 6.7 |
| | | 5 | 150 | 1 | dodecane | 9.2 |
| | | 20 | 250 | 1 | *N*-hexylcyclopropanamine **3c** [b] | 11.2 [b] |
| | | | | | *N*-methylhexylamine **3b** [b] | 13.6 [b] |
| | | | | | *N*-(prop-2-yn-1-yl)hexan-1-amine **3d** [c] | 16.8 [c] |
| | | | | | *N*-allylhexan-1-amine **3e** [b] | 11.2 [b] |
| | | | | | DMSO | 20.2 |
| **C Hydrodex β-TBDM** | C1 | - | 100 | 2 | DMSO | 6.8 |
| | | 5 | 220 | 1 | *N*-methyl-1-phenylethylamine **7b** | 9.5, 9.6 |
| | | | | | ethyl levulinate **10** | 9.8 |
| | | | | | acetophenone **7** | 10.5 |
| | | | | | 1-cyclopropyl-5-methyl-2-pyrrolidinone **10c** | 16.7, 16.8 [a] |
| | | | | | dodecane | 10.7 |
| **C** | C2 | - | 80 | 2 | 2-methyl-1-pyrroline | 10.7 |
| | | 5 | 220 | 1 | 2-methylpyrrolidine | 20.1, 20.5 |
| | | | | | (*R*)-2-methylpyrrolidine | 20.1 |
| | | | | | DMSO | 17.7 |

[a] Based on products of amine synthesis
[b] Based on comparison with negative control, confirmed on GC-MS
[c] Based on negative control

# 5.2. GC chromatograms

## 5.2.1. Amine products from cyclohexanone **1**

### Cyclohexylamine **1a**

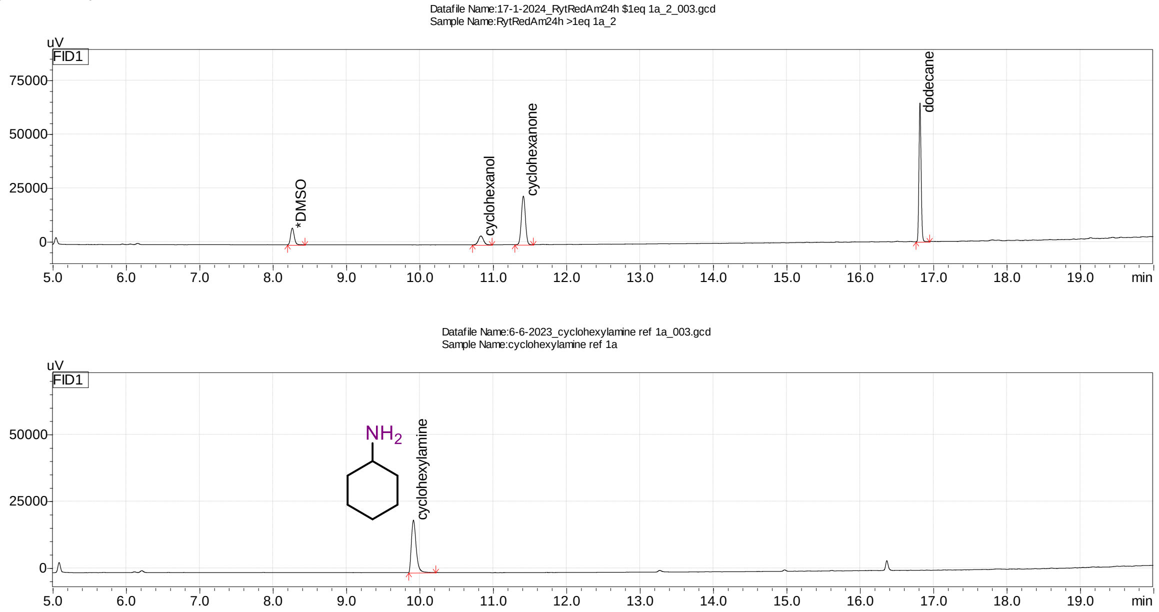


**Figure S16.** GC chromatograms of the *Ryt*RedAm reaction mixture (top) to produce cyclohexylamine **1a** (bottom reference); on CP-Sil 8 CB method A1. No conversion was observed.

### *N*-methylcyclohexylamine **1b**

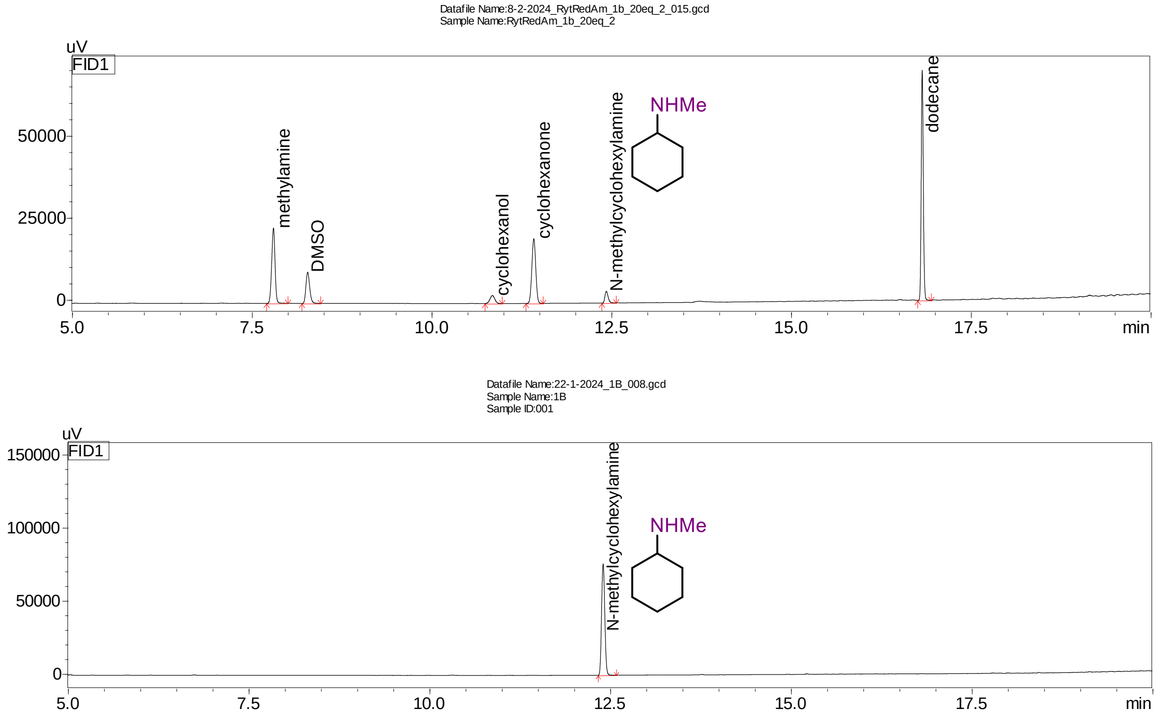


**Figure S17.** GC chromatogram of the *Ryt*RedAm reaction mixture with 20 eq. methylamine to produce *N*-methylcyclohexylamine **1b** (12.4 min), bottom: amine standard; on CP-Sil 8 CB method A1.

## *N*-cyclopropylcyclohexanamine **1c**

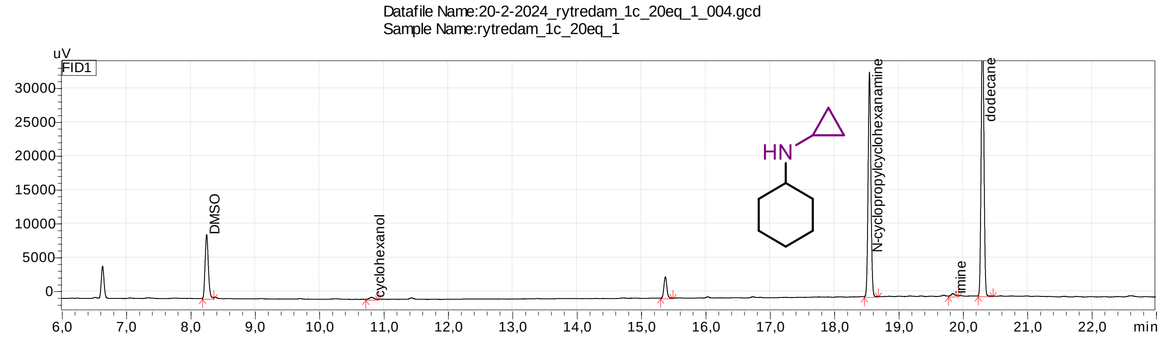


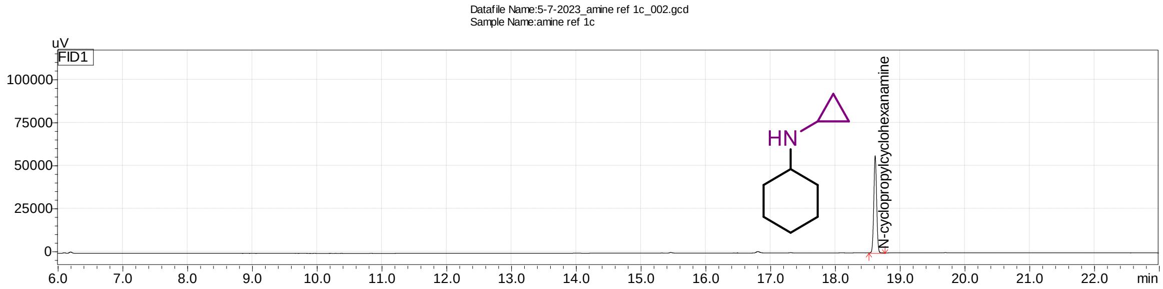


**Figure S18.** GC chromatograms of the *Ryt*RedAm reaction mixture (top) to produce *N*-cyclopropylcyclohexanamine **1c** (bottom amine standard); on CP-Sil 8 CB method A2.

## *N*-2-propyn-1-ylcyclohexanamine **1d**

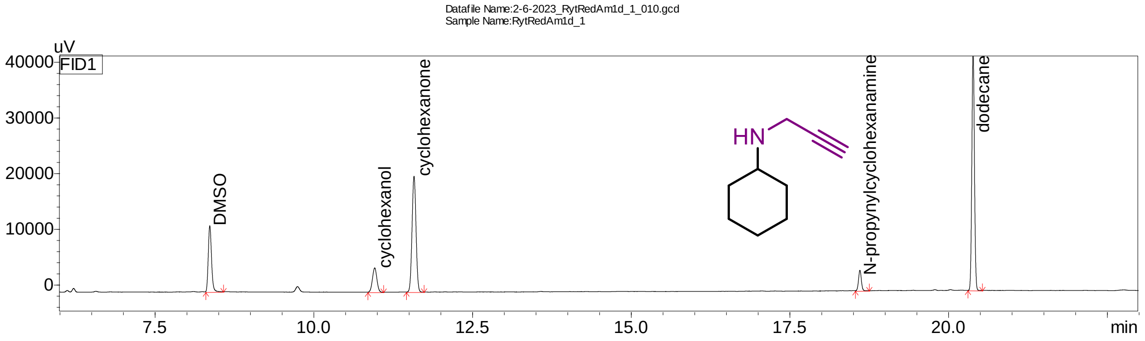


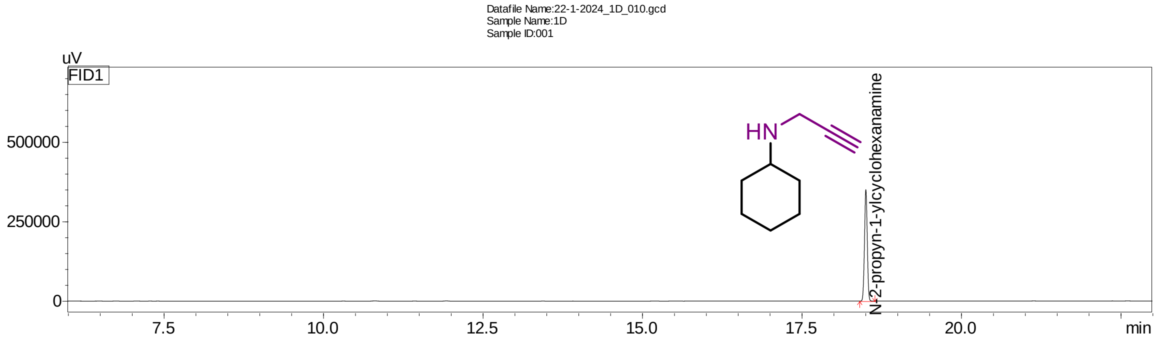


**Figure S19.** GC chromatogram of reaction mixture with *Ryt*RedAm (top) to produce *N*-2-propyn-1-ylcyclohexanamine **1d** (18.6 min). Bottom: amine standard; on CP-Sil 8 CB method A2.

## *N*-allylcyclohexanamine **1e**

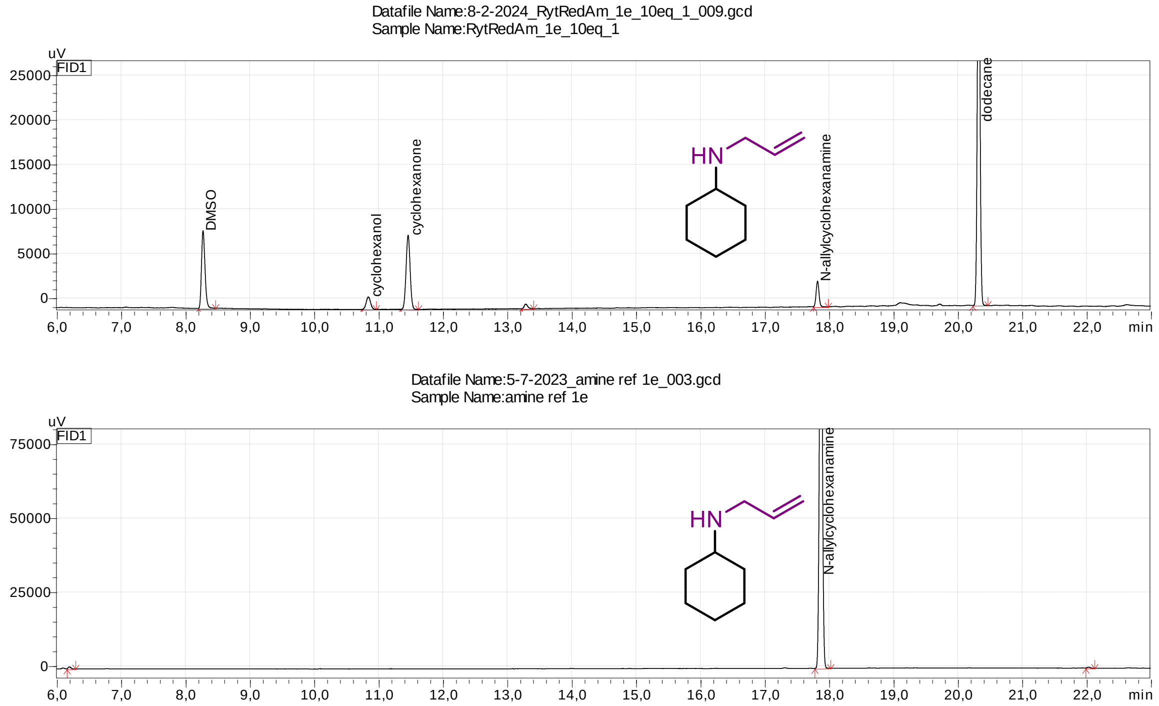


**Figure S20.** GC chromatogram of reaction mixture with *Ryt*RedAm (top) to produce *N*-allylcyclohexanamine **1e** (17.9 min). Bottom: amine standard; on CP-Sil 8 CB method A2.

## *N*-benzylcyclohexanamine **1f**

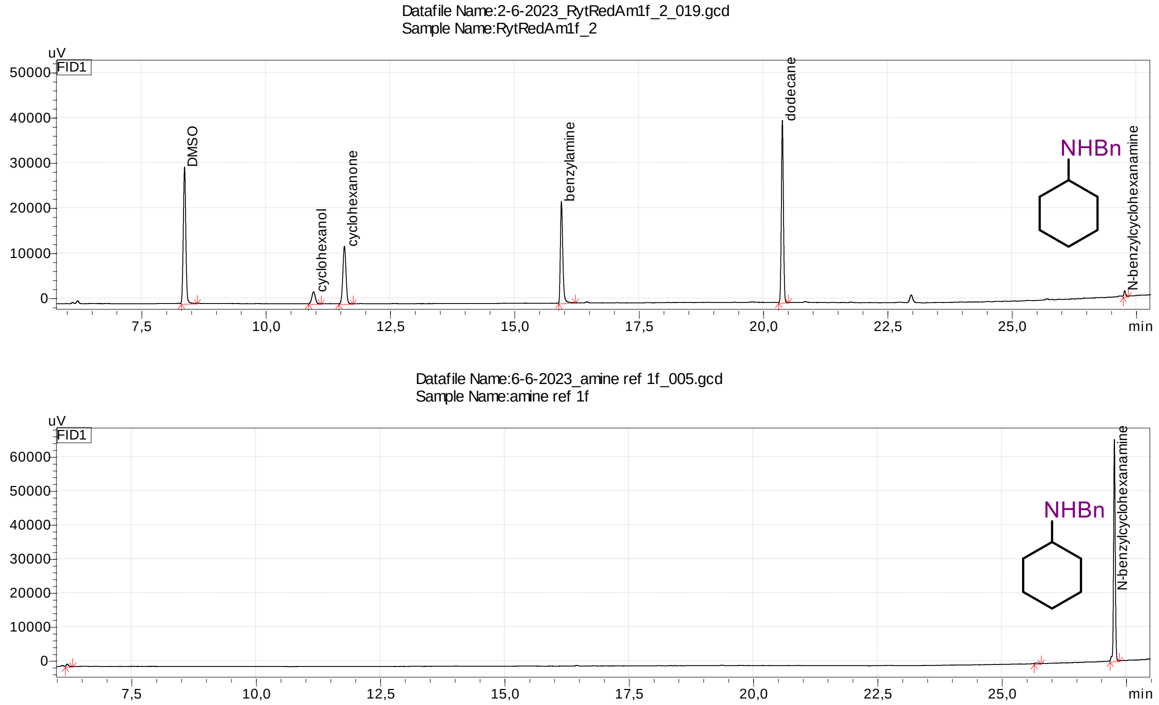


**Figure S21.** GC chromatogram of reaction mixture with *Ryt*RedAm (top) to produce *N*-benzylcyclohexanamine **1f** (27.3 min). Bottom: amine standard; on CP-Sil 8 CB method A3.

### 5.2.2. Amine products from hexanal **3**

### *N*-methylhexylamine **3b**

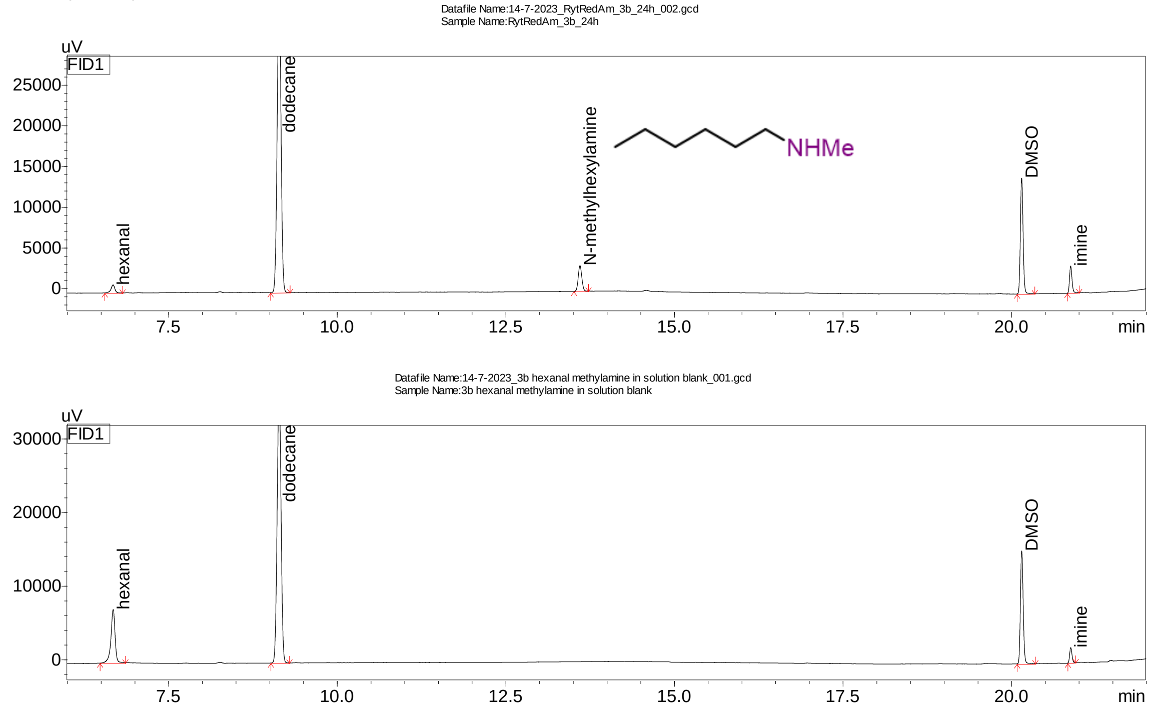


**Figure S22.** GC chromatogram of reaction mixture with *Ryt*RedAm to produce *N*-methylhexylamine **3b** (top) and control reaction without enzyme (bottom); on column CP-Wax 52 CB method B. Expected amine product at 13.6 min.

### *N*-hexylcyclopropanamine **3c**

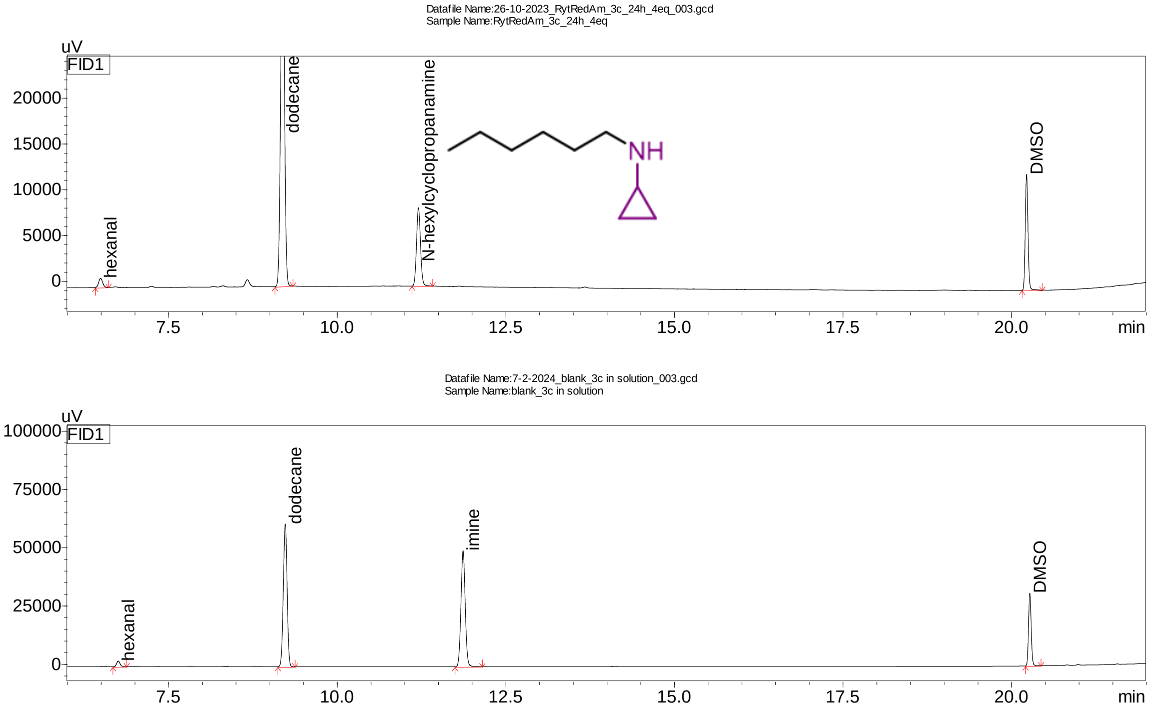


**Figure S23.** GC chromatogram of reaction mixture with *Ryt*RedAm to produce *N*-hexylcyclopropanamine **3c** (top) and control reaction without enzyme (bottom); on column CP-Wax 52 CB method B. Expected amine product at 11.2 min.

## *N*-(prop-2-yn-1-yl)hexan-1-amine **3d**

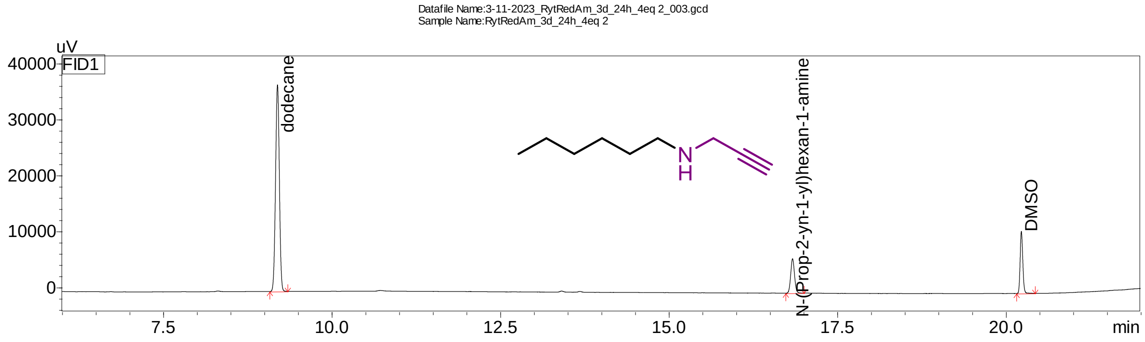


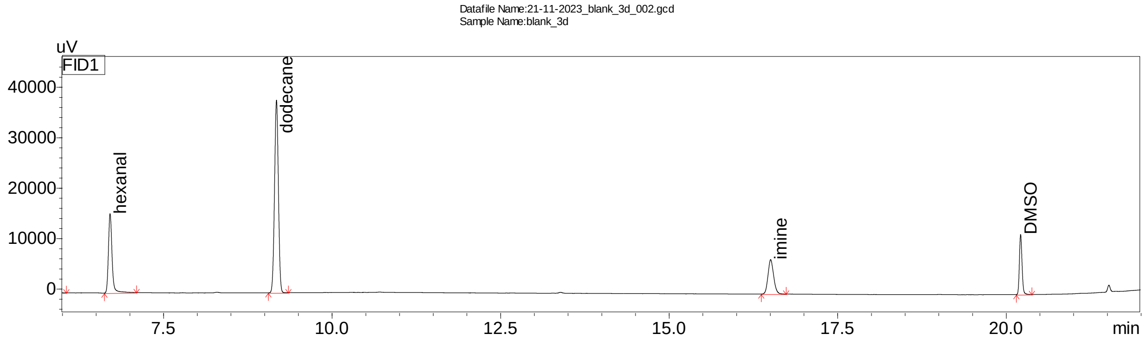


**Figure S24.** GC chromatogram of reaction mixture with *Ryt*RedAm to produce *N*-(prop-2-yn-1-yl)hexan-1-amine **3d** (top) and control reaction (bottom) on column CP-Wax 52 CB method B. Expected amine product at 16.8 min.

## *N*-allylhexan-1-amine **3e**

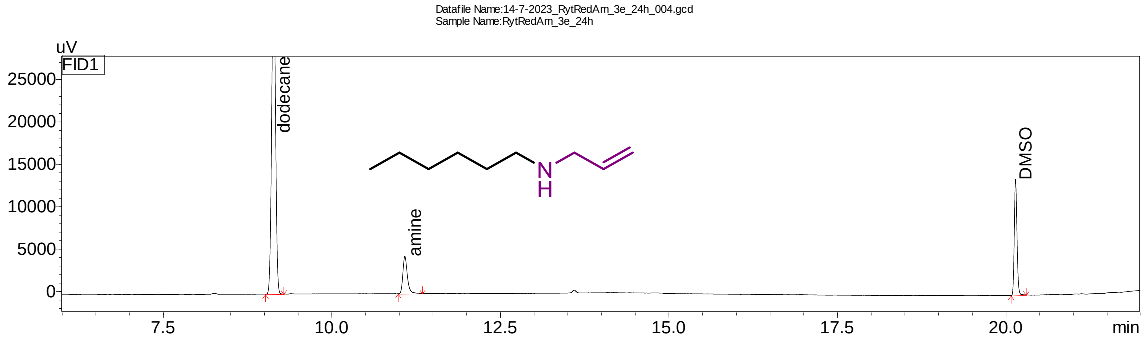


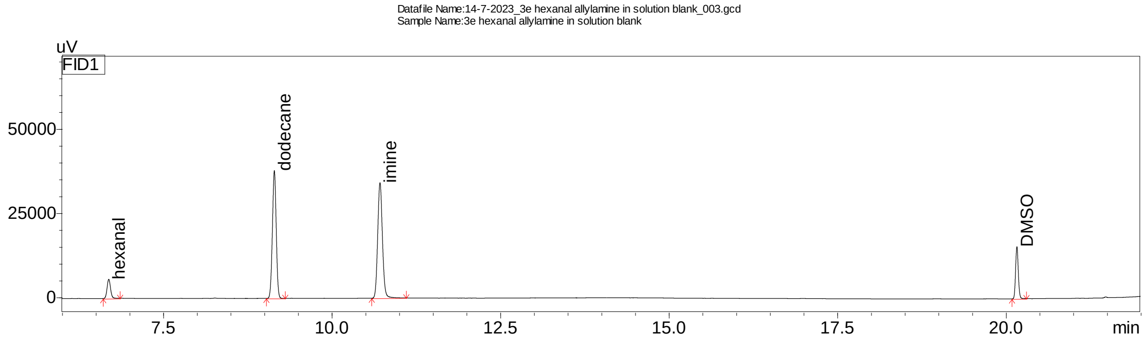


**Figure S25.** GC chromatogram of reaction mixture with *Ryt*RedAm to produce *N*-allylhexan-1-amine **3e** (top) and control reaction without enzyme (bottom); on column CP-Wax 52 CB method B. Expected amine product at 11.2 min.

## 5.2.3. Amine products from hydrocinnamaldehyde **4**

### *N*-cyclopropylbenzenepropanamine **4c**

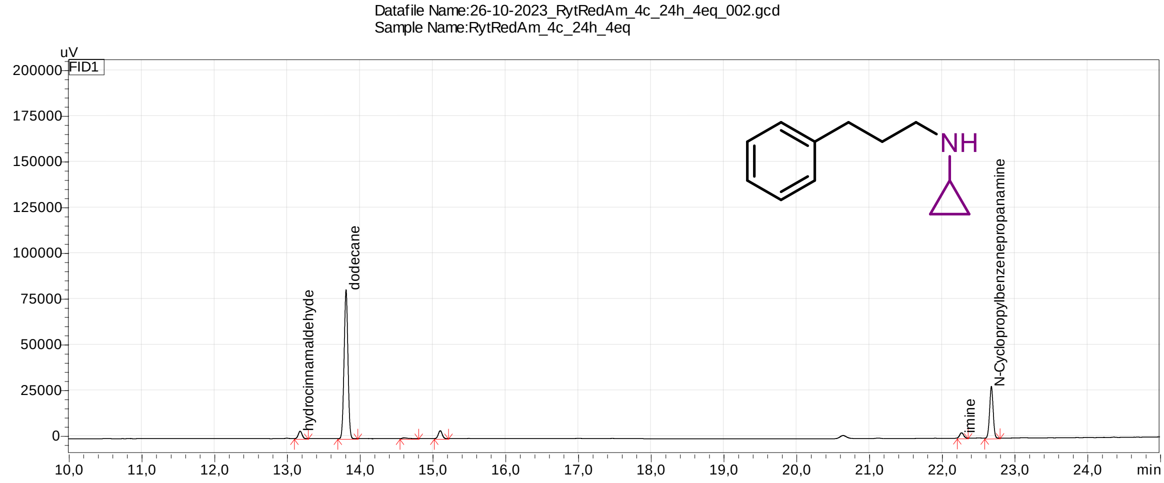


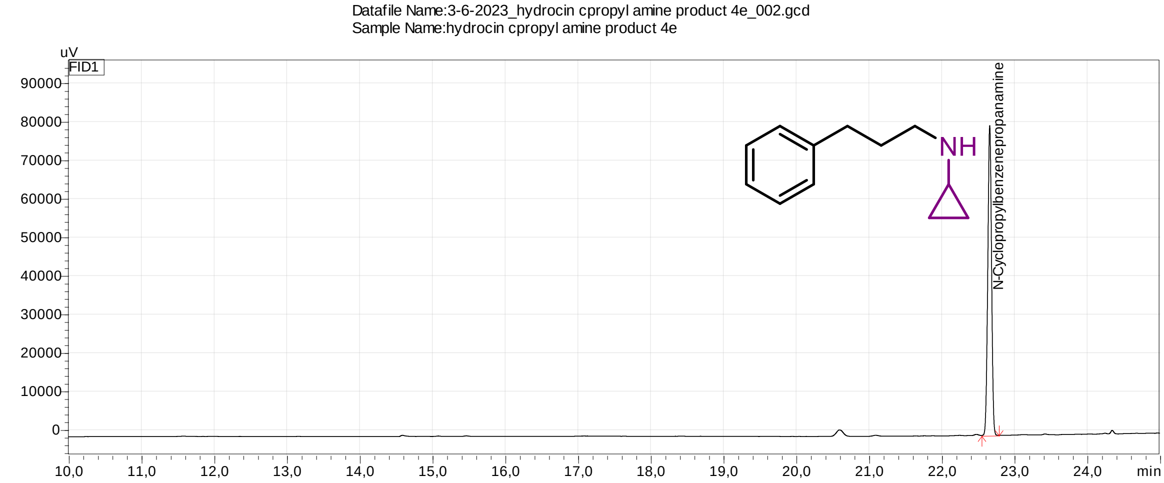


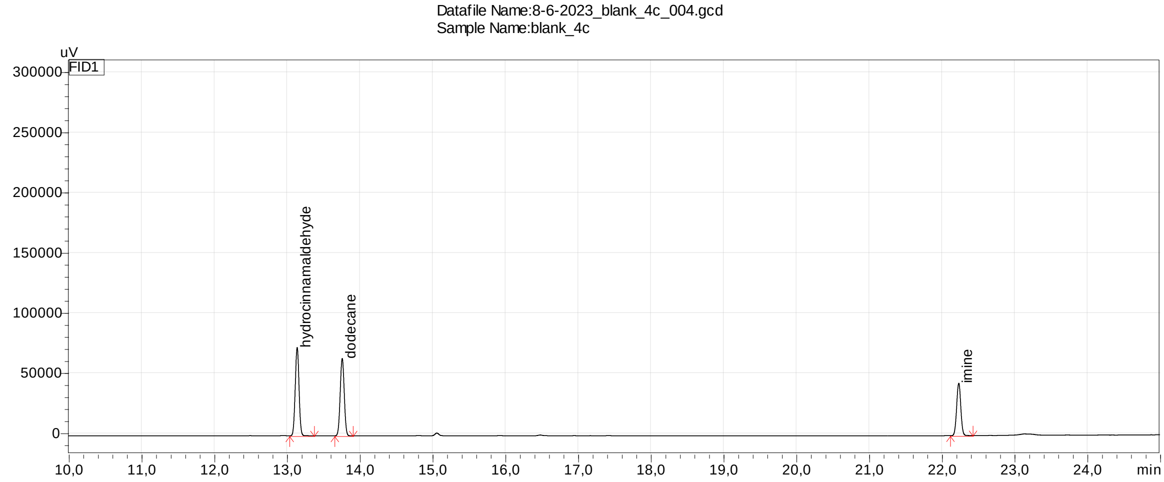


**Figure S26.** GC chromatogram of reaction mixture with *Ryt*RedAm to produce *N*-cyclopropylbenzenepropanamine **4c**. Impurity of hydrocinnamaldehyde at 15.0 min. Expected imine present at 22.3 min. Middle: synthesized product **4c**. Bottom: control reaction without enzyme, impurity of hydrocinnamaldehyde at 15.0 min, expected imine present at 22.3 min; on CP-Sil 8 CB method A4.

## *N*-2-propyn-1-ylbenzenepropanamine **4d**

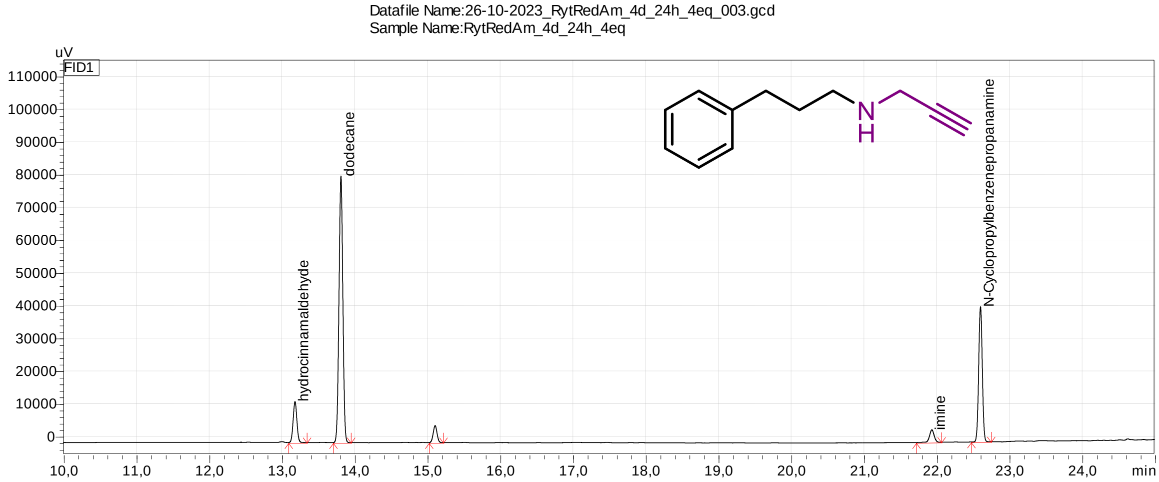


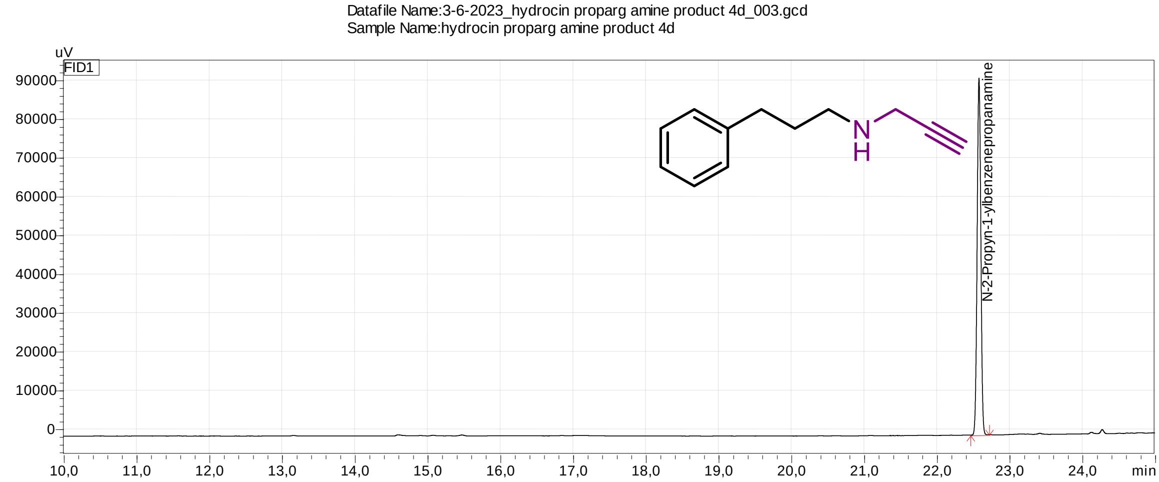


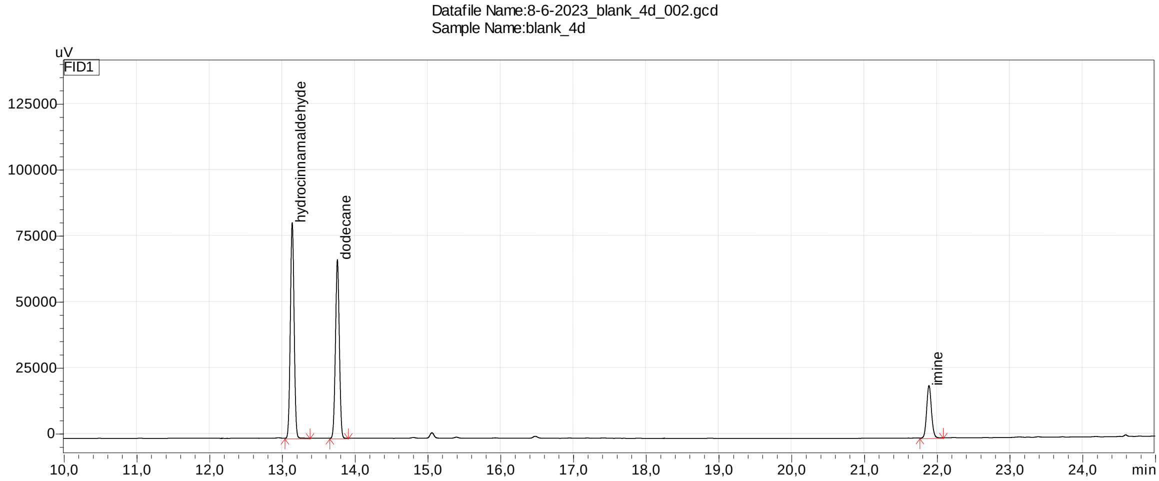


**Figure S27.** GC chromatogram of reaction mixture with *Ryt*RedAm (top) to produce *N*-2-propyn-1-ylbenzenepropanamine **4d**, impurity of hydrocinnamaldehyde at 15.0 min, expected imine present at 21.8 min. Middle: synthesized product **4d.** Bottom: control reaction without enzyme; on CP-Sil 8 CB method A4.

## *N*-2-propen-1-ylbenzenepropanamine **4e**

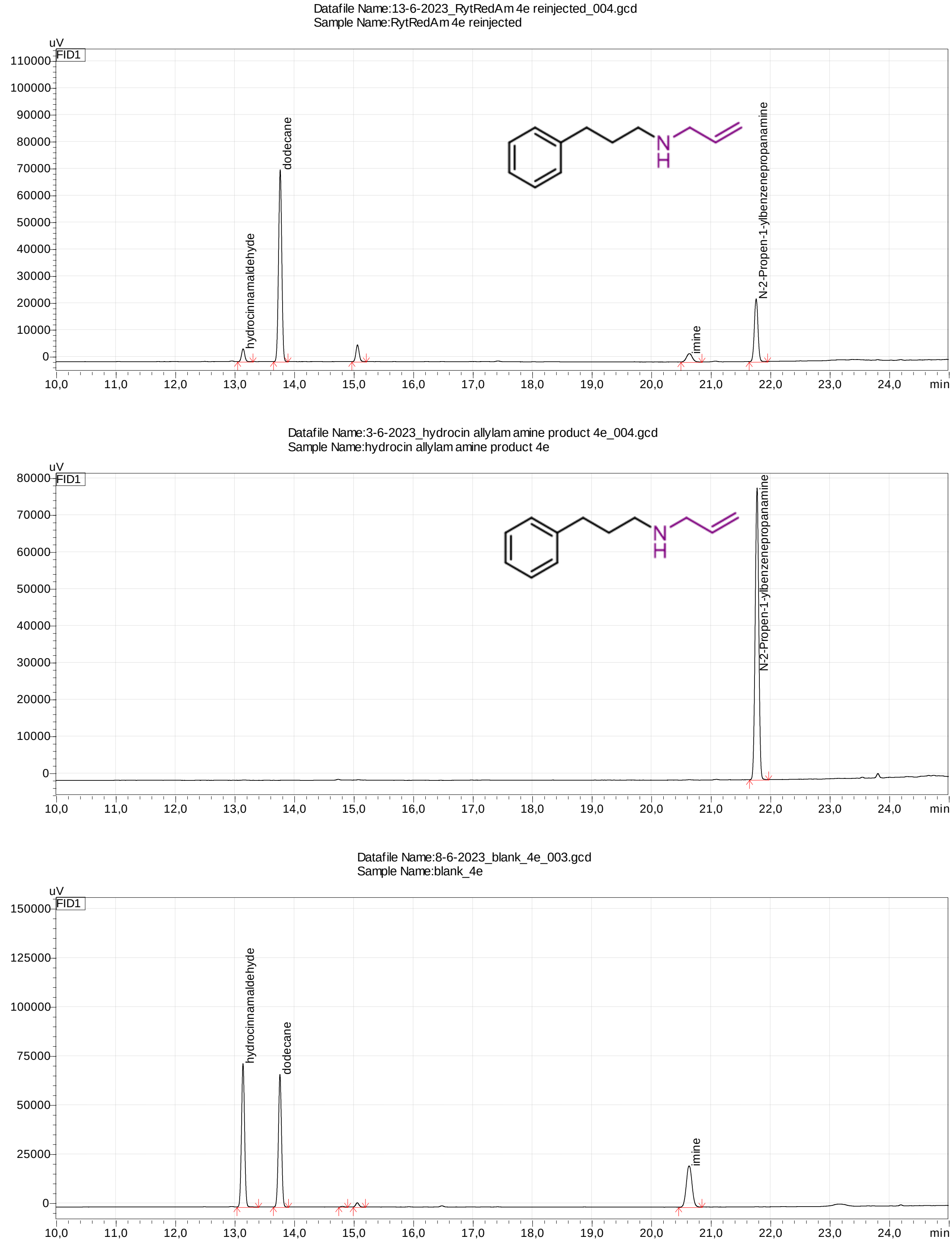


**Figure S28.** GC chromatogram of reaction mixture with *Ryt*RedAm (top) to produce *N*-2-propen-1-ylbenzenepropanamine **4e** Middle: synthesized product **4e**. Bottom: control reaction without enzyme, Impurity of hydrocinnamaldehyde at 15.0 min, expected imine present at 20.7 min; on CP-Sil 8 CB method A4.

## 5.2.4. Amine products from acetophenone **7**

### *N*-methyl-1-phenylethylamine **7b**

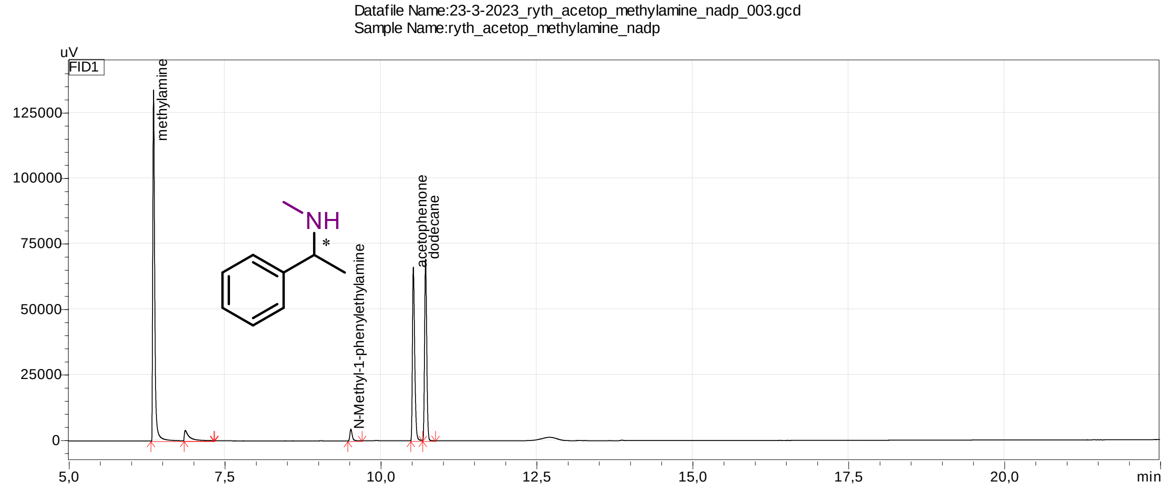


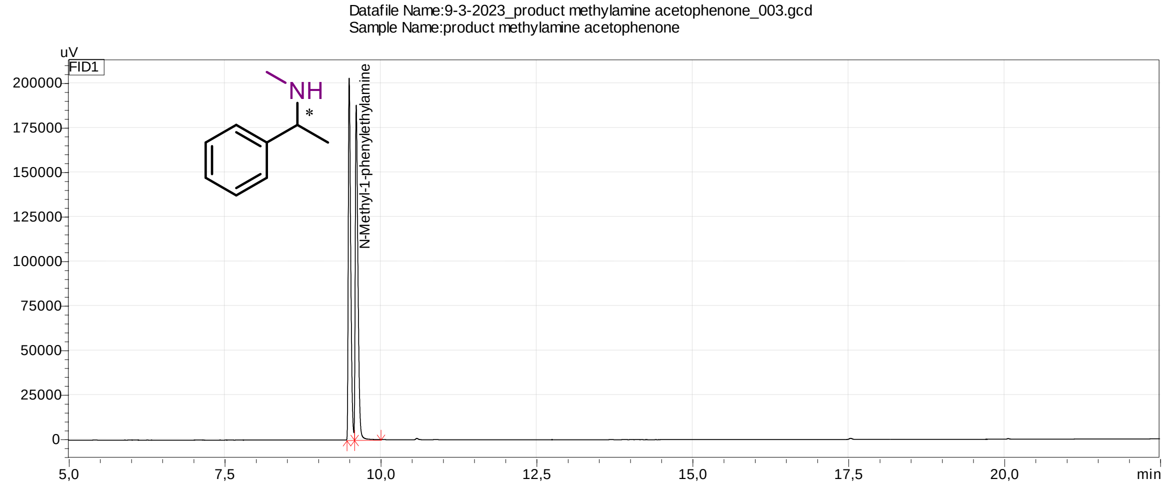


**Figure S29.** GC chromatogram of reaction mixture with *Ryt*RedAm to produce *N*-methyl-1-phenylethylamine **7b**. Methylamine at 6.4 min. Bottom: amine standard **7b**; on Hydrodex β-TBDM method C1.

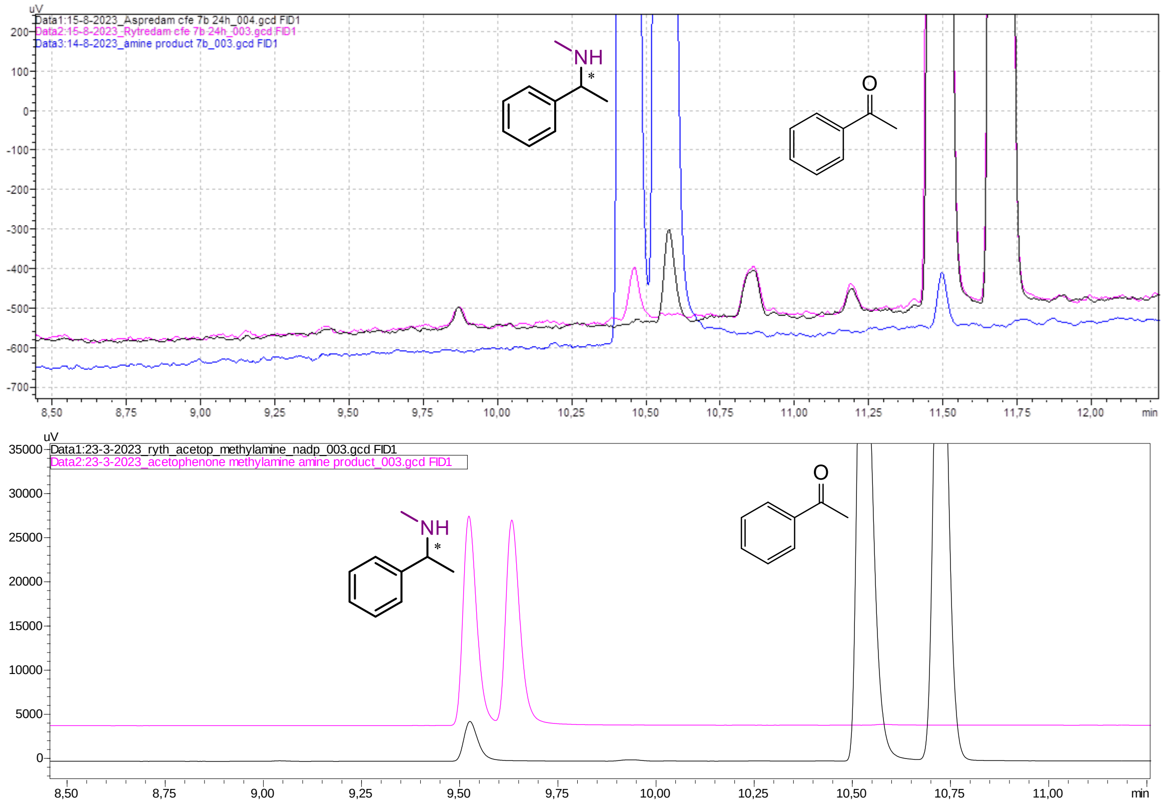


**Figure S30.** GC chromatogram of reaction mixtures to produce *N*-methyl-1-phenylethylamine **7b** by *Asp*RedAm (>99% *ee R*, black), by *Ryt*RedAm (>99% *ee S*, pink) and the racemic **7b** (blue). Bottom: *Ryt*RedAm (>99% *ee S*, black) and the racemic **7b** (pink); on Hydrodex β-TBDM method C1.

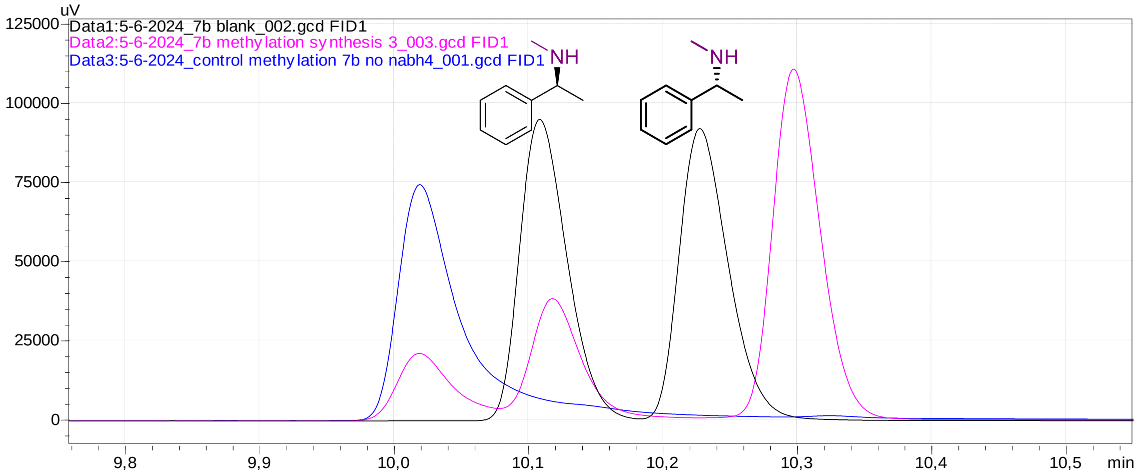


**Figure S31**. GC chromatogram of chemically synthesized racemic standard of *N*-methyl-1-phenylethylamine (black), (*S*)-*N*-methyl-1-phenylethylamine after methylation of (*S*)-*α*-methylbenzylamine (pink), and control reaction without $NaBH_4$ (blue). Expected imine at 10.0 min, expected dimethylated product at 10.3 min; on Hydrodex β-TBDM method C1.

## 5.2.5. Amine products from ethyl levulinate **10**

## 1-cyclopropyl-5-methyl-2-pyrrolidinone **10c**

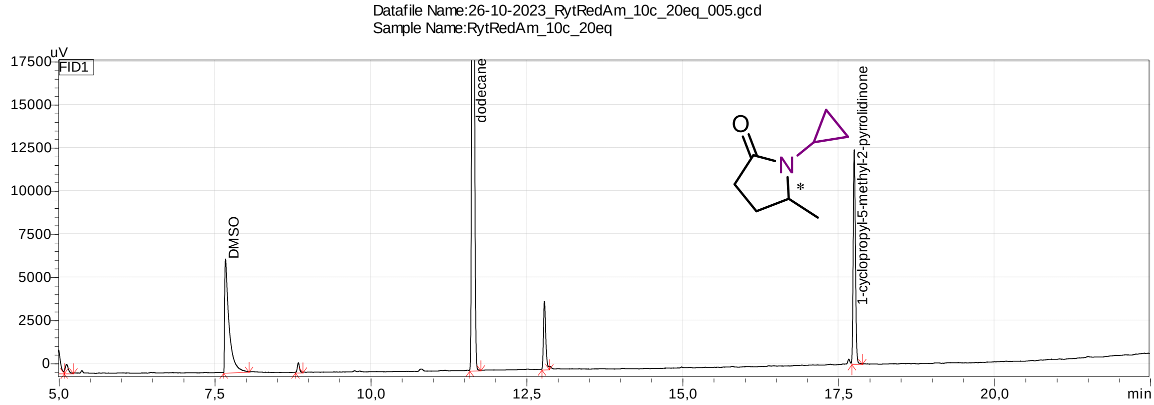


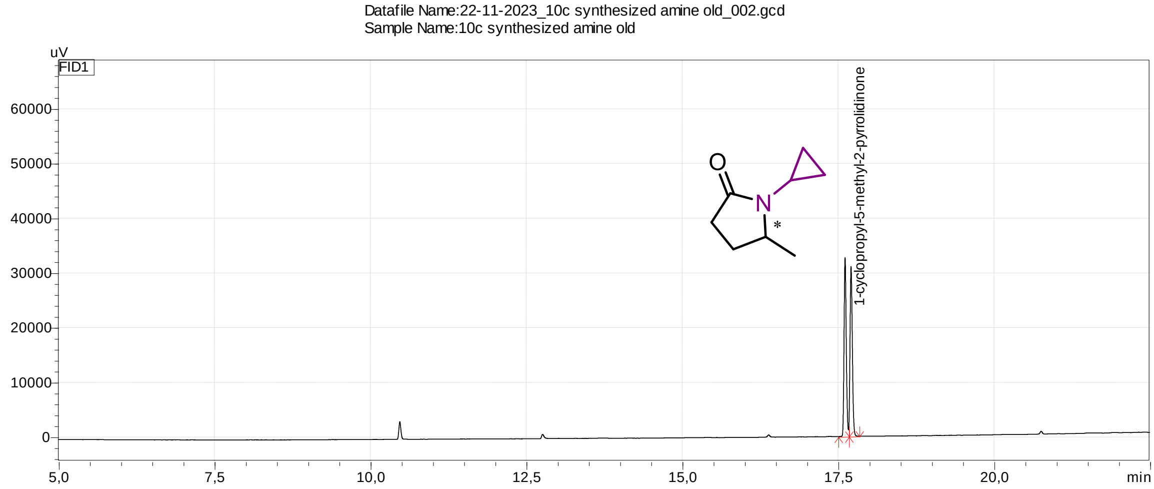


**Figure S32.** GC chromatogram of *Ryt*RedAm reaction product 1-cyclopropyl-5-methyl-2-pyrrolidinone **10c** (top), unknown side-product formed at 12.8 min; bottom: synthesized amine product 1-cyclopropyl-5-methyl-2-pyrrolidinone **10c;** on Hydrodex β-TBDM method C1.

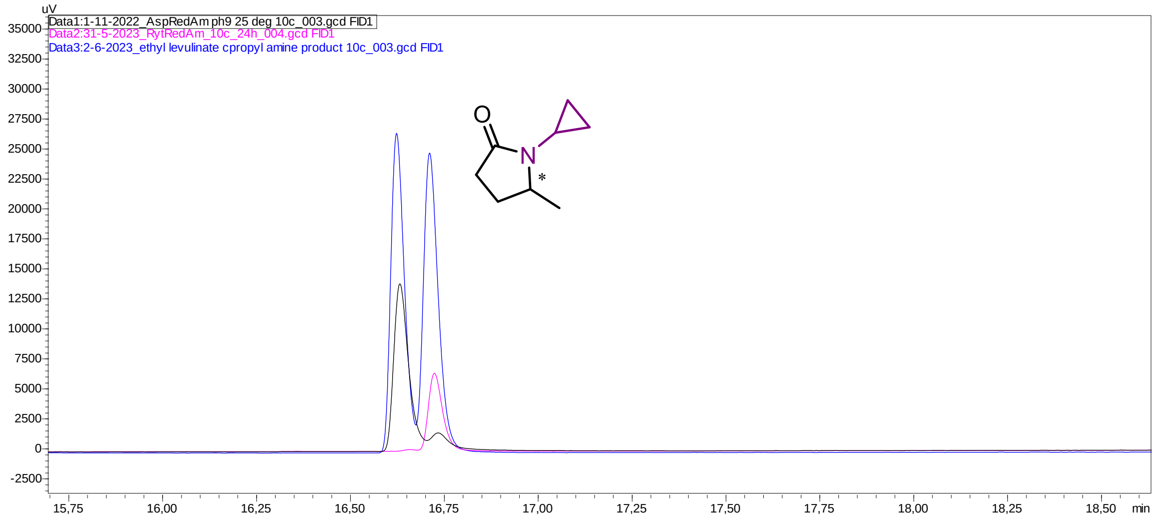


**Figure S33.** Chiral GC chromatograms of the reaction mixture to produce 1-cyclopropyl-5-methyl-2-pyrrolidinone **10c** with *Asp*RedAm (black, 73.9% *ee*), *Ryt*RedAm (pink, 96.6% *ee*), and the synthesized racemic product (blue); on Hydrodex β-TBDM method C1.

## 5.2.6. Amine products from benzaldehyde **11**

### *N*-methylbenzylamine **11b**

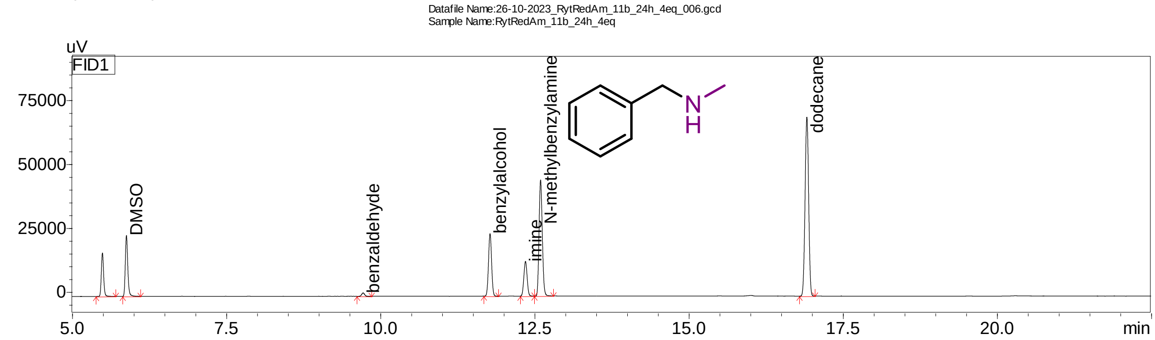


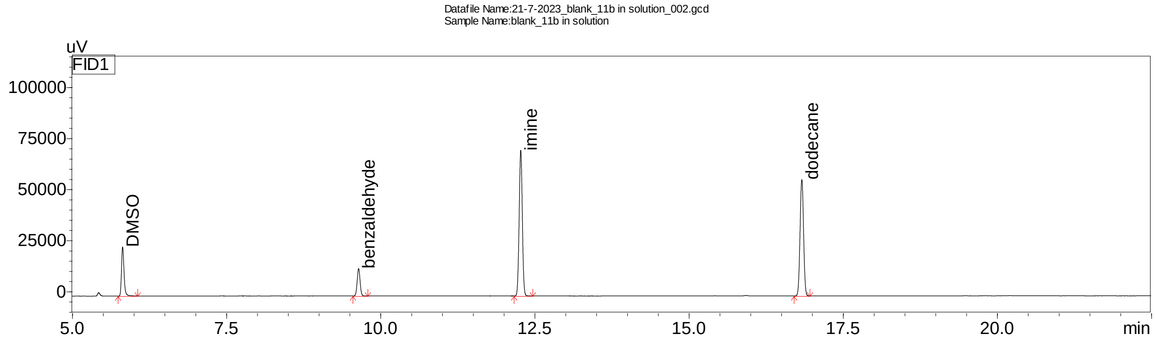


**Figure S34.** GC chromatogram of reaction mixture with *Ryt*RedAm (top) to produce *N*-methylbenzylamine **11b** (12.6 min), the corresponding benzyl alcohol (11.7 min) and imine (12.3 min) peaks were identified by GC-MS. Bottom: control reaction without enzyme, expected imine at 12.3 min; on CP-Sil 8 CB method A3.

### *N*-benzylcyclopropanamine **11c**

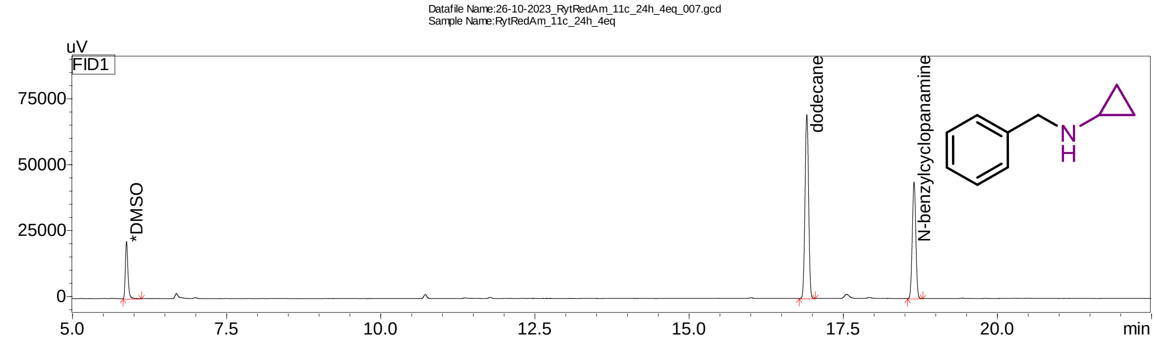


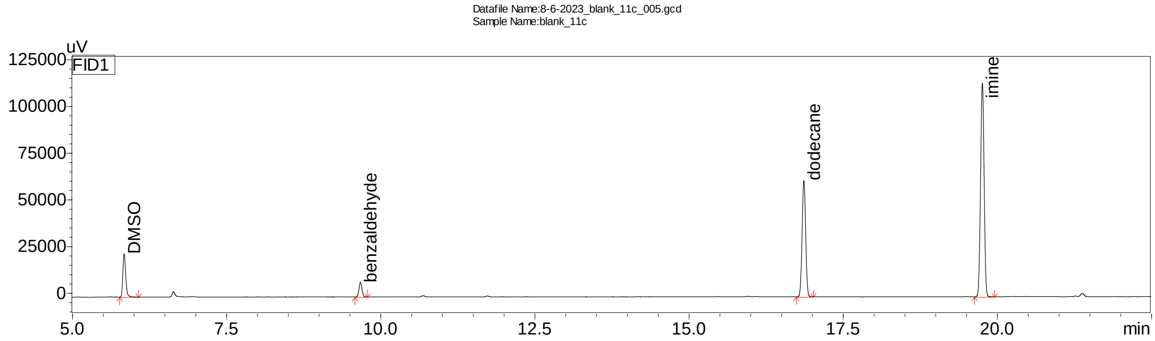


**Figure S35.** GC chromatogram of reaction mixture with *Ryt*RedAm to produce *N*-benzylcyclopropanamine **11c** (18.6 min), confirmed by GC-MS. Bottom: control reaction without enzyme, expected imine at 19.8 min; on CP-Sil 8 CB method A3.

## *N*-benzylpropargylamine **11d**

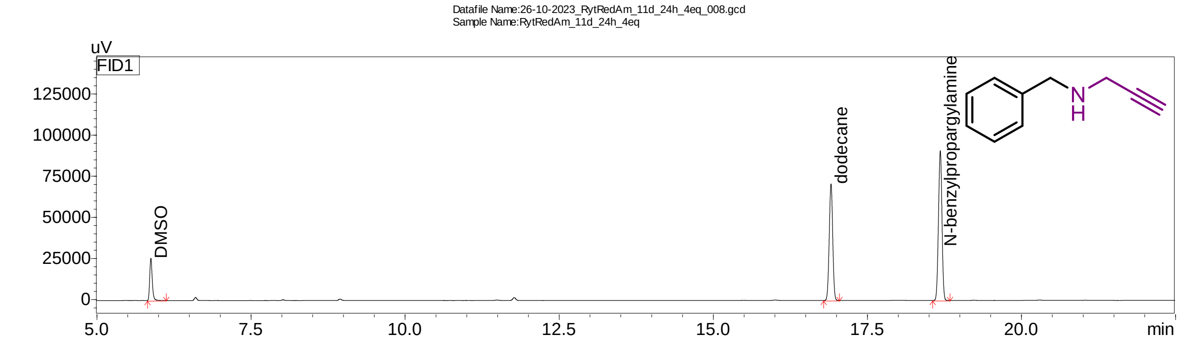


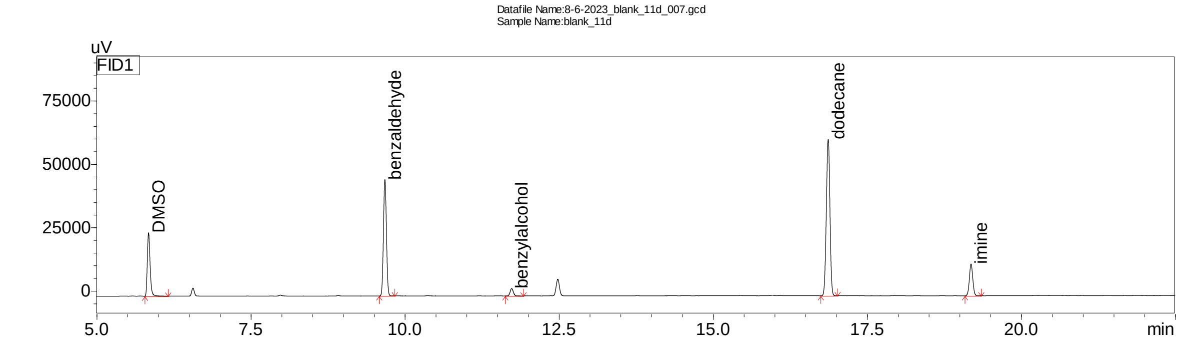


**Figure S36.** GC chromatogram of reaction mixture with *Ryt*RedAm to produce *N*-benzylpropargylamine **11d** (top); bottom: control reaction without enzyme, expected imine at 19.2 min; on CP-Sil 8 CB method A3.

## *N*-allylbenzylamine **11e**

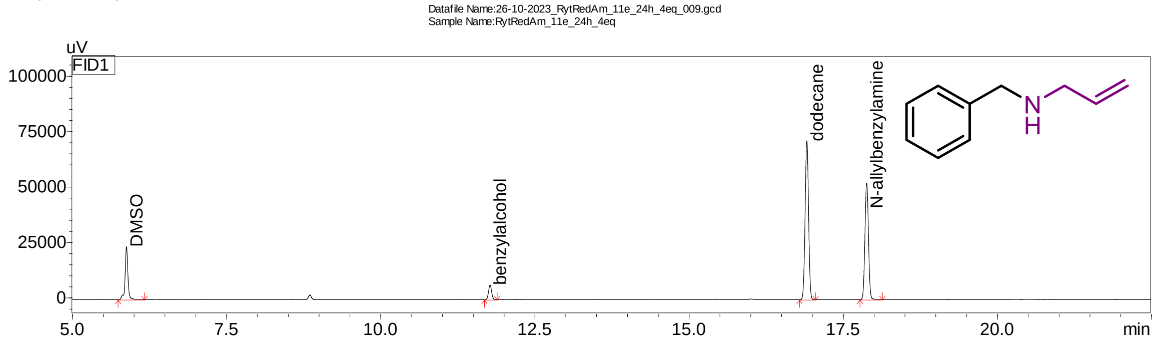


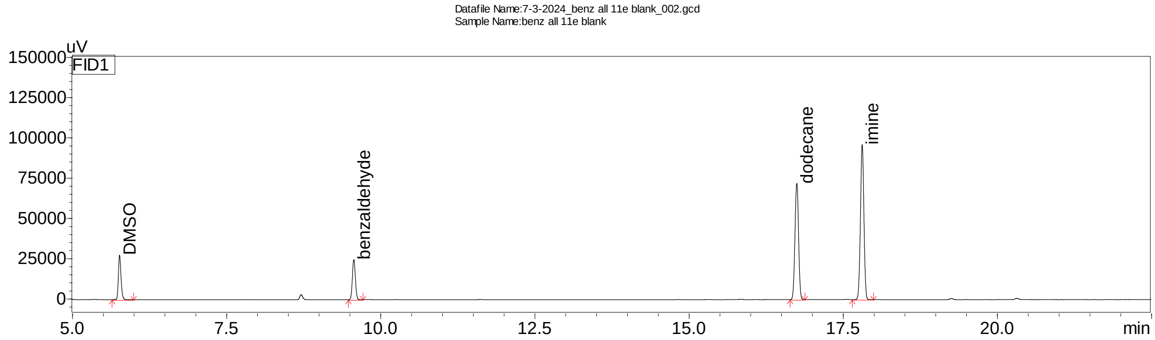


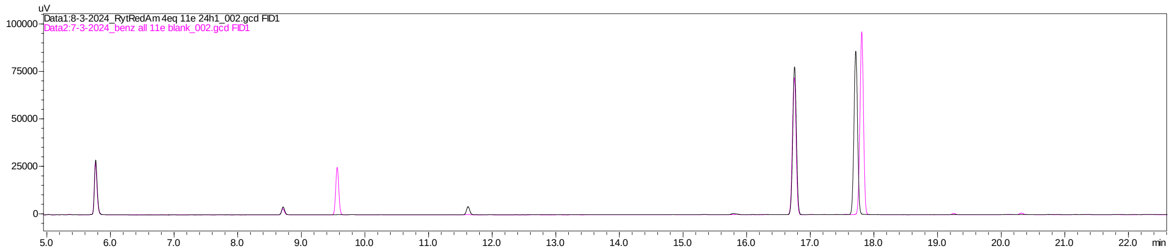


**Figure S37.** GC chromatogram of reaction mixture with *Ryt*RedAm to produce *N*-allylbenzylamine **11e** (17.8 min); middle: reaction control without enzyme, expected imine at 17.9 min; bottom: overlay of chromatograms of the amine produced (black trace) and blank with imine formed (pink trace); on CP-Sil 8 CB method A3.

## dibenzylamine **11f**

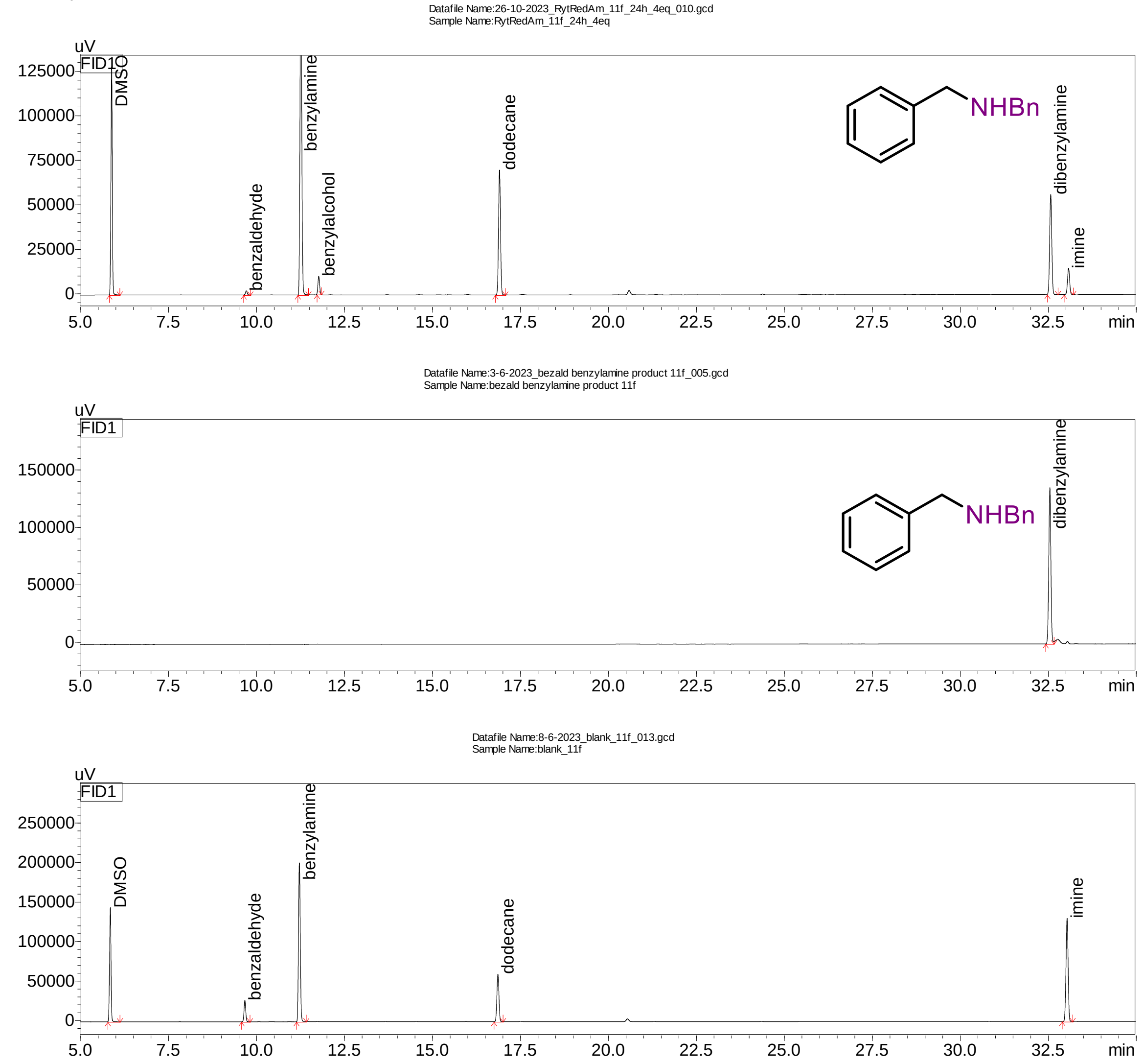


**Figure S38.** GC chromatogram of reaction mixture with *Ryt*RedAm (top) to produce dibenzylamine **11f** (32.5 min); middle: amine standard; bottom: control reaction without enzyme; on CP-Sil-8 CB method A3. Expected imine at 33.1 min.

## 5.2.7. Imine reduction by *Ryt*RedAm

## 2-methyl-1-pyrroline reduction by *Ryt*RedAm

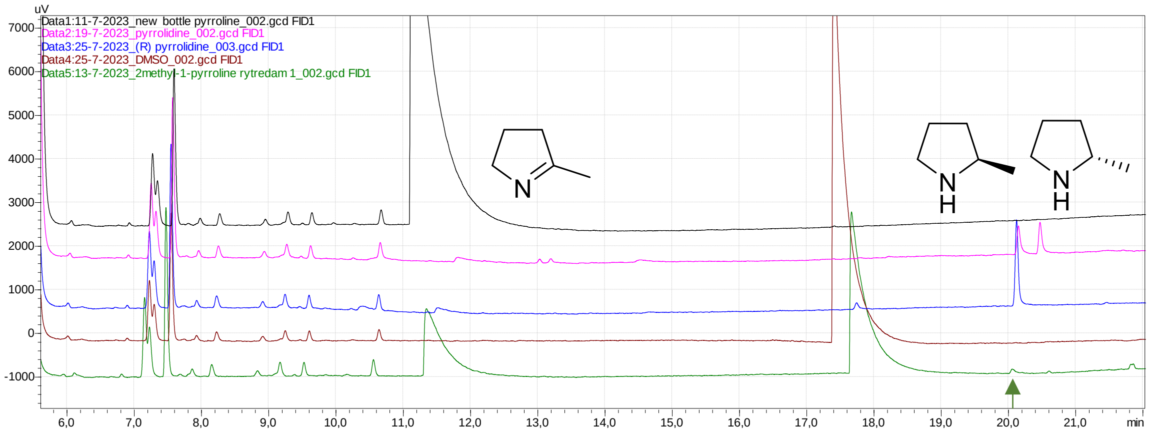


**Figure S39.** GC chromatograms of 2-methyl-1-pyrroline (black), 2-methylpyrrolidine (pink), (*R*)-2-methylpyrrolidine (blue), DMSO (brown) and a reaction mixture with the *Ryt*RedAm (green); on Hydrodex β-TBDM method C2.

## 5.3. GC-MS column and chromatograms

When the expected amine product was not commercially or synthetically available, extracted reaction samples were injected and analyzed on a GC-MS-QP2010 SE from Shimadzu.

**Column D**: CP-Sil-5 VF-1ms, 25 m × 0.25 mm × 0.4 µm, split ratio 50, injection temperature 340 °C, helium as carrier gas.

**Table S5.** GC-MS method and retention times of products which were commercially unavailable.

| | | GC oven program | | | | |
|---|---|---|---|---|---|---|
| **GC column** | **Method** | rate (°C/min), | temp. (°C), | hold (min) | **Compound** | **Ret. time (min)** |
| | | | | | *N*-methylhexylamine **3b** | 3.4 |
| | | | | | *N*-allylhexan-1-amine **3e** | 5.5 |
| **D** | | - | 80 | 3 | *N*-methylbenzylamine **11b** | 5.9 |
| **CP-Sil-5** | D1 | 5 | 100 | 4 | *N*-hexylcyclopropanamine **3c** | 6.0 |
| **VF-1ms** | | 25 | 345 | 1 | *N*-benzylpropargylamine **11d** | 8.9 |
| | | | | | dodecane | 9.0 |
| | | | | | *N*-benzylcyclopropanamine **11c** | 9.5 |

### 5.3.1. *N*-methylhexylamine **3b**

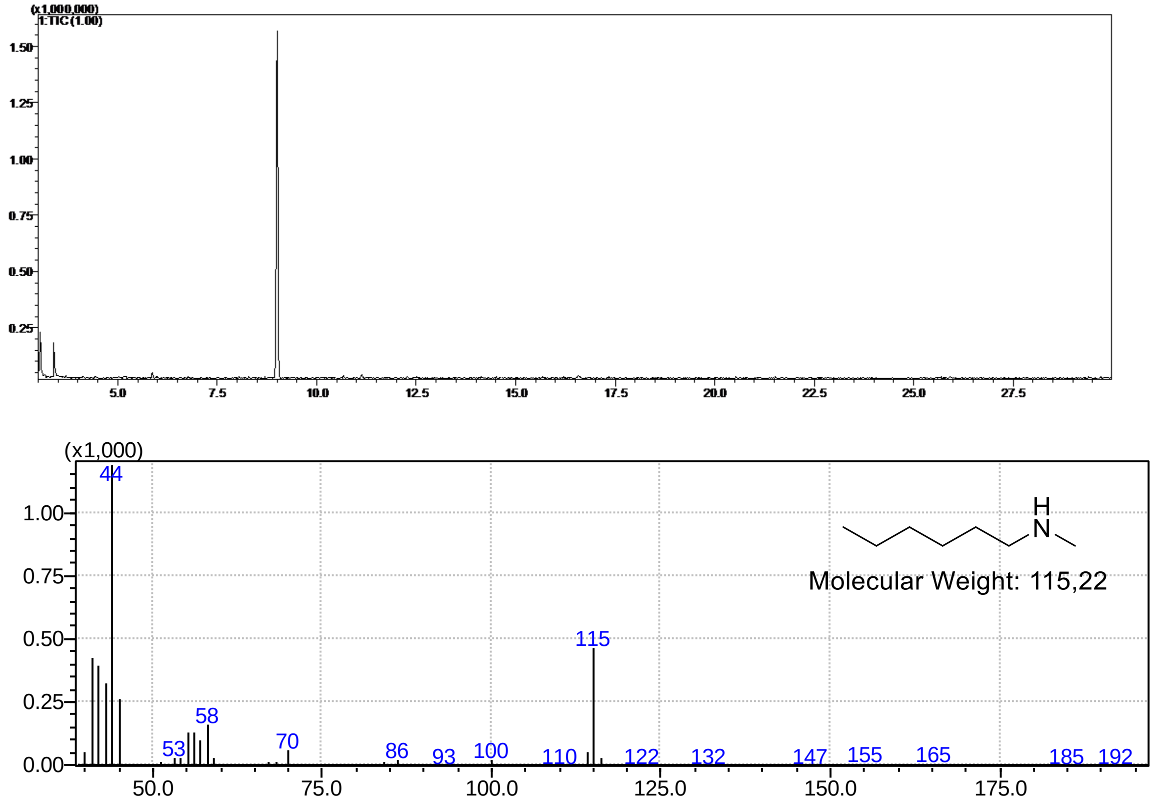


**Figure S40.** GC-MS chromatogram (top) and mass spectrum (bottom) of *N*-methylhexylamine **3b** (3.4 min) on CP-Sil-5 VF-1ms method D1.

## 5.3.2. *N*-hexylcyclopropanamine **3c**

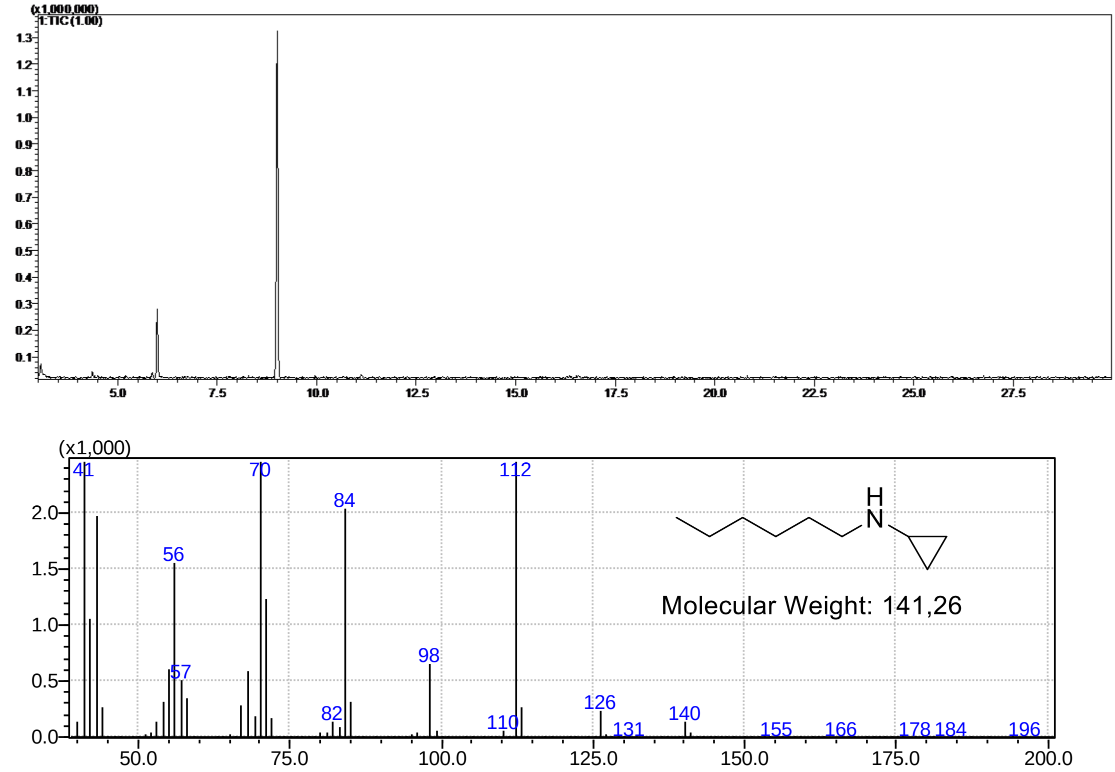


**Figure S41.** GC-MS chromatogram (top) and mass spectrum (bottom) of *N*-hexylcyclopropanamine **3c** (6.0 min) on CP-Sil-5 VF-1ms method D1.

## 5.3.3. *N*-allylhexan-1-amine **3e**

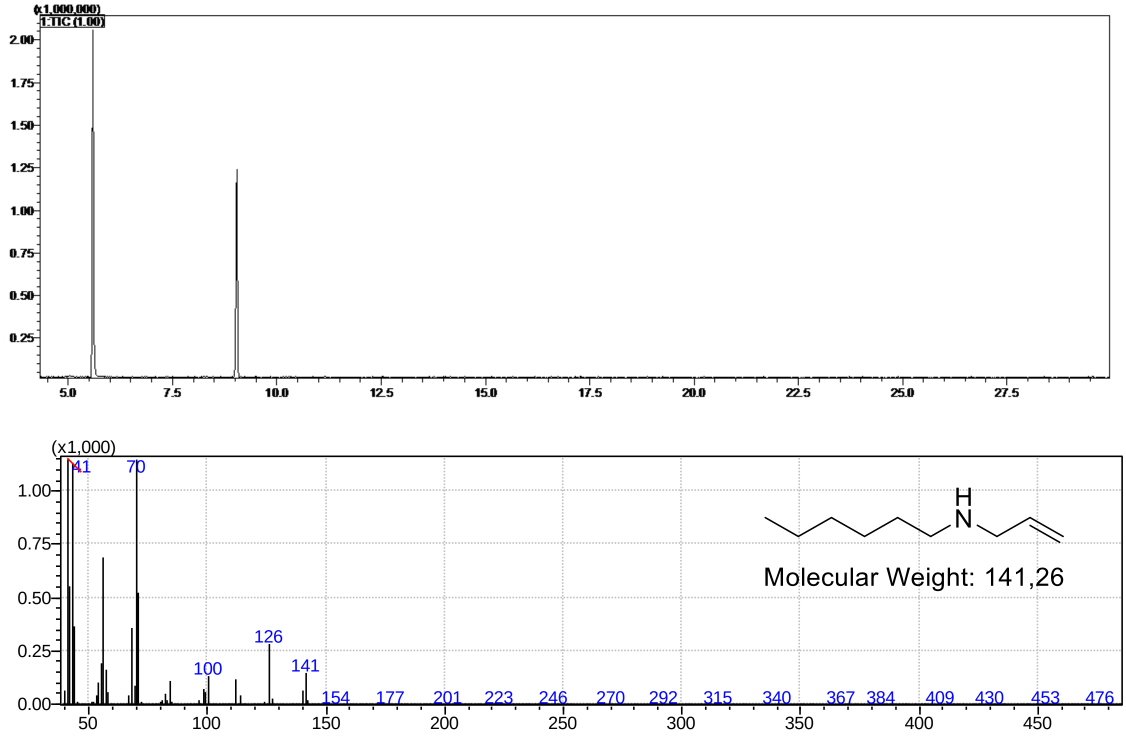


**Figure S42.** GC-MS chromatogram (top) and mass spectrum (bottom) of *N*-allylhexan-1-amine **3e** (5.5 min) on CP-Sil-5 VF-1ms method D1.

### 5.3.4. *N*-methylbenzylamine **11b**

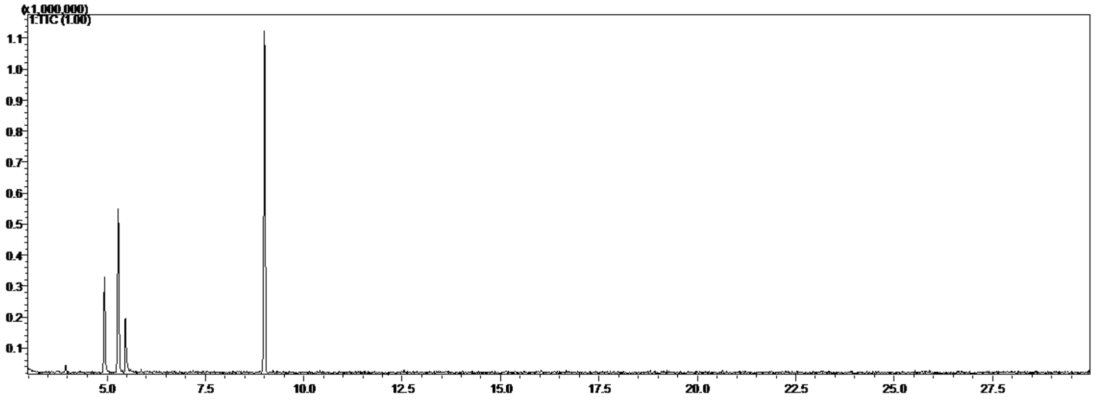

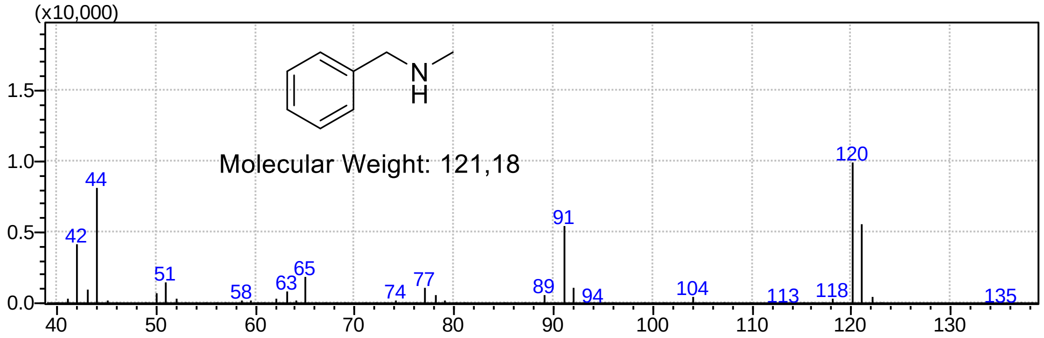


**Figure S43.** GC-MS chromatogram (top) and mass spectrum (bottom) of *N*-methylbenzylamine **11b** (6.0 min) on CP-Sil-5 VF-1ms method D1. Corresponding alcohol present at 4.9 min, corresponding imine present at 5.3 min.

### 5.3.5. *N*-benzylcyclopropanamine **11c**

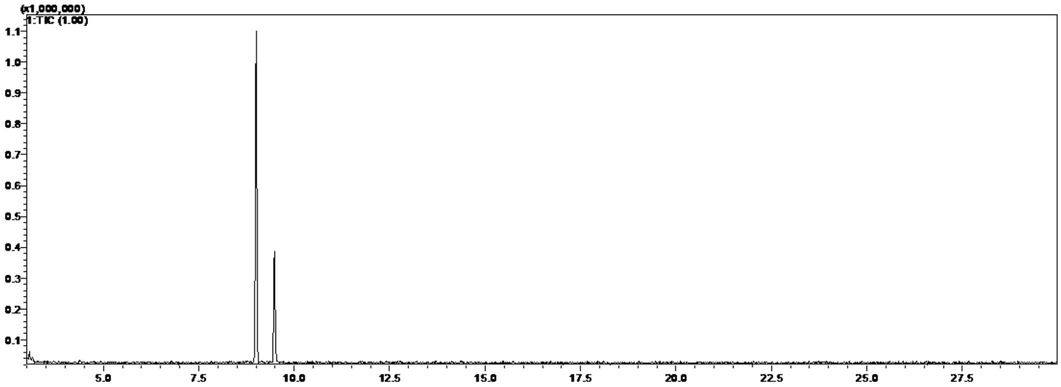

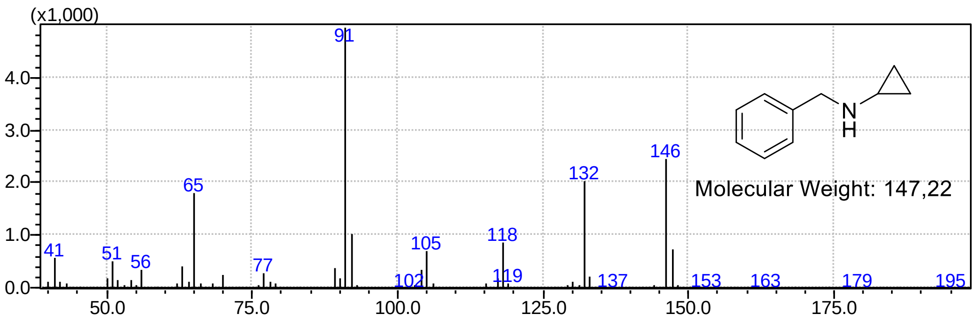


**Figure S44.** GC-MS chromatogram (top) and mass spectrum (bottom) of *N*-benzylcyclopropanamine **11c** (9.5 min) on CP-Sil-5 VF-1ms method D1.

## 5.3.6. *N*-benzylpropargylamine **11d**

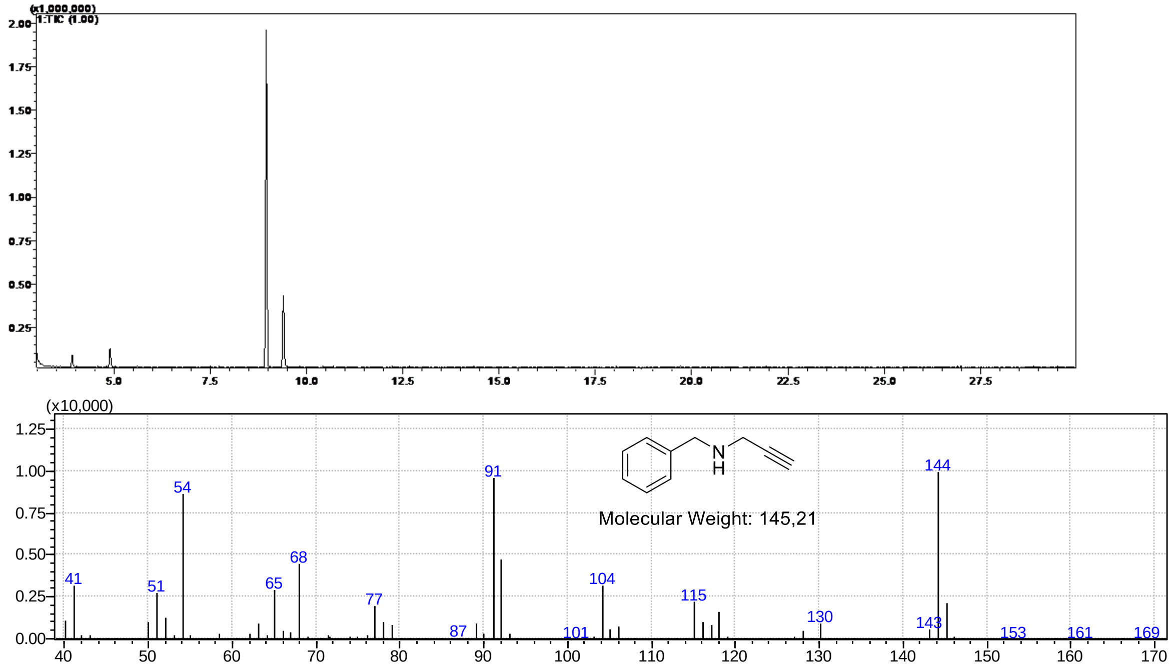


**Figure S45.** GC-MS chromatogram (top) and mass spectrum (bottom) of *N*-benzylpropargylamine **11d** (8.9 min) on CP-Sil-5 VF-1ms method D1.

# 6. *Ryt*RedAm scale-up reactions

## 6.1. Biotransformation to *N*-allylhexan-1-amine 3e

In a 50 mL falcon tube were added 100 mM $KP_i$ buffer pH 7.0, 50 mM hexanal, 200 mM allylamine, 150 mM Glc, 0.2 mM $NADP^+$, 12 U/mL *Bs*GDH, and 1.0 mg/mL *Ryt*RedAm in a total volume of 14 mL. The reaction mixture was shaken at 350 rpm at 20 °C for 24 h. The workup was done as described by Mayol *et al.*,[11] After 24 h, the mixture was basified with 10 M NaOH until pH was 12.0. Then, the product was extracted with 3 × 20 mL $Et_2O$. A solution of 2 M HCl in $Et_2O$ was added (2 mmol) to the combined organic layers. Then, 10 mL of distilled water was added and the corresponding amine hydrochloride salt was extracted with 2 × 20 mL water. The combined aqueous phase was washed with 3 × 10 mL $Et_2O$. The aqueous phase was lyophilized to obtain *N*-allylhexan-1-amine hydrochloride salt **3e** (65 mg, 52% yield) as pale-yellow crystals.

**$^1$H NMR** 400 MHz, $CDCl_3$) 9.58 (br s, 2H), 6.14-6.03 (m, 1H), 5.46 (m, 2H), 3.59 (m, 2H), 2.88 (m, 2H), 1.89-1.81 (m, 2H), 1.38-1.29 (m, 6H), 0.87 (t, *J* = 6.7 Hz, 3H); **$^{13}$C NMR** (100 MHz, $CDCl_3$) 127.8, 123.9, 49.4, 46.4, 31.1, 26.4, 25.8, 22.4, 13.9.

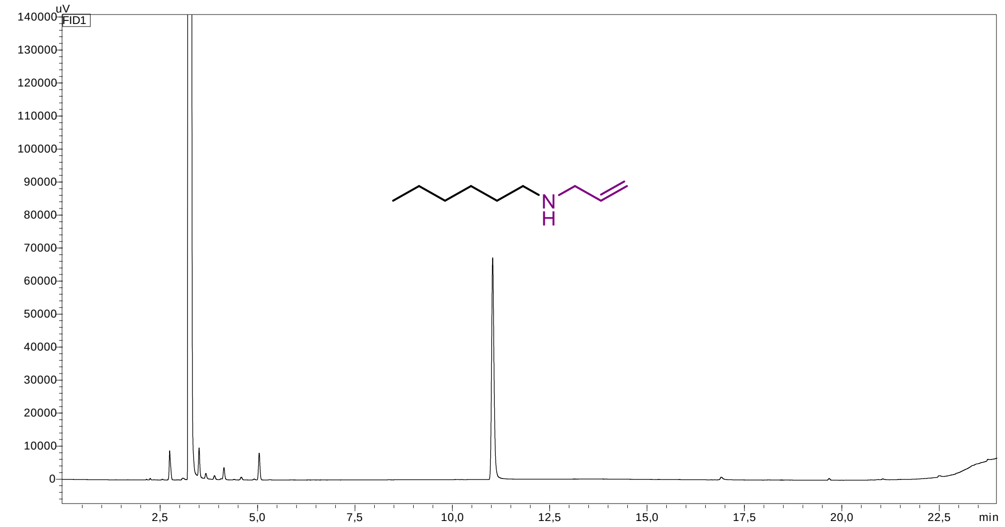


**Figure S46.** GC chromatogram of isolated *Ryt*RedAm reaction product *N*-allylhexan-1-amine **3e** in EtOAc.

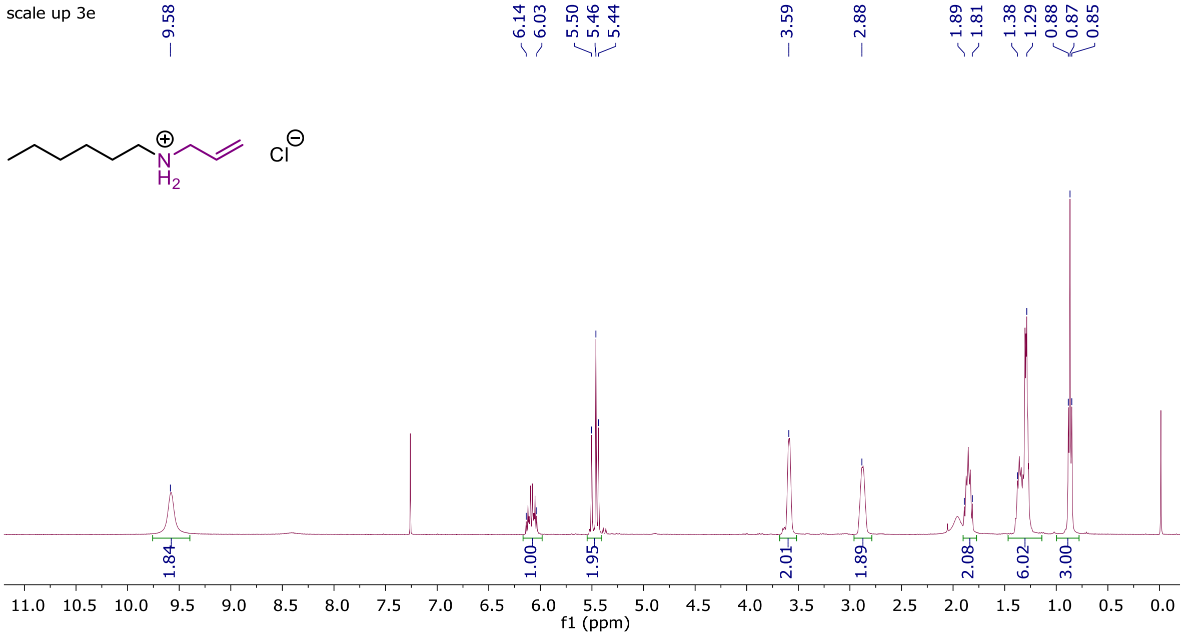


**Figure S47.** $^1$H NMR spectrum in $CDCl_3$ of isolated *Ryt*RedAm reaction product *N*-allylhexan-1-amine hydrochloride salt **3e**-HCl.

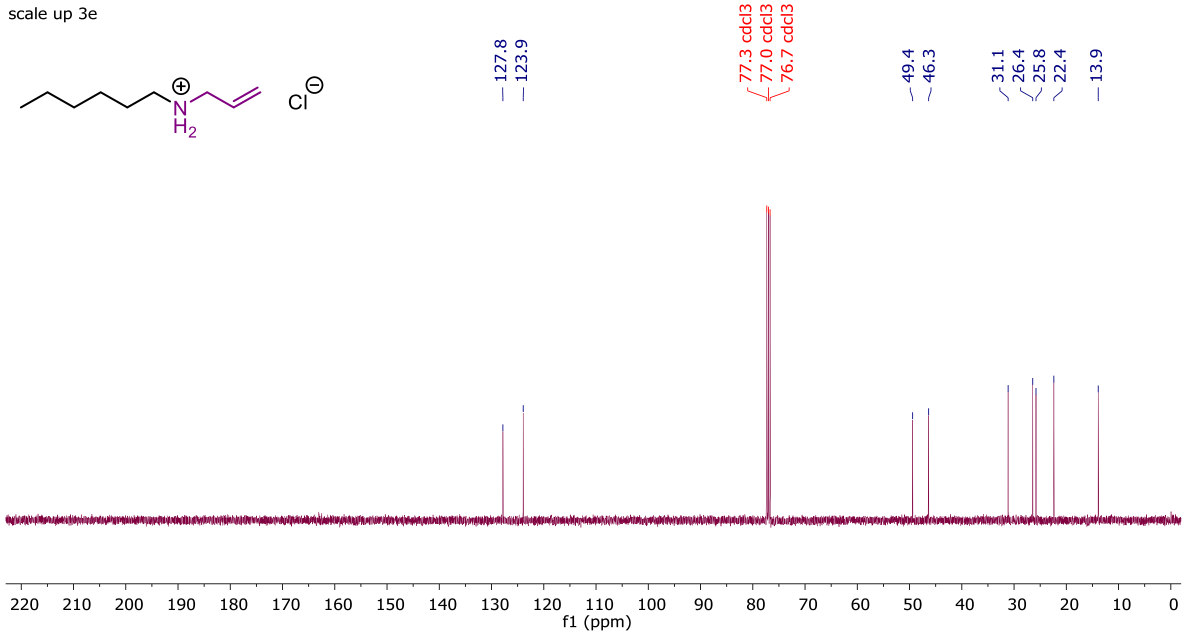


**Figure S48.** $^{13}C$ NMR spectrum in $CDCl_3$ of isolated *Ryt*RedAm reaction product *N*-allylhexan-1-amine hydrochloride salt **3e**-HCl.

## 6.2. Biotransformation to 1-cyclopropyl-5-methyl-2-pyrrolidinone 10c

In a 50 mL falcon tube were added 100 mM $KP_i$ buffer pH 7.0, 50 mM ethyl levulinate, 250 mM cyclopropylamine, 150 mM Glc, 0.2 mM $NADP^+$, 12 U/mL *Bs*GDH, and 1.0 mg/mL *Ryt*RedAm in a total volume of 14 mL. The reaction mixture was shaken at 350 rpm at 20 °C for 24 h. The workup was done as described by Aleku *et al.*[9] After 24 h, the mixture was basified with 10 M NaOH until pH was 12.0. The product was extracted with 3 × 20 mL dichloromethane ($CH_2Cl_2$). The organic layers were pooled, and the $CH_2Cl_2$ was removed under reduced pressure until a small volume remained. The remaining oil was subjected to column chromatography (silica, 100% EtOAc, $R_f$ = 0.20) to afford the corresponding product as a colorless oil (11 mg, 95.3% *ee,* 11% isolated yield).

**$^1$H NMR** δ (400 MHz, $CDCl_3$) 3.60-3.52 (m, 1H), 2.43-2.35 (m, 2H), 2.31-2.23 (m, 1H), 2.14-2.05 (m, 1H), 1.61-1.53 (m, 1H), 1.26 (d, *J* = 6.3 Hz, 3H), 0.97-0.90 (m, 1H), 0.83-0.76 (m, 1H), 0.71-0.62 (m, 1H), 0.55-0.48 (m, 1H). **$^{13}$C NMR** δ (100 MHz $CDCl_3$) 175.8 (C=O), 55.0 (CH), 30.7 ($CH_2$), 26.3 ($CH_2$), 23.0 (CH), 20.0 ($CH_3$), 7.4 ($CH_2$), 4.1 ($CH_2$).

The isolated 1-cyclopropyl-5-methyl-2-pyrrolidinone (**10c**) was dissolved in $CHCl_3$ to a concentration of 10 mg/mL. Specific rotation was determined using a Perkin Elmer Model 343 S Polarimeter at 20 °C, at a wavelength of 589 nm, $[\alpha]_D^{20}$ = –69° (*c* = 1.0, $CHCl_3$).

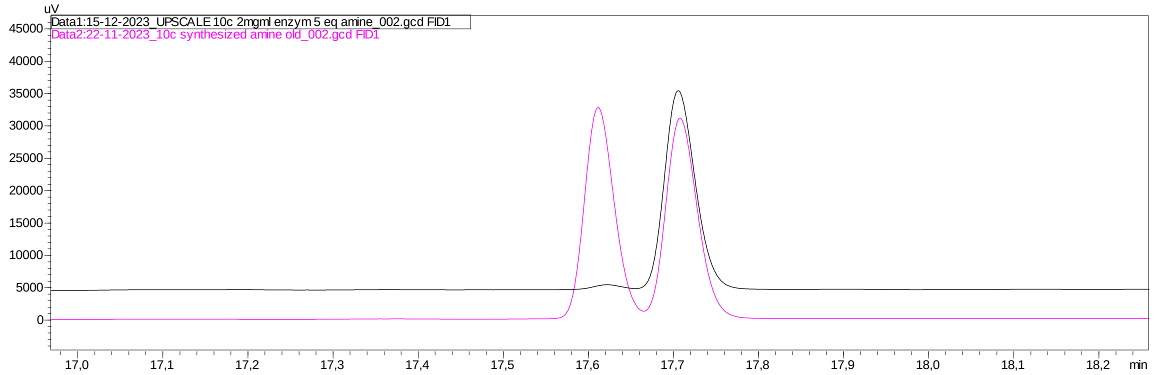


**Figure S49.** GC chromatograms of reaction product 1-cyclopropyl-5-methyl-2-pyrrolidinone **10c** obtained with *Ryt*RedAm (black) and by chemical synthesis (pink); on Hydrodex β-TBDM.

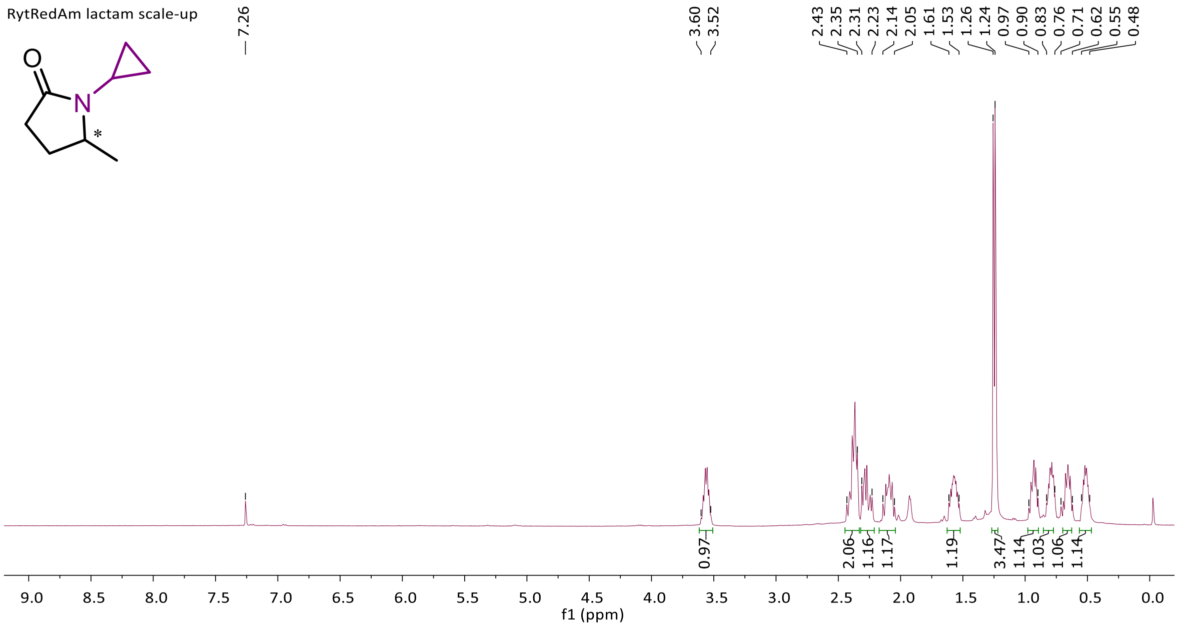


**Figure S50.** $^{1}H$ NMR spectrum in $CDCl_3$ of isolated *Ryt*RedAm reaction product 1-cyclopropyl-5-methyl-2-pyrrolidinone **10c**.

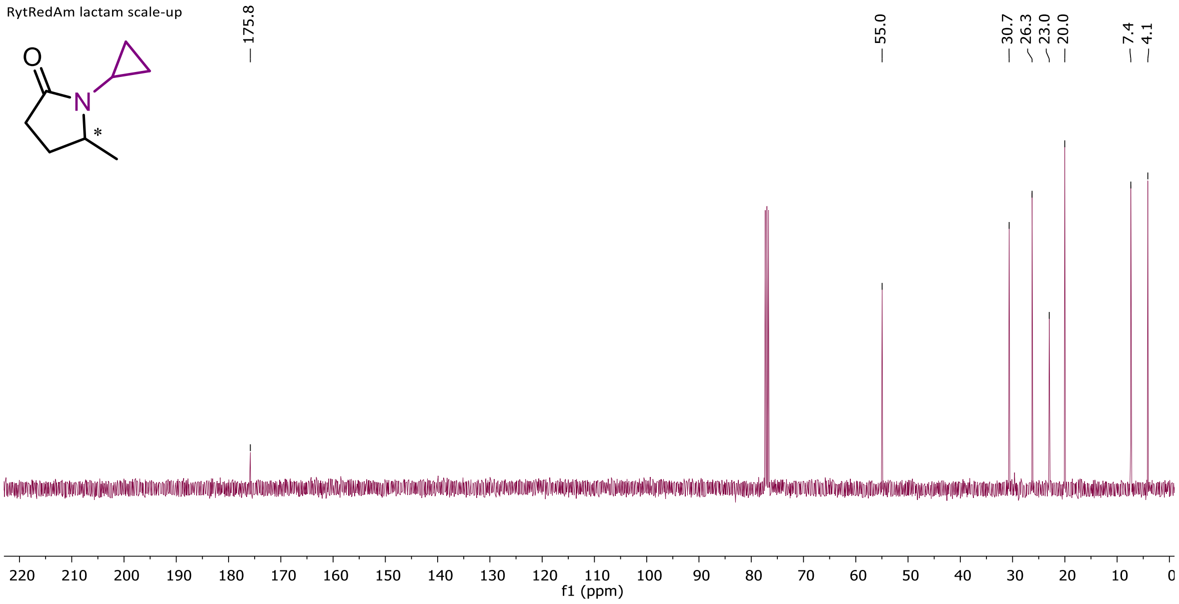


**Figure S51.** $^{13}C$ NMR spectrum in $CDCl_3$ of isolated *Ryt*RedAm reaction product 1-cyclopropyl-5-methyl-2-pyrrolidinone **10c**.

# 7. References

(1) Atkin, K. E.; Reiss, R.; Turner, N. J.; Brzozowski, A. M.; Grogan, G. Cloning, expression, purification, crystallization and preliminary X-ray diffraction analysis of variants of monoamine oxidase from *Aspergillus niger*. *Acta Crystallogr. Sect. F Struct. Biol. Cryst. Commun.* **2008**, *64*, 182-185.

(2) Kabsch, W. XDS. *Acta Crystallogr. D. Biol. Crystallogr.* **2010**, *66*, 125-132.

(3) Evans, P. Scaling and assessment of data quality. *Acta Crystallogr. D Biol. Crystallogr.* **2006**, *62*, 72-82.

(4) Winter, G. *xia2*: an expert system for macromolecular crystallography data reduction. *J. Appl. Crystallogr.* **2010**, *43*, 186-190.

(5) Vagin, A.; Teplyakov, A. MOLREP: an Automated Program for Molecular Replacement. *J. Appl. Crystallogr.* **1997**, *30*, 1022-1025.

(6) Jumper, J.; Evans, R.; Pritzel, A.; Green, T.; Figurnov, M.; Ronneberger, O.; Tunyasuvunakool, K.; Bates, R.; Žídek, A.; Potapenko, A.; Bridgland, A.; Meyer, C.; Kohl, S. A. A.; Ballard, A. J.; Cowie, A.; Romera-Paredes, B.; Nikolov, S.; Jain, R.; Adler, J.; Back, T.; Petersen, S.; Reiman, D.; Clancy, E.; Zielinski, M.; Steinegger, M.; Pacholska, M.; Berghammer, T.; Bodenstein, S.; Silver, D.; Vinyals, O.; Senior, A. W.; Kavukcuoglu, K.; Kohli, P.; Hassabis, D. Highly accurate protein structure prediction with AlphaFold. *Nature* **2021**, *596*, 583-589.

(7) Emsley, P.; Cowtan, K. Coot: model-building tools for molecular graphics. *Acta Crystallogr. D. Biol. Crystallogr.* **2004**, *60*, 2126-2132.

(8) Murshudov, G. N.; Vagin, A. A.; Dodson, E. J. Refinement of macromolecular structures by the maximum-likelihood method. *Acta Crystallogr. D. Biol. Crystallogr.* **1997**, *53*, 240-255.

(9) Aleku, G. A.; France, S. P.; Man, H.; Mangas-Sánchez, J.; Montgomery, S. L.; Sharma, M.; Leipold, F.; Hussain, S.; Grogan, G.; Turner, N. J. A reductive aminase from *Aspergillus oryzae*. *Nat. Catal.* **2017**, *9*, 961-969.

(10) Ziegenhorn, J.; Senn, M.; Bucher, T. Molar absorptivities of β-NADH and β-NADPH. *Clin. Chem.* **1976**, *22*, 151-160.

(11) Mayol, O.; Bastard, K.; Beloti, L.; Frese, A.; Turkenburg, J. P.; Petit, J.-L.; Mariage, A.; Debard, A.; Pellouin, V.; Perret, A.; de Berardinis, V.; Zaparucha, A.; Grogan, G.; Vergne-Vaxelaire, C. A family of native amine dehydrogenases for the asymmetric reductive amination of ketones. *Nat. Catal.* **2019**, *2*, 324-333.
